# Characterization of Carbon Nanostructures by Photoelectron Spectroscopies

**DOI:** 10.3390/ma15134434

**Published:** 2022-06-23

**Authors:** Giorgio Speranza

**Affiliations:** 1 Center for Materials and Microsystems—Fondazione Bruno Kessler, v. Sommarive 18, 38123 Trento, Italy; speranza@fbk.eu; 2 Istituto Fotonica e Nanotecnologie—Consiglio Nazionale delle Ricerche, CSMFO Lab., Via Alla Cascata 56/C Povo, 38123 Trento, Italy; 3 Department of Industrial Engineering, University of Trento, v. Sommarive 9, 38123 Trento, Italy

**Keywords:** XPS, UPS, carbon nanostructures, surface characterization, electronic properties

## Abstract

Recently, the scientific community experienced two revolutionary events. The first was the synthesis of single-layer graphene, which boosted research in many different areas. The second was the advent of quantum technologies with the promise to become pervasive in several aspects of everyday life. In this respect, diamonds and nanodiamonds are among the most promising materials to develop quantum devices. Graphene and nanodiamonds can be coupled with other carbon nanostructures to enhance specific properties or be properly functionalized to tune their quantum response. This contribution briefly explores photoelectron spectroscopies and, in particular, X-ray photoelectron spectroscopy (XPS) and then turns to the present applications of this technique for characterizing carbon nanomaterials. XPS is a qualitative and quantitative chemical analysis technique. It is surface-sensitive due to its limited sampling depth, which confines the analysis only to the outer few top-layers of the material surface. This enables researchers to understand the surface composition of the sample and how the chemistry influences its interaction with the environment. Although the chemical analysis remains the main information provided by XPS, modern instruments couple this information with spatial resolution and mapping or with the possibility to analyze the material in operando conditions at nearly atmospheric pressures. Examples of the application of photoelectron spectroscopies to the characterization of carbon nanostructures will be reviewed to present the potentialities of these techniques.

## 1. Introduction

The characteristics of a material refer to a list of properties that depend on the electronic structure of the solid. This, in turn, is intimately bound to the structure and chemical properties of the material. Crystalline or amorphous phases, size confinement and the material chemical composition strongly influence the charge distribution around atoms. Thus, an accurate description of the material properties requires a precise characterization of these parameters. Generally, this is done using a list of complementary techniques which may be roughly classified in two groups: those using photons as probes and those relying on electrons. Here, we will focus our attention to the photon probes and, in particular, X-ray photons in order to investigate the material properties. 

**Figure 1:**
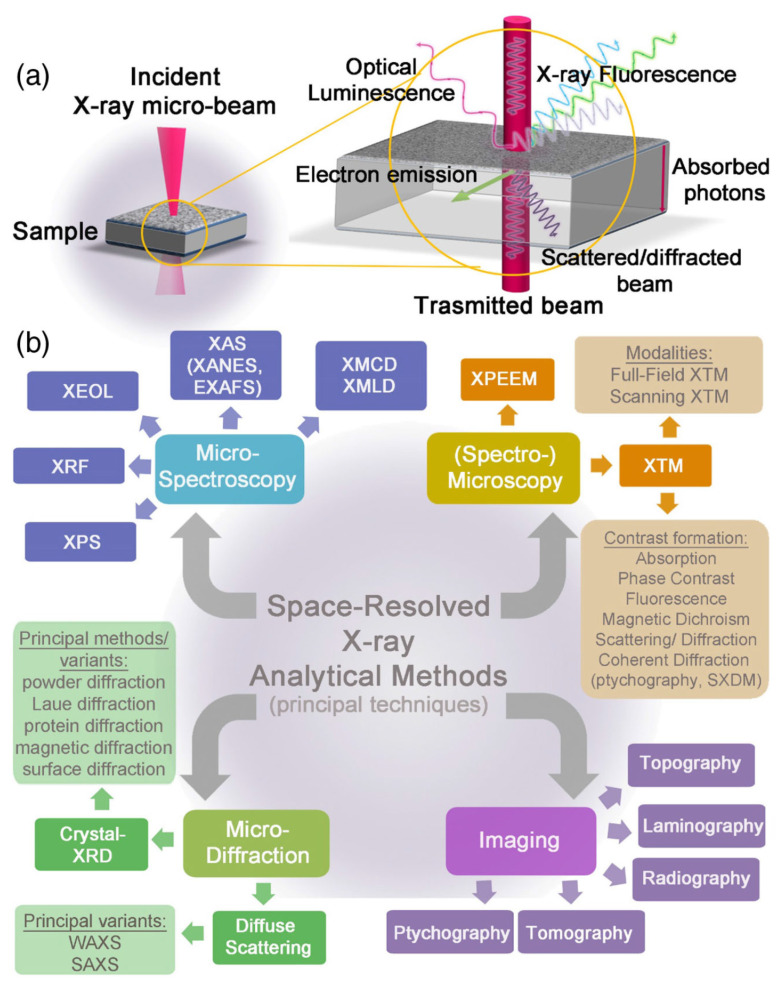
(**a**) Schematic representation of the principal X-rays and matter interactions. (**b**) Classification of the most popular X-ray-based space-resolved analytical tools with micrometric and submicrometric resolutions; four main categories are represented: micro-spectroscopy, micro-diffraction, spectro-microscopy and imaging. For graphical reasons, only the “micro-” prefix is reported, although, nowadays, the majority of these analytical techniques can provide information at the submicrometric scale. The most common variants, methods, and operation modalities in each category are reported as well. Reprinted with permission from [11].

The term X-ray is used to indicate a radiation with wavelengths in the range 10 nm–0.01 nm, corresponding to soft and hard rays. Different kinds of light/matter interactions occur at different frequencies of X-ray photons. This led to the development of a number of techniques to probe materials at different length scales, from the macroscopic to the atomic level. In the first case, bulk structural and chemical information are provided, while in the second, a description of the local environment of the atoms is obtained. A schematic representation of the X-ray photon/matter interactions and the corresponding analytical techniques are summarized in Figure 1.

Among the techniques based on X-radiation, we can mention X-ray diffraction, X-ray based tomography, Extended X-ray Absorption Fine Structure and X-ray Absorption Near-Edge Spectroscopy, X-ray Fluorescence and, finally, X-ray Photoemission Spectroscopy (XPS).

In the first class of these techniques, both probing and detection are based on X-rays (diffraction, wide- and small-angle scattering, absorption, fluorescence). In the second class, X-ray photons are used as probes while photoelectrons are detected. X-rays are used to analyze the electronic structure of the material, and this reflects the structural and chemical properties of the sample. 

This is very useful when the dimensions of the sample are reduced on the nanoscale. In nanosized systems, quantum effects come into play, inducing radical changes in the material’s properties. Understanding the different behaviors of the nanostructures with respect to their parent bulk materials requires the use of different techniques providing complementary information. XPS can probe the changes of the electronic structure reducing the system dimensions, thus shedding light on the development of the traits induced by the quantum confinement. 

Recently, the scientific community has been involved in two game-changing innovations: the synthesis of the single sheet of carbon atoms—namely, the graphene—and the development of quantum technologies. Both these events have had a great impact on the scientific research boosting the development of novel technologies based on the peculiar properties induced by the quantum confinement. Single graphene sheets and graphene coupled to other carbon nanostructures were used in a plethora of applications in different areas from energy and chemistry to sensing, biomedicine, etc. [1,2]. As for the quantum technologies, they rely on the possibility to initialize a quantum system into a well-known state and to read out its state after the system has interacted with an external entity. This may be utilized for sensing or measuring physical quantities, producing single photon sources and entangled photons [3,4]. Diamonds and nanodiamonds and their defects (N, Si, Ge, vacancies) are among the materials offering high emission coherence and lifetimes long enough to be used in quantum devices. 

These events add to the rich list of milestones [5,6] where new forms of carbon and carbon nanostructures came to the fore in the panorama of scientific research. This motivates the focus of the attention on carbon nanostructures and how they can be characterized using XPS to provide a piece of information still lacking in the literature.

XPS can be used to characterize any kind of material, provided it can be placed under ultra high vacuum (UHV), although recent XPS instruments work in near ambient pressures [7], enabling the liquid and gaseous phases at high pressures and the materials in operando conditions to be analyzed [8]. Furthermore, the recent evolution of XPS instruments allow for the characterization of materials with lateral resolution on the micron scale obtained by acting on the X source [9] or on the analyzer performances [10]. XPS is commonly utilized to characterize the chemical composition of nanostructures. This review will direct the attention toward a different direction and, in particular, to the spectral changes induced by the reduction of the system dimensions to the nanoscale. In the next sections, we will describe how the macro-to-nano transition induces variations of the electronic structure of the nanoparticle, which can be detected by XPS. Then, some examples showing the use of XPS for characterizing carbon nanostructures will be provided. 

### 1.1. X-ray Photoelectron Spectroscopy

Photoelectron spectroscopy is performed by measuring the number of electrons emitted from the surface of a sample as a function of their kinetic energy (E_k_). The photoemission process results from the absorption of a monochromatic photon of energy hν_0_ and a complete transfer of its energy to the core level electron. To perform photoelectron spectroscopy, the sample is then irradiated with the light of selected wavelengths to generate photoelectrons, whose energy is measured using a hemispherical energy analyzer. The phenomenon is described by Einstein’s formula for the photoelectric effect [12,13,14,15,16].

E_k_ = hν_0_ − Φ (1)

where Φ represents the sample work function, the energy difference between the Vacuum level E_vac_ and the Fermi level E_F_. In metals, this corresponds to the minimum energy required to remove an electron and can be obtained by measuring its kinetic energy E_k_ when hν_0_ is known. Equation (1) describes the emission of electrons from the highest occupied molecular orbital (HOMO) orbitals. Photoemission also occurs from inner core levels and is described by the relation

E_k_ = hν_0_ − BE − Φ (2)

where BE corresponds to the energy required to excite the electron to E_F_. Depending on the type of the probing radiation utilized, we are dealing with X-ray photoelectron spectroscopy, where the photon energy is commonly between 1–6 keV [16,17,18], or ultraviolet photoelectron spectroscopy (UPS), where photon energy ranges from 5 to 48 eV [19,20]. Generally, in photoelectron spectroscopy, the Koopmans Theorem is applied—namely, the first ionization energy of a system equals the negative of the energy of the HOMO energy −ε_0_. In this approximation, the rearrangement of the charge distribution occurring after photoemission and the effects deriving from the electron correlation are neglected. According to Equation (1), XPS provides the Φ values (i.e., −ε_0_) of the HOMO. This relation is extended also to the core orbitals of the atoms present at the sample surface. 

Since each atom possesses its own electronic structure, the values of ε_0_ are element-specific such that XPS can be used for the elemental speciation. In addition, since the integrated intensity of the XPS peaks is proportional to the number of emitting atoms, XPS can provide the element concentration of the analyzed sample [21,22]. However, different electronic structures result in different element cross sections. For a homogeneous solid composed of atoms A with density *N_A_* and illuminated by X-ray photons of energy *hν* and intensity *I_hν_*(α, z) (α = X-ray incidence angle) at z depth, it is possible to describe the intensity *I_A,i_* of the photoelectron current generating ionization of a core-level *i* (*i* = 1s, 2p, 3d, etc.) of element A considering the integral of the spatial distribution of excitation and emission [23,24]:

*I_A,i_* = ΔΩ/4π _0_∫^∞^*I_h__ν_*(α, z) σ_A,i_ W_A,i_(β_A,i_ Ψ) *N_A_*(z) exp[−z/(λ_A,E_ sinθ)] dz (3)

ΔΩ is the acceptance solid angle of the XPS analyzer, σ_A,I_ is the ionization cross section of the orbital i of element A, W_A,i_(β_A,i_ Ψ) is the angular asymmetry factor [25] and the remaining term describes the attenuation of the photoelectron signal generated at depth z (see next section). The cross section σ_A,i_ was calculated by Scofield [26], treating electrons relativistically in a Hartree–Slater central potential for all elements. The Scofield cross sections do not consider screening effects leading to intrinsic plasmon losses. More precise evaluation of the elemental sensitivity factors (RSFs) were obtained experimentally by Wagner [27] and, more recently, by other authors [28] using uniform standards and standardized background subtraction. The direct use of the RSF is possible only if the user possesses the same XPS instrument as those of the works listed [27,28]. In other cases, the different analyzer transmission function and the detector efficiency at various BEs may introduce consistent errors. Generally, the instrument manufacturer provides RFSs calibrated for the instrument analyzer/detector. 

By comparing the spectra of the same element in different environments, it is possible to realize the presence of peculiar differences. In particular, it was observed that the different chemical bonds formed by an element with other chemical species strongly affect its core line spectrum. Then, XPS offers the possibility to describe the surface chemistry of the material. For this reason, XPS was originally called electron spectroscopy for chemical analysis (ESCA). 

### 1.2. Surface Sensitivity of the Photoelectron Spectroscopies

As these spectroscopies are based on the detection of electrons photoemitted in the sample matrix, they are surface-sensitive since they probe the first few monolayers of the sample. This is the result of the energy loss scattering processes occurring along the travel of the electron towards the surface. The electron kinetic energy E_k_ is completely dissipated if electrons are generated deep in the material, thus preventing their ejection. A consequence of this fact is the possibility of varying the sampling depth, changing the energy of the excitation source. This can be done in synchrotron radiation facilities where it is possible to select the photon energy in a wide range (from ~10 eV to tens of KeV). Selecting photons with increasing energy increases E_k_ and the probability that deep photoelectrons arrive to leave the surface. Conversely, by lowering the excitation energy by using UV photos, the analysis is confined to the very top material layers. For an electron created at a depth z below the surface, the probability to be ejected is linked to the inelastic scattering. This leads to electron attenuation, which follows the Beer–Lambert law, similarly to what happens to photons in an absorbing medium:

I_z_ = I_0_ exp(−z/λ sinθ) (4)

where I_z_ is the intensity of the electron current created by atoms at depth z, I_0_ is the maximum intensity from the surface atoms and θ is the take-off angle, defined by the electron trajectory and the sample surface. Here, λ represents the inelastic mean free path, representing the average distance covered by an electron between two successive elastic and inelastic collisions [29]. The attenuation length depends not only on the material but also on the kinetic energy of the photoelectrons. The sources of the attenuation length were studied in the past by Seah and Dench [30], collecting experimental attenuation length values obtained from overlayers deposited on substrates. If a sample is formed by an overlayer A of thickness *d* and a bulk B, then we can describe the contributions to the photoelectron intensities of A and B as

I_A_ = I^∞^_A_ [1 − exp(−*d*/λ_A_ sinθ)] (5)

I_B_ = I^∞^_B_ [exp(−*d*/λ_B_ sinθ)] (6)

I_A_ and I_B_ are the detected intensities deriving from A and B, I^∞^_A_ and I^∞^_B_ are the signal intensities that would be generated by a sample formed just by A and B and λ_A_ and λ_B_ are their attenuation lengths. θ is the take-off angle defined by the sample surface and the analyzer axis. By applying Equations (5) and (6), the authors obtained λ values expressed in monolayers. Using a “universal curve”, the authors fitted the values of the attenuation length separately for elements and organic and inorganic compounds. The general form of the universal curve is

λ = [a/E^2^ + b (*d* E^0.5^)] (7)

For energies between 1 and 10,000 eV above the Fermi level, a = 538, b = 0.41 and *d* is the thickness of the monolayer, expressed as

*d* = A/ρnN × 10^24^ (8)

**Figure 2:**
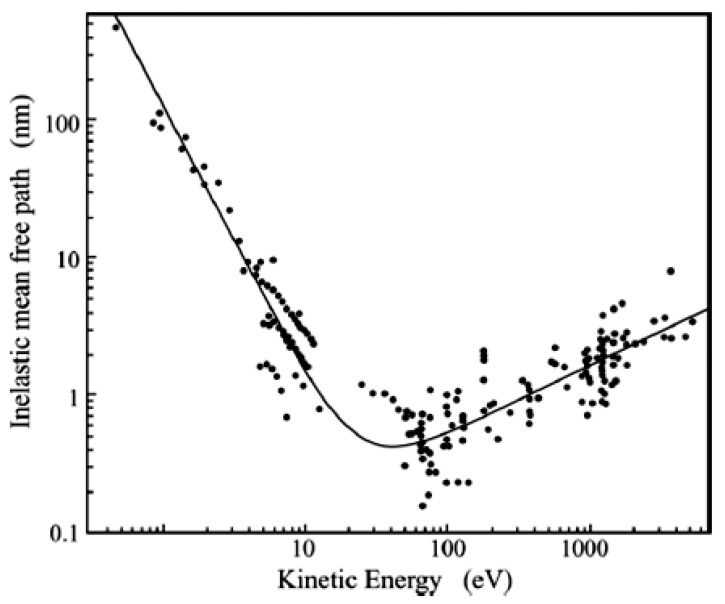
Compilation of results for the attenuation length as a function of electron energy for elements. Reproduced with permission from [30].

The experimental data fitted with the universal curve for elements is represented in Figure 2. Here, A is the atomic or molecular weight, n is the number of atoms in the molecule, N is Avogadro’s number and ρ is the bulk density in kg m^−3^. Fitting Equation (9) for inorganic compounds gives a = 2170 and b = 0.72, while for organic molecules, a = 49 and b = 0.11.

The trend of the attenuation length as a function of the energy can be utilized to vary the sampling depth, which is commonly taken as 3λ. The photon energy varies from few eV when using ultraviolet sources (generally, He sources emitting HeI at 21.2 eV and HeII at 40.8 eV photons) to 1253.6 eV or 1486.6 eV for Mg and Al Kα anodes, which are generally utilized in the X-ray sources of XPS instruments. Using synchrotrons to perform photoelectron spectroscopy, the radiation energy is varied in a broad range from 10 to 10^4^ eV. Then, the sampling depth varies from fractions of nanometers when using UV to some nanometers in the case of X-ray photons or up to ~20 nm using hard X-ray photons (depending on the material).

### 1.3. Angle-Resolved XPS

**Figure 3:**
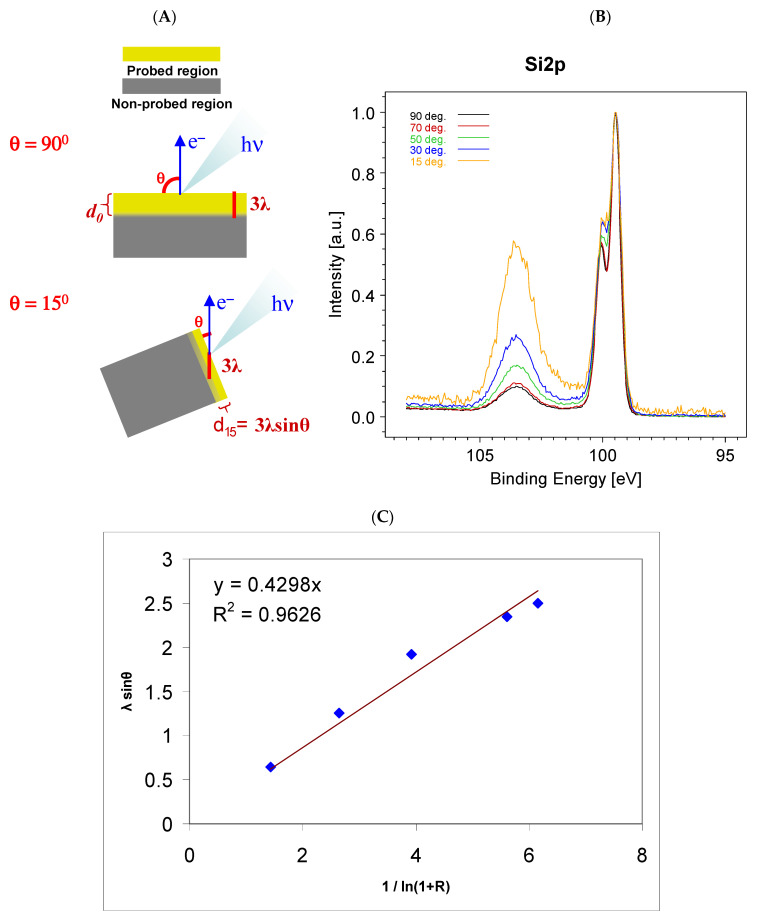
(**A**) at tilt = 0°, the sampling depth d_0_ corresponds to three times the attenuation length λ. At tilt angle 75° (take-off angle = 15°), the real sampling depth d_75_ is reduced to 3λ sinθ. The increased surface sensitivity is shown in (**B**), where the intensity of the components of the Si 2p core line representing the native silicon oxide at ~103 eV increases as the tilt angle θ increases from 0° to 75°. (**C**) Linear regression of the experimental intensities obtained from the spectra in (**B**). From Equation (11), a value of d = 0.43 nm is obtained. Figures produced by the manuscript author.

The attenuation of the photocurrent described by the Lambert–Beer law (4) enables the non-destructive depth profiling of the sample surface. Equations (5) and (6) describe the variation of the electron photocurrent as a function of the attenuation length and the take-off angle. In a simple case, it is possible to analyze the changes of different “bond-components” of a core line by varying the take-off angle as shown in Figure 3A. A well-known example is the estimate of the thickness of silicon native oxide. Equations (5) and (6) are reduced to

λ_B_ sinθ = d/ln(1 + I_SiO2_/I_Si_) = λ_B_ sinθ = d/ln(1 + R) (9)

The evolution of the Si core line spectra with the take-off angle is shown in Figure 3B. To make the effect of the tilt clearer, the spectra are normalized to a common intensity. As can clearly be seen, increasing the tilt angle increases the intensity of the silicon oxide at ~103 eV. Applying Equation (9) it is possible to estimate the thickness of the native oxide, which is ~0.4 nm.

## 2. Quantum Confinement

In the past, a hot area of research from both the experimental and theoretical point of view regarded the study of the electronic structure of small metal clusters. This was motivated by the implications of metal clusters in many areas—in particular, the tremendous technological importance for catalysis. It was observed that the core level binding energies increase with decreasing cluster sizes, and the same occurs for the centroid of the valence band. Conversely, the photoelectron kinetic energy and the valence band splitting decrease [31]. A possible explanation for these effects was discussed in [32]. Essentially, there are two main theories: the first hypothesis explains the photoelectron energy changes as due to variations in the relaxation processes in the final state [33,34]. The second interprets the quantum confinement effects as a size dependence of the initial state [31]. In the first model using a simple approximation, the change in photoelectron energy due to the relaxation processes can be estimated by combining XPS and Auger spectroscopies to calculate the parameter α defined by α = E_k_ + E_B_. The different value of α for a given element in different system configurations is approximately twice the difference in relaxation energies

Δα ~ 2ΔR(10)

The relaxation model satisfactorily accounts for the variations in linewidth, binding energy and Auger kinetic energy due to quantum confinement effects.

The second model explains the changes of the electronic structure of the metal cluster as deriving from a nonintegral d-band configuration and, in particular, its hybridization with empty states above the Fermi level. This hypothesis is supported by experimental data deriving from X-ray absorption and energy-loss spectra and band structure theoretical calculations (see the citations in [31]). Core levels are sensitive to the valence electron configuration. In particular, an energy shift to a lower BE when the d level hybridizes with the s or *p* valence levels leads to an increase in the d-electron number. Both effects of the models modify the electronic structure of the metallic nanoparticles. This was verified in Au clusters, where the intensity of the valence band featured near the Fermi level increased with the increasing cluster size, testifying the increase in the valence electron charge [35,36,37]. A core-level shift of the Au 4f core line was also observed by the same authors [35,36,37]. Because they are size dependent, it is possible to evaluate the nanocluster size from the degree of the photoelectron energy change, as demonstrated in [38]. If we neglect the C-nanostructure characterized by an energy gap, the effects of quantum confinement are expected to also be visible in conducting carbon nanostructures.

## 3. Limits of the Photoelectron Spectroscopies

Current instruments display a typical energy resolution of 0.3 eV. This resolution is generally sufficient to recognize the chemical shifts and assign the different chemical bonds when characterizing the surface of carbon nanostructures. Nevertheless, this energy resolution could be low when quantum confinement effects are studied. The energy shifts induced by the quantum confinement are dependent on the nanostructure dimension: by increasing the structure dimensions, the quantum confinement effects disappear along with the related energy shift. Then, the latter may be just a fraction of the electronvolt well below 0.3 eV, the typical energy resolution of common XPS instruments. Conversely, the energy shift increases by decreasing the nanostructure dimension, making XPS a good complementary technique with increasing sensitivity to estimate the small nanoobject dimensions. Modern instrumentation is also characterized by a high efficiency in collecting photoelectrons, generally obtained using a magnetic lens deflecting the emitted charge into the analyzer. This is used to acquire chemical maps of the material surface. To each pixel of the map, a spectrum mirroring the chemical bonding of the element in that point is associated. However, the maximum lateral resolution amounts to some microns, which is too low compared to objects with dimensions on the nanoscale. When the dimension of the nanostructures is of few nanometers, it is also difficult to distinguish between the surface and bulk or resolve structural conformations. However, as shown in [39], the composition as a function of the depth in structured nanoobjects may be estimated by integrating the contribution of elements as a function of the depth. The sampling depth depends on both the photon energy and the material density. For Al Kα radiation, it varies from ~3 nm for diamonds [40] up to 5 nm in multiwalled carbon nanotubes (MWCNTs) [41]. Then, when dealing with nanodiamonds or few-layer carbon nanotubes, the whole structure is probed, leading to contributions from all parts of the system. A possible solution is to utilize photons with different wavelengths, which may be provided by synchrotron radiation beamlines. In that case, different sampling depths can be obtained, as shown in [42], where the authors studied the surface and bulk fluorination in MWCNTs. Examples of the use of photoelectrons possessing different energies, will be described in the section dedicated to the carbon nanotubes.

As for the surface composition, another important element to consider is the effect of contamination, which, in the case of nanostructures, could be serious due to the extended surface. This problem is more complex when carbon-based objects are considered. A high degree of contamination can derive from CH_x_ hydrocarbons, which can be difficult to be recognized and separated from the nanostructure spectral components. Then, the preparation of the samples and the arrangement on a sample holder are important. An interesting paper containing a rich list of information concerning the use of XPS to characterize nanostructures is given in reference [43].

The principal characteristics of XPS, UPS using He lamp sources or synchrotron radiation and the Hard X-ray Photoelectron Spectroscopy of synchrotrons are summarized in Table 1. 

To analyze the nanostructures, there are other limiting factors to be considered. Nanostructures are commonly deposited on a substrate to be analyzed, and some aspects have to be carefully considered. Attention must be paid to the sample preparation. (i) The possible source of contamination deriving, for example, from the solvent used to suspend the C-nanostructures must be limited. In situ heat treatments to desorb contaminations may be evaluated to purify the sample surface. (ii) The possible contribution to the photoelectron spectra coming from the substrate must be limited. Sufficiently thick films of C-nanostructures have to be deposited, and a substrate with spectral features well-separated from those of C have to be used, although interference cannot be eliminated in valence bands. (iii) The user should select highly flat surfaces to limit the appearance of spectral features from the substrate. (iv) Conducting substrates should be utilized to obtain spectra with reliable binding energy values. However, the possible presence of charging effects can affect the spectra from non-conducting C-NP—for example, nanodiamonds, fullerenes and graphene oxide NPs. In this respect, comparison with the reference spectra may help to obtain optimal charge compensation. This aspect is particularly critical because residual charging may hinder or be erroneously interpreted as an effect generated by the structure nano-size. Important information to correctly analyze C-NPs can be found in [50]. 

## 4. Characterization of Carbon Nanostructures

Due to its versatility, XPS is a very useful analytical tool to control the steps of the synthesis process of materials. The technique may also be applied to study carbon nanostructures, providing the abundance of heteroatoms and their concentration provided it is compatible with the instrument sensitivity. Because XPS probes the material’s electronic structure, it may provide information regarding the carbon atom hybridization, thus adding important elements for understanding the material properties. However, an important note regards the presence of surface contaminants. In this respect, there are two main elements to consider when dealing with XPS. The first is inherent to the technique, which is intrinsically surface-sensitive, as already observed in this manuscript. Surface contaminations could introduce significant errors in the data analysis. The second point regards the nature of the contaminants. They may be composed of foreign species which easily allow for their identification. This is the case for the H_2_O molecules derived from the atmosphere humidity, which are easily adsorbed on the sample surface. Another important source of contamination is the hydrocarbon molecules that are always present on the sample surface. Their contribution cannot be easily separated from that of the carbon nanomaterials, and the only solution is to remove them. Working with nanostructures, the only methods to eliminate the contaminations are the thermal annealing and Ar^+^ sputtering, although the latter may cause non-negligible damage to the carbon nanostructures, introducing a number of defects. Both treatments should be performed under UHV to avoid the exposure to the atmosphere and contaminant molecules. Finally, carbon nanostructures must be deposited on a substrate to place them under UHV and be analyzed. Generally, the deposition occurs via a drying of a colloidal suspension, resulting in a porous structure. The spongy nature of the sample may allow for gaseous species such as CO and CO_2_ to be absorbed. This can contribute to the oxidized components of the carbon peak, introducing errors in the bond quantification. Both surface contamination and absorption should be minimized or accounted for to prevent erroneous conclusions about the chemical state of the sample.

### 4.1. The Analysis of the Electronic Structure of Graphene

There is a general consensus regarding the position of the C 1s derived from pristine graphite. Graphite possesses a semimetallic nature, leading to an unexpectedly high asymmetry. This is the result of the distortion of a pure Lorentzian line shape (apart from Gaussian broadening) induced by energy losses and then located on the high BE side of the peak [51]. The C 1s peak falls at 284.4 eV [15,52,53,54,55,56] for pure highly oriented pyrolytic graphite. The lifetime broadening of the C 1s is around 160–180 meV, and the asymmetry parameter α is in the range 0.05–0.19 [56,57,58]. 

Graphene is a carbon nanostructure strictly connected with graphite. Graphene is, in fact, the single graphitic layer of carbon atoms and a stack of graphene layers correctly overlapping each other, which leads to the formation of graphite. Carbon atoms in graphene are sp^2^ hybridized and connected with three neighboring carbon atoms via σ bonds, while the remaining unhybridized z orbital is aligned along the vertical axis graphene sheet, forming π bonds [59]. The σ bond in graphene is about 1.42 Å long, resulting in a strength higher than that of diamonds (42 N/m and Young’s modulus of 1.0 TPa) [60]. The conjugated out-of-planar π bonds allow for electron mobility as high as 200,000 cm^2^/V·s in suspended graphene and prominent electrical conductivity (6300 S/cm) [61]. Graphene also possesses a high thermal conductivity of about 5000 WmK^−1^. Finally, graphene is characterized by a high specific surface area of 2630 m^2^/g [62] and a transparency of 97.7% on the whole visible range, resulting in a very high absorbing power of 2.3% for a single atomic layer [63]. 

Graphene can be produced by bottom-up methods or via a top down exfoliation of graphite mechanical routes. In the first case, high quality graphene layers may be obtained via an epitaxial growth under ultra-high vacuum based on the thermal decomposition of SiC [64]. However, the drawback of this technique is the lack of scalability of the method, resulting in the production limited to experimental needs. A solution to this problem is the use of CVD processes, which permit the production of large area graphene sheets. In CVD processes, graphene is produced by the high temperature catalytical decomposition of a hydrocarbon gaseous precursor [65]. Graphene can also be produced through top-down methods. Among these, mechanical exfoliation by using adhesive tape was the first documented method to separate the single graphene layer from crystalline graphite [66]. A high quantity of graphene may be produced via the liquid exfoliation of graphite, N-methylpyrrolidone [67] or by high-energy ball milling, which can be performed in both wet [68] and dry conditions [69]. The chemical route uses strong acids to exfoliate graphite [70] and is broadly applied because of the high process yield and scalability. This method, however, produces highly oxidized graphene sheets. Reduced graphene oxide is then applied to remove oxygen from the graphene oxide sheets. Chemical reductants [71] or thermal treatments [72] may be used to perform the reduction. Thanks to the scalability of these processes and their availability on the market, graphene and its derivatives have gained increasing interest for energy applications [63] in electronics, photonics [73], catalysis [74] and sensing [75] and biosensing applications [76].

**Figure 4:**
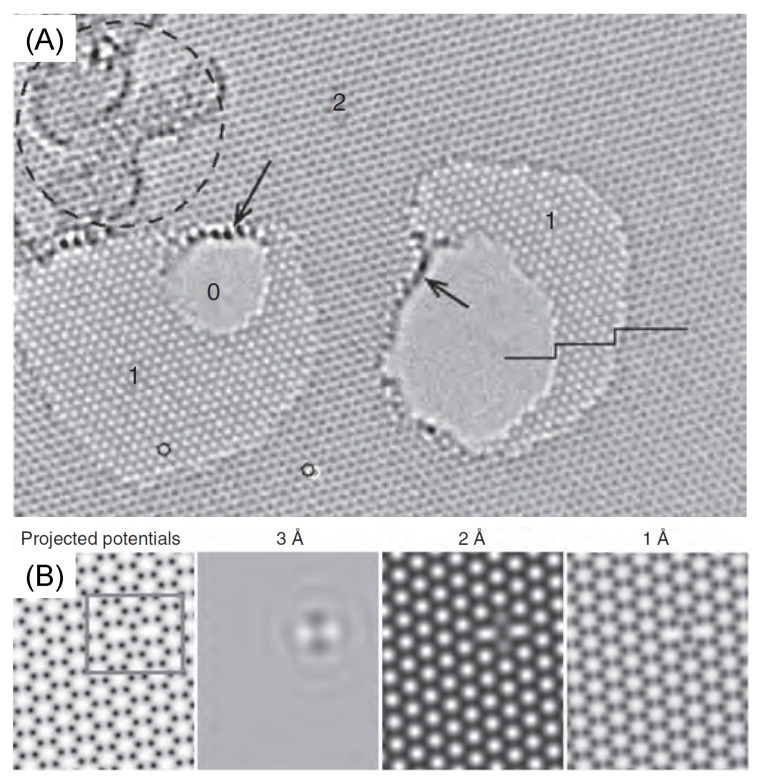
(**A**) HRTEM image example of graphene, showing a hole, a monolayer and bilayer areas. Contamination and small amorphous contamination are indicated by arrows. The dashed circle indicates a region of highly defected graphene with amorphous patches. (**B**) The graphene defect HRTEM shows a pentagon–heptagon configuration at different resolutions. Reprinted with permission from [77].

The quality of the graphene is essential for the sensitivity and efficiency of the sensing device. High-resolution transmission electron microscopy (HRTEM) may be utilized to determine the presence of defects and impurities [77]. An example is given in Figure 4. In (A), an aberration-corrected HRTEM image in which the bilayer, monolayer graphene and holes are identified by 2, 1 and 0 is shown. The arrows indicate contaminations, while the dashed line delimits a highly defected region. The very high resolution of HRTEM allows for the identification of defects at the atomic level. Figure 4B shows how the graphene pentagon–heptagon configurations appear at different resolutions.

**Figure 5:**
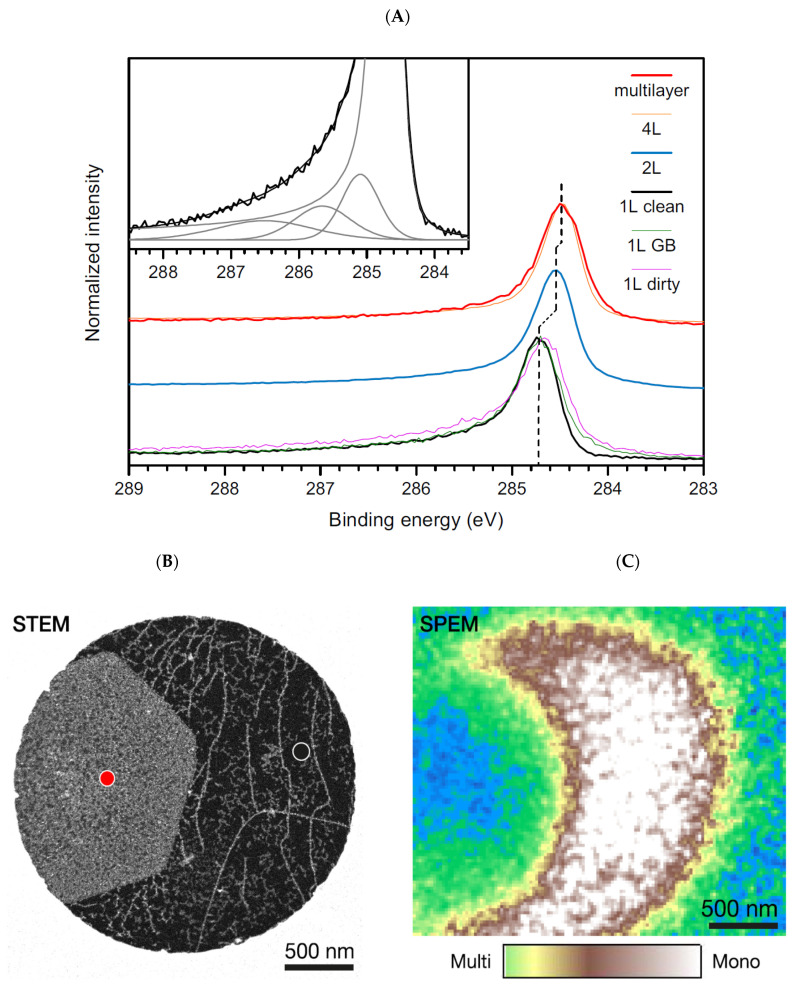
(**A**) C 1s XPS spectra collected from 130 nm diameter spots characterized by STEM, with the lines colored according to the color of each spot marked on the STEM images: dirty monolayer (1L) and 1L with a grain boundary (GB), clean 1L and multilayer and two-layer (2L) and four-layer (4L) graphene. The inset provides a deconvolution of the 1L spectrum, which contains small residual contributions from nongraphitic carbon. (**B**) Monolayer graphene (black spot) with an overlying multilayer grain (red spot) measured using STEM. (**C**) SPEM colored according to the ratio of the signal in energy windows corresponding to mono- (284.57, 284.98) eV and multilayer (283.94, 284.49) eV graphene. Reproduced with permission from [78].

Concerning characterization, the graphene C 1s core line would be the one derived from a free-standing single layer. However, despite how easily it can be imagined, it is very complex to perform XPS analysis on a free-standing single layer because it is not possible to produce enough extended suspended graphene sheets allowing for the analysis to be performed with conventional instruments. Graphene is characterized by a single atomic layer with the consequent possible generation of quantum confinement effects and the consequent influence on the core-hole screening. In particular, a less screened photohole causes a more intense coulombic field, leading to an increased BE. It is then expected that the C 1s of the single graphene sheet is shifted to higher binding energies with respect to those of graphite. It is also important to observe that, contrary to graphite, where a bulk and a surface component are visible in the C 1s core line spectrum [53,56], in graphene, the bulk component is obviously absent. Synchrotron radiation was utilized together with a scanning photoelectron microscopy facility to analyze a 3 × 3 μm area of a suspended graphene sheet [78]. The authors were able to discriminate among multilayer structures and two-layer and single-layer graphene portions. In Figure 5A, the C 1s of a multilayer structure resembles that of graphite, as expected. The presence of quantum confinement disappears, and the C 1s peak falls at 284.46 eV. Reducing the system at two layers, there is a small confinement effect, leading the carbon peak to fall at a slightly higher BE. For the single graphene layer shown in Figure 5B, the C 1s photoelectron spectrum is peaked at a BE of 284.7 eV. The difference in binding energy was utilized in scanning photoelectron microscopy (SPEM) to acquire maps capable of discriminating between mono- and multilayered regions Figure 5C. 

These results are in agreement with the evidence obtained from epitaxial graphene grown on SiC, where a linear trend of the BE shift as a function of the number of layers is found. The position of the C 1s in single-layer graphene falls at 284.8 eV [79]. In another work, the authors studied the evolution of the C 1s core line of a SiC sample at different annealing temperatures. The peak is formed by three components: at a lower BE at ~283 eV, carbon falls in SiC. At 284.7 eV, the authors found the carbon from graphene, while a higher BE of 285.85 eV was assigned to the buffer layer used [80]. The authors found that, by increasing the temperature from 1125 °C to 1375 °C, the intensity of the component relative to the graphene increases, as expected because of the thermal decomposition of SiC and the gradual formation of the graphene layer.

However, some effects deriving from the substrate have to be expected in both these works. The interaction of graphene with SiC is very weak. However, the difference in the work functions of the two systems may lead to a small BE shift of the C 1s peak, which is quantified in ~0.2 eV [81]. The shift of the graphene work function due to the interaction with the substrate also leads to a change in the position of the Dirac point. However, the authors of [78] measured a null shift of the Dirac point, thus ensuring a null influence of the environment on the position of the C 1s peak. The observed shift of their carbon peak with respect to that of the pristine crystalline graphite is then derived from a reduced screening of the photohole induced by quantum confinement effects.

The graphene on Ir(111) was characterized by angle-resolved photoelectron spectroscopy (ARPES) to understand the dependence of the electronic structure on the preparation process [82]. The authors probed the Dirac cone band structure using synchrotron radiation and found that the interaction of graphene with the substrate led to 0.1 eV hole doping and the formation of replica bands and of a minigap at the Dirac point. The results show that the drop in intensity of the ARPES intensity of the Ir(111) surface states can be explained only by a specific interaction of the Ir substrate with the graphene layer. Because of the faster reduction in the intensity compared with the graphene growth on the Ir substrate, an additional quenching mechanism generated by the scattering induced by strongly bound graphene edges is also hypothesized.

The author of this manuscript characterized monolayer graphene deposited on Cu via a supersonic beam of fullerenes and separated the contribution from the substrate via ARXPS and a conventional XPS instrument [83].

**Figure 6:**
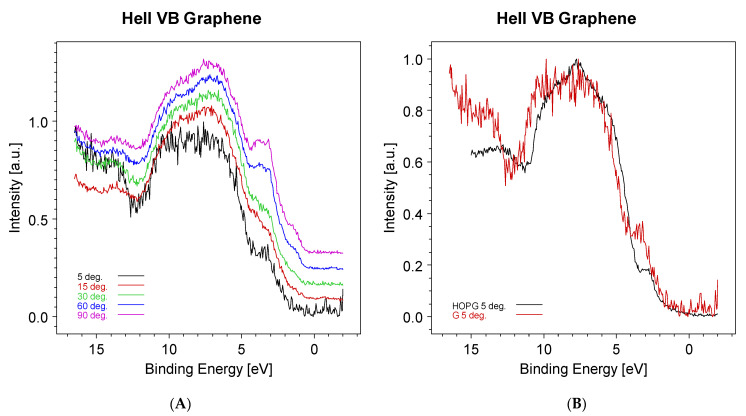
(**A**) evolution of the graphene HeII valence band as a function of the tilt angle. (**B**) Comparison of the He II valence band from highly oriented pyrolytic graphite and of the graphene at a 5° take-off angle. Figures produced by the manuscript author.

In Figure 6, the evolution of the graphene valence band obtained using a HeII (40.8 eV) photon source is shown. The prominent peak at ~3.5 eV is due to the copper 3d contribution. In an attempt to reduce the intensity of this band, the take-off angle was reduced from 90° down to 5°. At this sample inclination, the sampling depth is expected to be reduced at fractions of nanometers and then of the order of the graphene thickness.

As can be seen in Figure 6A, the Cu 3d band intensity decreases with the decrease in the take-off angle. It is also possible to follow the evolution of the Cu Fermi level, which disappears at a 5° inclination. In Figure 6B, the HeII valence band from the graphene (red) is compared to that from the highly oriented pyrolytic graphite (black). The valence band shift to higher binding energies could be explained as an effect of the quantum confinement of electrons in the single graphene sheet.

The transparency of graphene to photoelectrons was utilized to estimate the number of layers of suspended membranes [84]. An electron mean free path was modeled using a TPP-2M model, and the corresponding electron attenuation length was compared with experimental data obtained from the photoemission spectra. The deviation of the TPP-2M model at low kinetic energies is likely to be caused by scattering processes. The authors found that, for a single or very low number of layers, the graphene membranes are truly electron-transparent, even at low electron kinetic energies, allowing for a correct measure of the number of graphene layers. The differences in the inelastic mean free path of electrons was utilized by other authors to measure the thickness of graphene sheets through Auger electron spectroscopy [85]. The results show that the C KLL Auger spectra change in intensity and spectra line shape by varying the number of graphene layers. The authors were also able to calculate the inelastic mean free path as a function of the electron energy. The C KLL Auger spectra display distinct fingerprints, with a decrease in the number of graphene layers, enabling the estimation of their number despite the presence of strong electronic coupling with an underlying substrate. 

**Figure 7:**
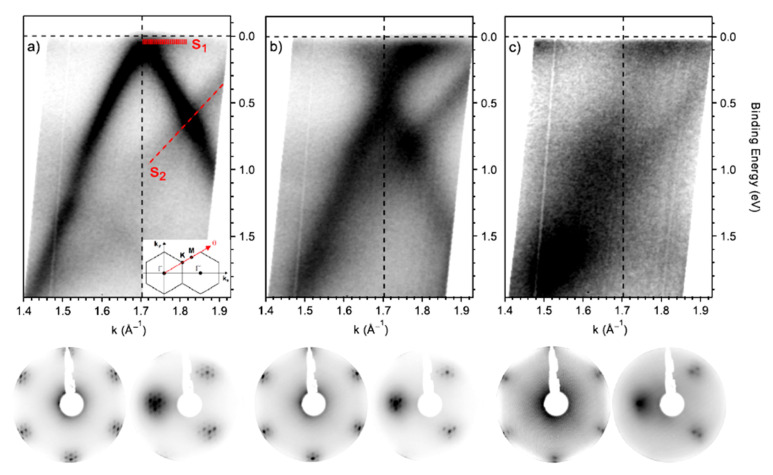
Top panels: band dispersion at the K point of the Brillouin zone obtained by plotting the ARPES intensity as a function of the wave vector and BE for (**a**) pristine graphene/Ir(111), (**b**) after nitrogen doping and annealing at 600 °C and (**c**) after oxygen exposure. The red dashed lines indicate the two Ir surface states, S1 and S2. Inset: experimental geometry of the ARPES experiment. Bottom panels: the corresponding LEED patterns respectively recorded with an electron energy of 78 eV with the (0,0) spot at the center of the image and at an off-angle of 10°. Reprinted with permission from [101].

XPS is the technique of choice to analyze the material surface chemistry. Descriptions of the surface composition and elemental quantification are commonly carried out to characterize the graphene and graphene derivatives and the results of surface functionalization. This process is commonly applied to optimize the graphene properties for selected applications. There are a plethora of articles in the literature describing the different functional groups grafted on the graphene surface and the surface molecular engineering needed for material, sensing, imaging, biomedicine, energy and other applications [86,87,88,89,90]. We allow the reader to explore the wide literature while giving some information regarding the modification of the electronic properties of graphene via functionalizing its surface and how it is studied. In chemical functionalization, the interaction with exogenous molecules considerably changes the graphene electronic properties [91] in terms of carrier concentration, their scattering, the related transport mechanisms, the polarity, the quantum-capacitance, the energy levels and the orbital hybridization. It is then important to be able to puzzle out the effects of the functionalization induced by the molecular interactions. There are a few mechanisms that directly modify the graphene electrical properties: functionalization induces a change in the carbon chemical potential [91] and the energy levels of the hybridization of the carbon atoms [92], and the consequent structural conformation induces the formation of dipoles and changes in the density-of-states (DOS) [93,94]. A broadly utilized method to produce single-layer graphene is the Hummer exfoliation of graphite, leading to oxidized graphene single layers with the formation of carboxyl, epoxy, hydroxyl, carbonyl, phenol, lactone and quinine functionalities [95]. The density of abundant carbon–oxygen σ-bonds with more localized electrons disorganizes the π cloud and converts the graphene into an insulator. Change in the carbon atom hybridization occurs also with the N doping of graphene. N-doped graphene exhibit n-type conductivity, although the introduction of defects in the π delocalized quasi-free electrons reduces the carrier mobility by ~30% [96]. The π-π interaction of graphene with aromatic molecules does not introduce structural distortions but may introduce dipoles, thus enhancing the local effective electric fields. Additionally, the graphene DOS is changed by changing the charge density [96]. More information may be found in [96]. Concerning characterization, the presence of functionalities is revealed by analyzing the C 1s core line. An example is given in [97]. The effect of doping on the electronic structure of graphene is provided in [98]. The Raman spectra of P- and N-doped graphene display an up-shift of the G peak, mirroring the occurrence of doping [99]. The inspection of the Raman spectrum of N-doped graphene shows that the 2D band is shifted to higher frequencies occurring in presence of hole doping, while lower frequencies are found in the presence of electron doping [99,100]. Additionally, the FWHM of the G band is sensitive to doping [100]. Then, Raman spectra may used to confirm the doping in the presence of N and P heteroatoms detected by XPS. The valence band of N-doped graphene shows a significantly increased DOS near the Fermi level that is consistent with the increased number of charge carriers, while pristine graphene and P-doped graphene display a similar valence band [98]. In another study, the authors report the interaction of molecular oxygen with N-doped graphene [101]. Graphene was grown on an Ir substrate to obtain large crystals. XPS core level spectra were extensively analyzed to unravel the presence of N-doping and the dissociation of O_2_ molecules by the doped graphene, excluding the possible contribution of the Ir substrate. Then, the sample was analyzed by angle-resolved XPS, and the diffraction patterns obtained by the sum of the spectra in the p and s polarizations are shown in Figure 7.

Figure 7a shows the well-known π band (Dirac cone) along with the Γ–K–M of pristine graphene, with the Dirac point slightly above the Fermi level, indicating the presence of a small p-doping. S1 and S2 indicate the surface state of the iridium substrate. Nitrogen doping leads to a sensible downshift in the Dirac point, now located at 0.44 ± 0.02 eV below the Fermi level, reflecting the presence of significant n-doping due to nitrogen (Figure 7b). The doping does not affect the linear trend of the π band, and the S1 and S2 surface states of iridium are still visible. The doping induces an increase of 0.105 Å^−1^ of the FWHM of the π band due to the higher scattering caused by the nitrogen defects. The exposure to oxygen molecules induces both a broadening and a flattening of the π band, as shown in Figure 7c. These changes are caused by the formation of C− bonds with the consequent change of the C hybridization from sp^2^ to sp^3^. This loss is also visible in the LEED pattern with a loss of definition caused by a strong increase in the background, which almost hides the Moiré superstructure. Finally, oxygen induces the generation of a bandgap of about 0.3 eV at the K point. 

### 4.2. Characterization of Carbon Nanotubes

Carbon nanotubes (CNT) are very peculiar nanostructures showing low dimensionality in their section, while they can be considered mesoscopic systems because they can possess macroscopic dimensions along their principal axis. For this reason, they are fascinating objects possessing a unique band structure derived from the two-dimensional confinement, leading to special transport properties. In the high temperature range, where it is possible to use semiclassical models, the transport in CNTs does not reflect those of their parent 3D carbons and graphites. The low dimensionality causes weak localization effects, low Coulomb interactions and universal conductance fluctuations in the quasi-ballistic regime. To describe the transport mechanisms in the CNTs, the electronic structure and the effect of the single- or multi-walls, chirality, defects and doping have to be described.

**Figure 8:**
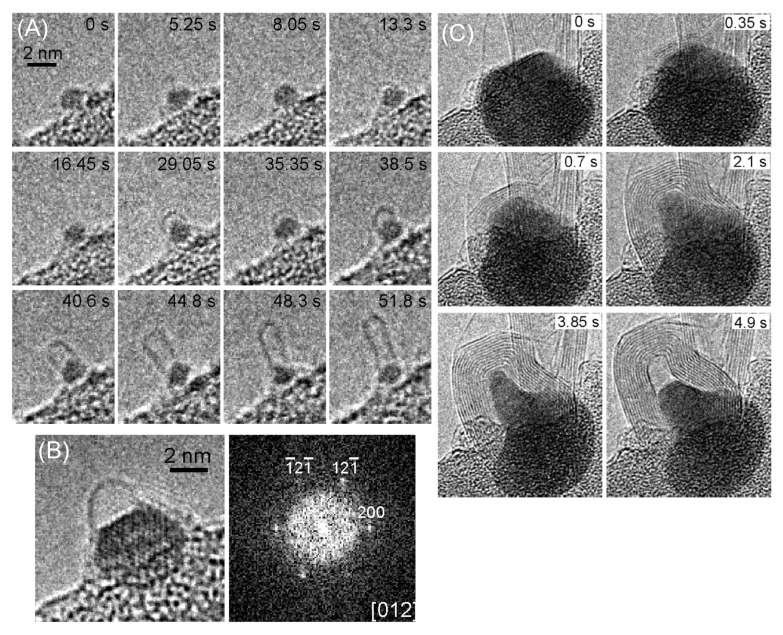
Nucleation and growth process of a SWNT from a nanoparticle Fe catalyst on a substrate. (**A**) Structural fluctuation of both carbon caps and a nanoparticle catalyst (NPC) is observed. The recording time is shown in the images. (**B**) A snapshot of an NPC with a carbon dome. The NPC exhibits the lattice image and the corresponding extra diffraction in the Fourier transform. The NPC can be identified as Fe-carbide (cementite, Fe3C) viewed along the [012] direction. (**C**) Nucleation and growth of a MWNT from an NPC on a substrate. Graphene layers are formed on an NPC, and then a MWNT is suddenly expelled from the deformed NPC. The recording time is shown in the images. Reprinted with permission from [110].

Photoelectron spectroscopy is one of the more appropriate techniques to probe the electronic properties of CNTs. The latter may be categorized in single-walled CNTs (SWCNT) and multiwalled CNTs (MWCNT). In addition, in SWCNTs, the chirality is used to discriminate between metallic and semiconducting nanotubes [102]. Various synthesis processes are utilized to produce these different kinds of CNTs. A very popular method is the arc discharge ignited in the presence of a catalyst placed in the discharge electrodes. The process conduces to the production of both SWCNTs and MWCNTs [103,104]. Process parameters such as the kind of atmosphere, pressure and arc current strongly influence the quality of the CNTs [105]. SWCNTs may be produced by arc discharge in an H/Ar atmosphere in the presence of catalysts such as Ni, Fe, Co, Pd, Ag, Pt, etc. or mixtures of Co, Fe and Ni with other elements such as Co–Ni, Fe–Ni, Fe–Co, Co–Cu, Ni–Cu and Ni–Ti [106]. The production yield depends on the catalyst selection. CNTs can be synthesized also by laser ablation [107], but the process yield is limited. Chemical vapor deposition (CVD) is broadly utilized to produce higher amounts of CNTs. Hydrocarbon precursors such as methane, acetylene, ethane, ethylene or alcohols are decomposed in the presence of catalysts such as Ni, Co and Fe at high temperatures [108]. The mass production of CNTs is obtained using the controlled combustion of fuels such as methane, ethanol, ethylene, methylene acetylene and propane in the presence of catalysts [109]. Figure 8 shows the progression of SWCNT and MWCNT growth with time [110]. In Figure 8A, the nucleation of unstable carbon cages first appears. After an incubation period of 29.5 s, a stable SWCNT dome is formed. Then, a SWCNT with a 1.5 nm diameter grows, and its length increases with time. Figure 8B shows the generation of a single wall dome on a Fe crystalline nanoparticle (NP). Figure 7C shows the growth of a MWCNT: first, a graphene layer is synthesized on the catalyzer surface. Then, individual graphene layers grow and extend, resulting in MWCNT formation. During the MWCNT synthesis, the catalyzer NP deforms, forming the characteristic protrusion and expelling the MWCNT. A high degree of CNT purity may be an essential requirement for applications in biomedicine and electronics needing a complete elimination of the metallic catalysts and other toxic elements [111,112]. 

XPS is commonly utilized to detect the CNT composition and the degree of contamination as well as the quality and electronic properties of the CNTs and how they change upon functionalization. In [113], purified MWCNTs were analyzed using photoelectron spectroscopies and other techniques to describe their optical properties. The MWCNTs’ diameter was in the range of 15–20 nm. In particular, the UPS HeII valence band (VB) appears to be very similar to that of graphite, although the intensity of the spectral features below 8 eV is lower for the MWCNTs. According to theoretical models [114], this region is associated with 2p-π and with the top of 2p-σ. In CNT, the reduced intensity of these two bands is explained as a result of the curvature of the nanotubes causing a lowering of the p-π electron density. The opposite trend is found in the VB region around 11.5 eV. At this energy, the p-σ band falls and the increased spectral intensity of the CNTs is interpreted as an effect of the σ-π hybridization. Depending on the diameter, CNTs exhibit a variable energy gap [115] which decreases with the increase in the diameter size. This was experimentally observed in [116] in both semiconducting and metallic SWCNTs. The authors used Raman spectroscopy to evaluate the semiconducting or metallic character of the SWCNTs and the diameter size. The latter was correlated with the value of the energy gap obtained by the combined use of HeII valence spectra to probe the density of the states below the Fermi edge and inverse photoemission spectroscopy to probe the unoccupied states above the Fermi. Interestingly, using these two techniques, the authors were able to reproduce the DOS. HeI radiation was utilized to evaluate the work function of MWCNTs [113]. The authors found that the secondary electron cutoff relative to the MWCNTs is shifted by 0.2 eV to a higher BE with respect to that of graphite. This means that MWCNTs exhibit a 0.2 eV lower work function with respect to graphite. An opposite result was obtained for SWCNTs [117]. In this case, the authors found that SWCNTs possess a work function of 4.8 eV, while the value of graphite was 4.6 eV. This can be explained as an effect of the quantum confinement, which is absent in MWCNTs, systems where the quantum size effects are progressively lost because of the tendency to resemble the graphitic system structure. 

Let us turn our attention to the C 1s core level. Despite being the higher value of the work function, the position of the C 1s peak—which, in graphite, falls at 284.6 eV—is shifted in MWCNTs of ~0.3 eV to a lower BE [113,117]. This lower value is ascribed to the CNT curvature, which weakens the C−C bond. In addition, the asymmetry parameter of the Doniac–Sunjic function used for the peak fitting is higher for MWCNTs than that of graphite, revealing the “more metallic” character of the MWCNTs. A high-resolution C 1s spectrum of suspended SWCNTs was acquired using a synchrotron radiation microprobe with a spatial resolution of 100 nm. The C 1s falls at a BE of ~284.6 eV—compatible with that of highly oriented pyrolytic graphite (HOPG) and higher than that of MWCNTs. The shift to a higher BE can be explained as an effect of quantum confinement. Regarding the line shape, also in this case, the C 1s appears to be asymmetric, with a Lorentzian core-hole lifetime broadening of 0.22 eV and an asymmetry parameter of 0.19. These values are approximately twice those of HOPG [52], indicating a higher DOS near the Fermi level. Finally, the C 1s in CNTs of different diameters always displays a full width at a half maximum (FWHM) higher than that of graphite, revealing a shorter lifetime of the holes in comparison to that of graphite [118].

**Figure 9:**
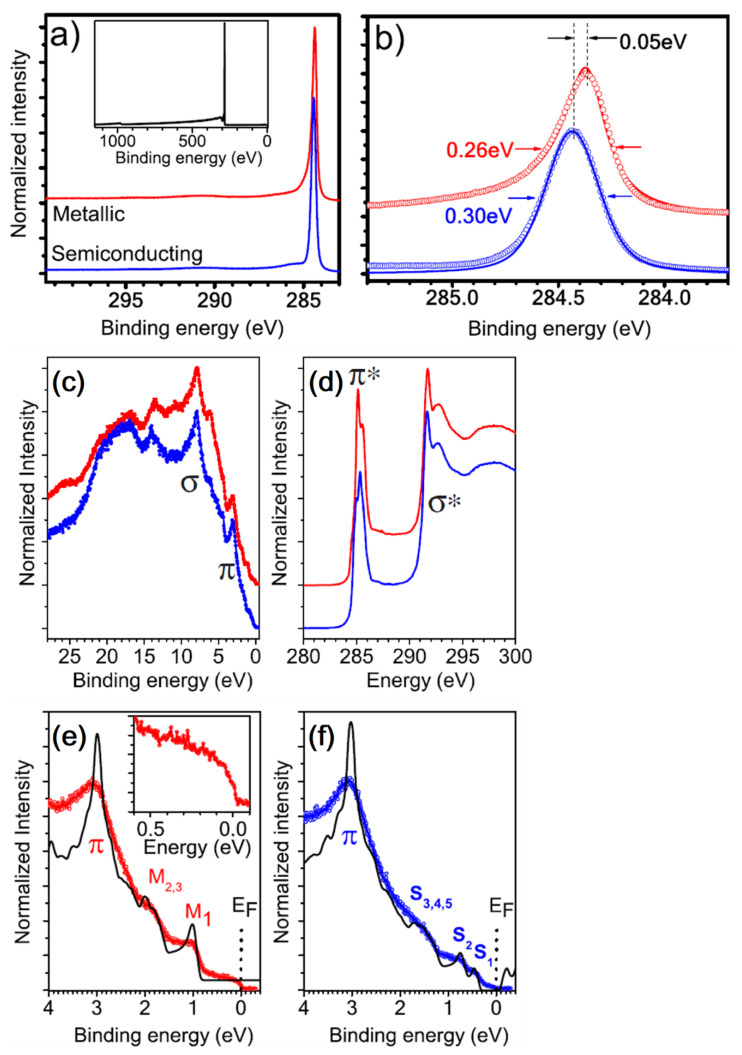
(**a**) C1s photoemission of metallic SWCNT (upper curve) and semiconducting SWCNT (lower curve) as conducted at 400 eV photon energy. The inset shows a PES survey scan up to 1200 eV. (**b**) The C1s line of the metallic and semiconducting SWCNT on an expanded scale. The solid lines are fits using a line shape analysis with a Doniach-Sunjic (metal) and Voigtian line shapes (semiconductors). (**c**) Valence band photoemission spectra of metallic (upper curve) and semiconducting (lower curve) conducted at 150 eV photon energy. (**d**) High-resolution XAS response of metallic (upper curve) and semiconducting (lower blue curve) showing the π* resonance at 285.4 eV and the σ* threshold at 291.7 eV. (**e**) π band response in the valence band PES conducted at 150 eV for the metallic and the semiconducting SWCNTs. (**f**) The inset in a shows the expanded region around E_F_ on an expanded scale. The black lines in the main panels show the fraction of the tight binding DOS of the semiconducting and metallic SWCNTs probed. The labels M_1…3_ and S_1…5_ depict the position of the van Hove singularities. Reproduced with permission from [122].

In [119], authors analyzed the C 1s derived from SWCNTs produced by different methods, namely, arc discharge, high-pressure CO conversion (HiPCO), laser ablation and CoMo catalysts (CoMoCat). The different synthesis methods result in different FWHMs of 0.91, 0.86, 0.81 and 0.88 eV for the arc discharge, HiPCO, laser ablation and CoMoCat methods, respectively. The FWHM is also affected by the CNT diameter size, the different metallic character affecting the Lorentzian lifetime associated with the core line, and by oxidation [119]. The presence of oxygen or the semiconducting character of the CNT may cause mild effects of charging. Evidence of this effect can be seen by looking at the Fermi energy region and, in particular, to the distortion of the DOS [120]. Concerning the loss structures, similar to what occurs for the HOPG, the CNT displays a feature at a BE 5.9 eV above the C 1s peak. This loss shakeup component derives from the collective excitation of the π- electrons due to the π → π* transitions during the photoemission. The definition of this structure is dependent on the SWCNT synthesis method and appears sharper in case the SWCNTs are produced by laser ablation, while it appears more or less embedded in the C 1s tail in the other cases (arc, HiPCO, CoMoCat). An additional broad feature is also seen at ~27 eV and can be attributed to collective σ + π plasma. Because of the curvature, π electrons are not uniformly distributed with respect the nanotube surface, like what happens for the flat graphite sheets. This induces asymmetries in the excitation of the σ + π electrons. In general, for carbon nanostructures, the intensity of the loss features depends on the density of the σ and π states placed not too far from the Fermi level [121]. In MWCNTs, the plasmon resonance feature is shifted to higher BE values [113] due to the redistribution of the electronic charge of the π electrons on the external side of the rolled graphene sheets. Interestingly, the C 1s may be utilized to discriminate between metallic and semiconducting CNTs [122]. Purified metallic and semiconducting SWCNTs with a Gaussian diameter distribution with average size of 1.37 nm were deposited on a sapphire substrate to produce thin films. Figure 9a shows the C 1s derived from metallic and semiconducting CNTs. The high-resolution spectrum depicted in Figure 9b clearly shows the different metallic/semiconducting character of the CNTs: the former is described by an asymmetric Doniac–Sunijic line shape (α = 0.11), while the second displays a nearly symmetric Voigt C 1s profile. Figure 8b also shows the position of the C 1s peak falling at 284.48 eV and 284.43 eV for metallic and semiconducting CNTs, respectively, and their FWHM of 0.26 eV and 0.30 eV, respectively, is surprisingly narrow compared to that of graphite (0.32 eV). Differences are also observed in the valence bands and in the X-ray absorption spectra (XAS), shown respectively in Figure 9c,d. The XAS spectra display two peaks representing the π* and σ* falling at 285.4 eV and 291.7 eV, respectively, which evokes that of graphite [123]. Differences in the π* fine structure can be related to the distinct van Hove singularities in the unoccupied DOS of the SWCNTs’ [124] clear signature of metallic/semiconducting character. In parallel to the analysis of the unoccupied DOS, Figure 9c displays the SWCNT DOS. Both metallic and semiconducting CNTs exhibit a π band at ~3 eV, in agreement with theoretical calculations [125].

Figure 9e,f display the CNT DOS in the region near the Fermi edge E_F_. In particular, the van Hove singularities M and S are also indicated. The metallic CNTs display only M singularities, with the M_1_ falling at −1.05 eV and the M_2,3_ falling at 1.8 and 2 eV. Conversely, the semiconducting CNTs spectrum shows the S_1_ and S_2_ peaks at 0.44 and 0.75 eV, respectively. The fine structure of the DOS is sensitive to the chirality of the CNTs.

**Figure 10:**
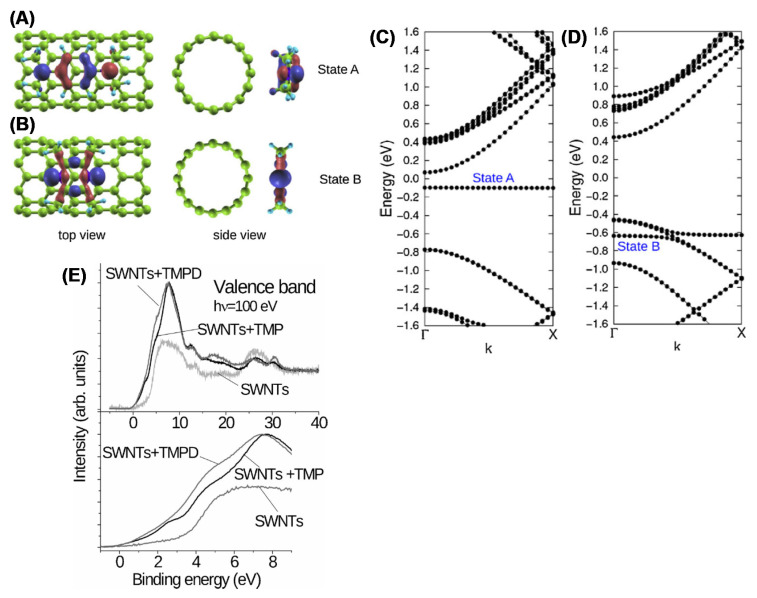
Wave functions of the localized states in the band structures corresponding to the dopant molecules (**A**) TMPD and (**B**) TMP on the surface of the (8,0) carbon nanotube; (**C**,**D**) band structures of the (8,0) CNT interacting with the TMPD and TMP, respectively. The Fermi level is set to zero. (**E**) The XPS valence band spectra of the pristine SWNT networks and the SWNT networks modified by aromatic amines. Reproduced with permission from [134].

XPS is also extensively utilized to analyze the chemical composition of the CNTs after functionalization [126] and the consequent change of the electronic properties. Oxygen-, nitrogen- and fluorine-functionalized CNTs are carefully characterized in [127], providing a detailed description of the possible chemical bonds and the relative BEs [128] (see also references therein). The presence of functionalities on the CNT surface leads to the reduction of the of van der Waals interactions between nanotubes, which highly facilitates the separation of nanotube bundles. If the functional groups possess a polarity, then the CNTs become water soluble. The different methods to functionalize the CNT are reviewed in [87,129]. The grafting of functional groups such as COOH, CH_3_, NH_2_, H, OH, F, etc. introduces defects in the CNT structure by changing the carbon atom hybridization from sp^2^ to sp^3^. This results in the creation of an impurity state around the Fermi level [130]. Functionalization is then expected to change the electronic DOS near the Fermi edge. The effect of the oxidation induced by ball milling on the surface of the SWCNT was studied in [131]. The authors show an increased energy gap in functionalized CNTs due to the charge transfer from C to the oxygen-based functional groups and a decrease in the VB intensity i.e., the density of states, at the top of the VB. The same authors estimated the change in the cutoff energy of photoelectrons in HeI-excited VB. They found that the cutoff energy shifted by 1.6 eV in oxygen-functionalized SWCNTs, leading to a corresponding reduction in the work function. The reduction in the work function may be explained through the formation of a dipole formed by a negative OH^−^ and a positive C atom. Finally, the authors found that the impurity state is distributed not only around the –OH group but over the entire unit cell. Similar results are obtained in fluorinated SWCNTs. With increasing F-doping content, XPS shows a parallel reduction in the DOS near the Fermi level [132]. This results in a transition from the metallic to the semiconducting character of the CNTs. The N doping of CNTs induces a remarkable change in the electronic properties, which are studied in detail in [133]. Nitrogen and nitrogen-based molecules are utilized to modify the electronic structure of the SWCNTs. As an example, in [134], aromatic amines were utilized to functionalize SWCNTs. The modification of the density of state induced by two different molecules namely, N,N,N0N0-tetramethyl-p-phenylenediamine (TMPD) and tetramethylpyrazine (TMP) — obtained via ab initio density functional calculations is shown in Figure 10A,B, together with the charge contour plot of the TMPD and TMP attached to the SWCNTs. Figure 10A,B show that functionalization with the TMPD and TMP leads to the formation of the two flat bands (states A and B, respectively). The highest occupied orbitals of the TMPD aromatic molecule exhibit a π-bonding character, whereas that of the TMP possesses a σ-bonding character. In the case of TMPD molecules, a charge transfer occurs, with an increased electron density on the CNTs, thus inducing n-type doping. On the contrary, the band structure of the TMP leads the formation of a TMP-derived localized state (state B) at about 0.6 eV below the Fermi level. This demonstrates that the molecular orbitals of the functional groups play a crucial role in controlling the doping level of the semiconducting CNT. 

The possibility to probe the CNT functionalization as a function of the depth is interesting. In [135], the authors compared the C 1s and C KLL Auger spectra from fluorinated MWCNTs, SWCNTs, carbon fibers and HOPGs. The different kinetic energies of the C 1s photoelectrons of the C 1s (~1202 eV) and of the C KLL Auger features (~260 eV) allow for probing ~10 layers or only 2 layers, respectively [41]. The experiments show that the carbon structures present a similar composition characterized by CF, CF_2_ and CF_3_ and CF_4_ bonds. However, the analysis of the CKVV spectra enlightened the absence of C-F bonding in the outer layers of the fluorinated MWCNTs, Fibers and HOPGs, while it exists in the outer layer of the SWCNT. Photons with different energies were also utilized to study the electronic structure of CN upon fluorination [42]. The authors found that the fluorination proceeds uniformly without dependence on the fluorine concentration. As expected, fluorine induced a change in the carbon atom hybridization from trigonal sp^2^ to tetrahedral sp^3^, with a consistent charge transfer between the carbon and fluorine atoms. Interestingly, by decreasing the photon energy from 1130 to 345 eV (the sampling depth varies from 2 nm to 0.4 nm), the intensity of the structure assigned to the CF bonds weakens.

### 4.3. Quantum Confinement Effects in Fullerenes

Fullerenes represent one of the more peculiar allotropic forms of carbon, where atoms are arranged in cage-like stable structures. This possibility was theoretically foreseen in the year 1970 by Hückel calculations predicting the possibility to arrange 60 carbon atoms in a superaromatic π-system [136]. The experimental evidence of this possibility was obtained only in 1985, where a mass spectrometer detected a dominant peak at *m*/*z* 720 corresponding to the C_60_ molecule. A second peak at *m*/*z* 820 was assigned to C_70_. Fullerenes are very stable and strong structures, allowing for sustaining pressures higher than 3000 atm without ruptures [137]. The more popular methods to synthesize fullerenes are the vaporization of graphite electrodes using arc or plasma discharges [138,139], the pyrolysis of hydrocarbons [140,141] and laser ablation [138,142]. Fullerenes are separated from soot using solvents such as benzene or toluene, while fullerenes with different sizes are separated using column or liquid chromatography [143]. As carbon nanotubes can be thought of as graphene layers rolled along a principal axis, the fullerene can be conceived as a single layer of carbon atoms folded to form a sphere. However, for geometrical reasons, this can be accomplished only by alternating hexagons and pentagons. The combination of hexagons and pentagons to generate a sphere is possible only with a “magic” number of carbon atoms: fullerenes are C_2n_ molecules with *n* = 10, 12, 13, 14, etc. Surprisingly, the C_22_ does not exist in nature, while the more frequent forms are C_60_ and C_70_. The presence of pentagon has important consequences on the electronic properties of the fullerenes. In pentagons, any bond resonance is suppressed, leading to fullerene molecules with poor electron delocalization. Contrary to what was predicted from the theory, fullerenes are not superaromatic structures. The study of the electron distribution in fullerenes is therefore essential to understand the reactivity of fullerenes towards other chemical species. In this respect, XPS plays a fundamental role in characterizing the fullerene properties and designing new fullerene-based materials. We will consider C_60_ as the prototype of the fullerene family. C_60_ possesses a triply degenerate low-lying lowest unoccupied molecular orbital (LUMO). This makes this molecule a very efficient electron acceptor, accommodating up to six electrons and thus facilitating the formation of donor-acceptor conjugates [144]. Furthermore, the special structure of fullerenes with high surface convexities containing a number of highly reactive double bonds imparts a pronounced chemical reactivity [145,146]. Fullerenes also display non-conventional properties such as superconductivity when coupled to alkali metals [147,148], ferromagnetic properties [149], non linear optical properties [150] and electrical and photocatalytic properties [144,151]. The difference in the aromatic character of a pure sp^2^ system as the highly oriented pyrolytic graphite sheet, and the C_60_-fullerene appears from the energy position of the C 1s core line. 

**Figure 11:**
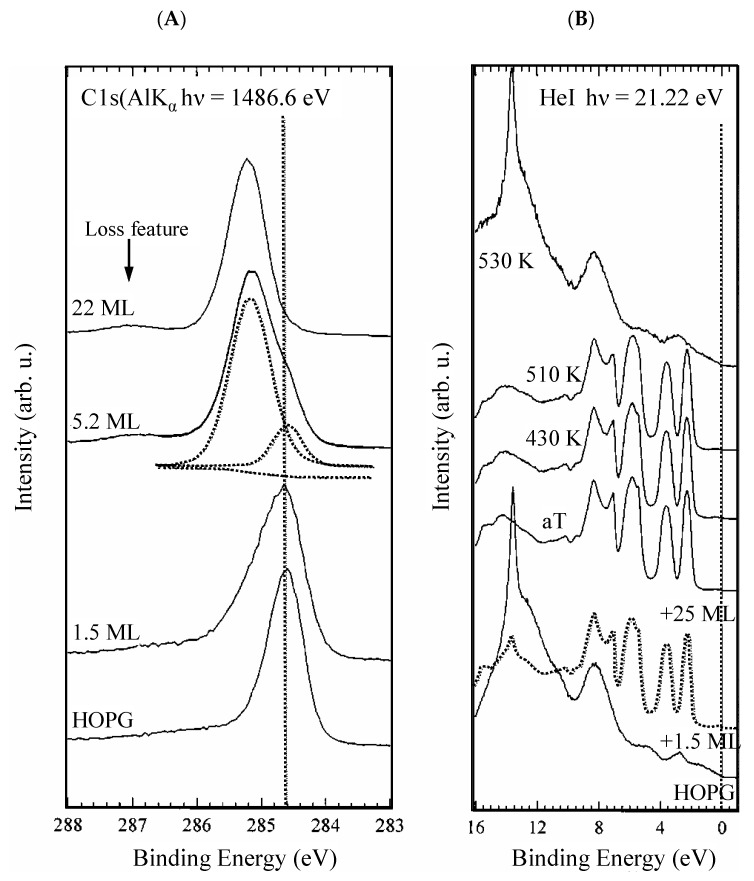
(**A**) Carbon 1s core level measured for the increasing coverage of the HOPG surface with C_60_. The bottom spectrum corresponds to the bare graphite surface. The deconvolution of the peak with Doniach–Sunic functions and a Shirley background (broken line) into two components (dotted lines) is included for the deposition step with 5.2 ML of C_60_. The agreement with the measured curve is excellent. (**B**) UPS-VB spectra recorded during the deposition of C_60_ and the subsequent annealing experiment. The bottom spectrum is the bare graphite surface and the VB spectra observed after the deposition of 1.5 and 25 ML of C_60_. Only a selection of UPS-VB spectra measured at a temperature close to the C_60_ desorption temperature is shown. The HOPG characteristics are completely restored at 530 K. Reprinted with permission from [157].

Figure 11A shows the C 1s from the HOPG (bottom) and the evolution with the deposition of increasing amounts of C_60_. As can be seen, the C 1s peak shifts to higher BE values, which are typical of CH_x_ aromatic molecules. The XPS analysis was carried out on a fullerene film. In this case, the semiconducting character of fullerenes results in poor conductivity. At room temperature, it is ~10^−14^ S/cm [152,153] and increases when fullerenes organize in a single crystal [152]. Quantum confinement effects leading to BE shifts to higher values may be expected [154,155,156]. Figure 11A shows also that, in correspondence to the higher amounts of deposited C_60_, it turns up a feature at 1.8 eV higher BE with respect to the C 1s, which can be assigned to the loss features associated with a monopole such as the HOMO-LUMO transition. In Figure 11B, the HeII valence band of the HOPG and the fullerene deposited on the HOPG at increasing concentrations are shown. The desorption of C_60_ at increasing temperatures and the corresponding HeII VB are also shown. The VB of C_60_ is much more structured, revealing a more complex electronic configuration. The lack of electron mobility, which causes a shift of the C 1s to higher BE, results in the formation of a gap here. The fullerene HOMO (top of the VB) is placed at a BE of 2.3 eV below the Fermi level, mirroring the fact that C_60_ is diamagnetic (no unpaired electrons) and does not conduct electricity.

Fullerenes behave as semiconductors [153] and display a temperature-dependent conductivity [153] induced by fullerene phase transitions [152]. Several theoretical studies were carried out in the past concerning the description of the electronic structure of the fullerenes [158,159,160,161,162]. As an example, the authors of [163] compare the DOS of C_60_, graphite and diamond using density functional calculations. The theoretical band structures are compared with the experimental valence bands with a high degree of correspondence. Using both the XPS valence spectra and the X-ray Kα emission spectra, it was possible to separate the contribution of the pσ- and pπ-bonding molecular orbitals. C KLL Auger spectra were also reconstructed, computing the 1s-2p2p, 1s-2s2p and 1s-2s2s transitions derived from the chemically different carbon atoms. Differently from inorganic crystals, the gap states in organic systems are strongly influenced by disorders and imperfections in the molecule packing. Gap states may be charge traps, thus directly influencing the transport properties of the material. The density of gap states of crystalline and polycrystalline organics can be obtained using current voltage [164] or Kelvin probe measurements [165]. However, the density of gap states can also be studied by angular resolved UPS, as in [166] for C_60_ crystals. 

The low photoelectron kinetic energy and fullerene conductivity lead to serious charging problems, which can be overcome by laser irradiation. The authors were able to estimate the ionization energy of a C_60_ single crystal (C_60_SC) and a C_60_ polycrystalline thin film (C_60_PC) deposited on a Si substrate, defined as

**Figure 12:**
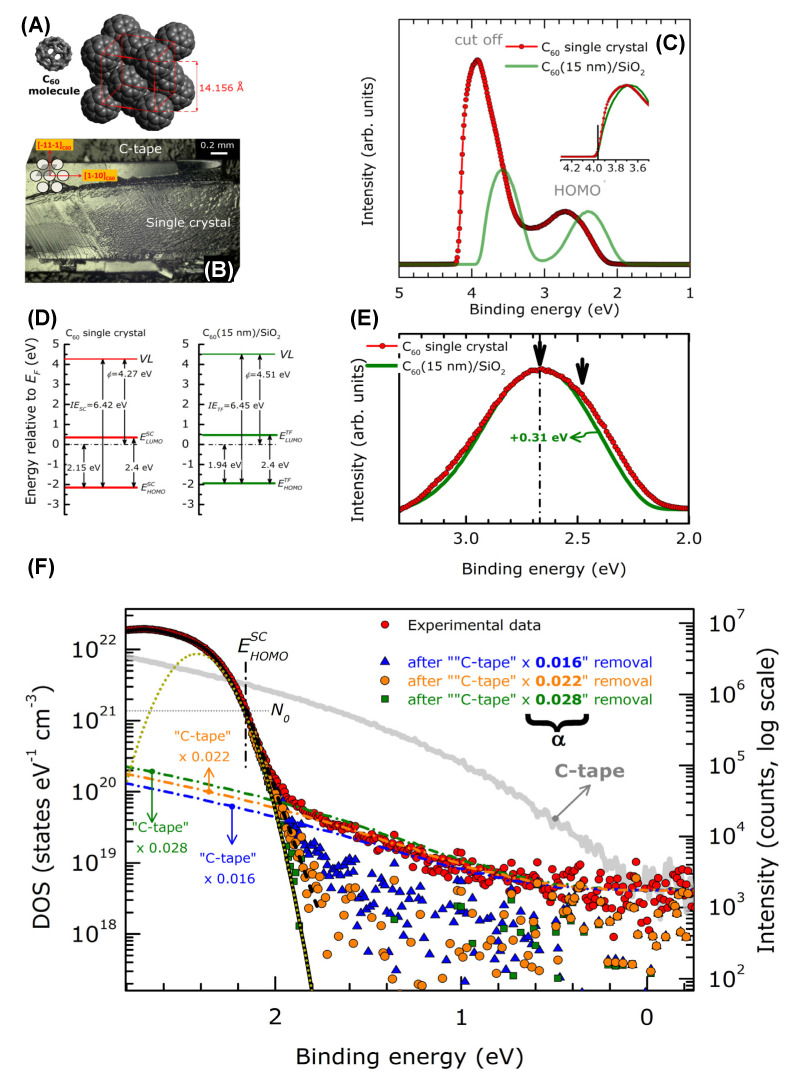
(**A**) Structure of the C_60_ molecule and its packing in the face-centered cubic (fcc) unit cell with a lattice parameter of 14.156 Å. (**B**) Photographic image of the C_60_SC on top of conductive carbon tape. (**C**) XeI-UPS-normalized spectra of the C_60_SC (red) and 15 nm thick C_60_PC on SiO_2_ (green). Inset: comparison of the C_60_SC and C_60_PC in the cutoff energy range. The UPS data were normalized at the highest intensity point of the cutoff region. The spectra are aligned to the cutoff position of the C_60_PC (vertical line). (**D**) Energy level alignment for the C_60_SC and C_60_PC as obtained from the UPS data in panel (**E**). (**E**) Comparison between the HOMO band of the C_60_ single crystal (red) and the C_60_PC (green) after the removal of the inelastic background. The UPS data were normalized to the highest intensity point of the HOMO bands and aligned at the HOMO peak position of the C_60_SC. (**F**) XeI-UPS spectrum of the C_60_SC in the HOMO and gap region (red). The XeI-UPS spectrum of the C-tape (gray) was rescaled to reproduce the C_60_ data in the 0–1.7 V BE range. The rescaled C-tape spectra (for different rescaling factors α) are indicated by dash-dotted lines. Continuous lines are the cumulative fitting curve of the C_60_-HOMO band resulting from the convolution of Gaussian functions. For clarity, only the lower-binding-energy Gaussian component was shown (dark yellow short dotted curve). Reproduced with permission from [166].

IE = E_HOMO_ + Φ = E_HOMO_ + hν − E_cutoff_ (11)

where E_HOMO_ is the BE value of the valence band top referred to the Fermi level, Φ is the work function, hν is the photon energy and E_cutoff_ is the minimum kinetic energy of the photoelectrons from the C_60_ systems. The results are shown in Figure 12. In Figure 12A, the structure of the C_60_ molecule organized in a face-centered cubic (fcc) structure is reported, while in Figure 12B, an image of the fullerene crystal cleaved on top of a carbon tape substrate is shown. Figure 12C reports the XeI UPS spectrum of both C_60_SC and C_60_PC film. The VB of the C_60_PC has a similar shape to that of the C_60_SC, but it is shifted towards the Fermi level of 0.21 eV. In Figure 12D, a schematic of the energy level is shown. The vacuum level (VL) and the HOMO edge positions of C_60_SC and C_60_PC were evaluated by the linear extrapolation of the secondary cutoff and HOMO bands. The LUMO positions were determined from the value of the HOMO–LUMO energy gap of C_60_ (2.4 eV) [167]. In Figure 12E the HOMOs of the C_60_SC and C_60_PC are compared. The latter appears to be more symmetric, while that of C_60_SC is composed by two bands derived from the non-equivalent molecules in the unit cell. Finally, Figure 12F displays the HOMO and gap C_60_SC and C_60_PC. The right axis represents the intensities on a logarithmic scale to highlight the very low intensity features, while the left axis describes the density of the states. In the 0–1.7 eV BE range, a very weak UPS signal derives from the disorder-related density of gap states (DOGS) and from the photoemission from the C-tape surrounding the samples. Three Gaussian functions were used to describe the electron–phonon coupling, the structural disorder and the small energy band dispersions [166]. The total DOS value of C_60_SC at the HOMO edge is N_0_ = 1.42 × 10^21^ states eV^−1^ cm^−3^ (gray horizontal dotted line). This value is used to define the C_60_SC HOMO edge, which falls at 1.7 eV. Above this energy, the VB starts to deviate from the Gaussian function, and the DOGS decreases very rapidly (black short dashed line) from N ~ 10^21^ to N ~ 10^19^ eV. In the studied C_60_SC, the detection limit of the DOGS can be roughly estimated to be ~10^18^ states eV^−1^ cm^−3^. 

**Figure 13:**
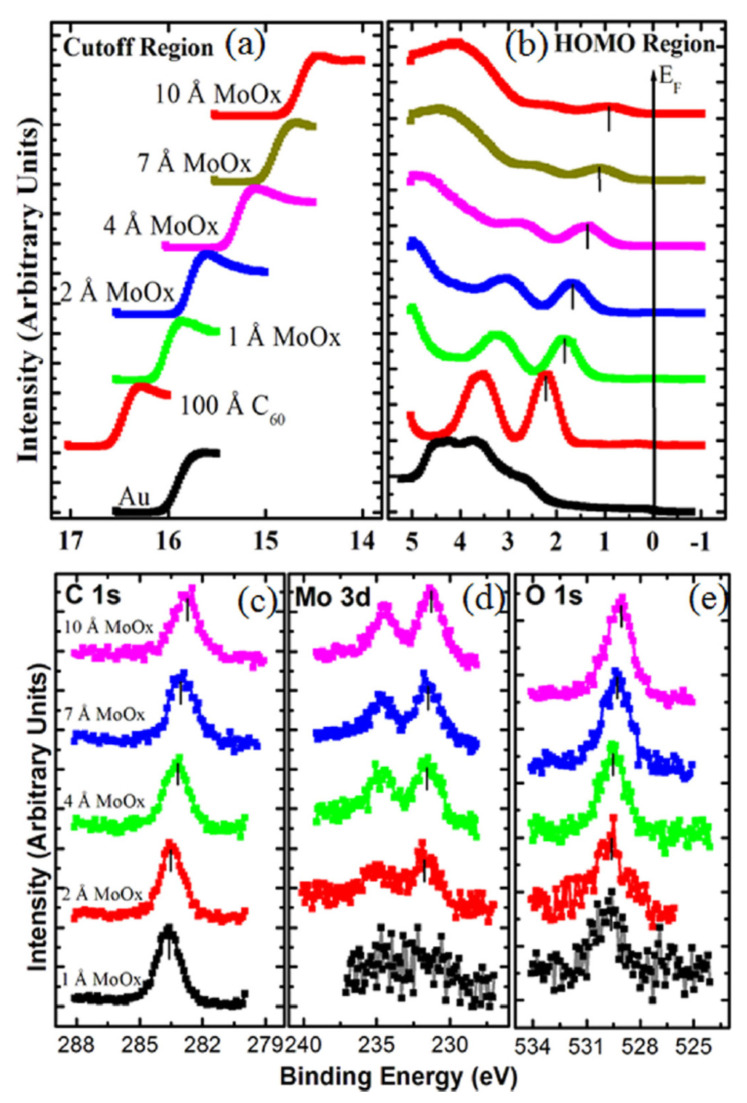
UPS and XPS spectra evolution of MoOx/100Å C60/Au. (**a**) The cutoff region; (**b**) the HOMO region; (**c**) C 1s, (**d**) Mo 3d and (**e**) O 1s core levels. Reproduced with permission from [168].

Photoelectron spectra can also be utilized to describe the effect of C_60_ doping. In [168], the effect of MoO_x_ on the electronic structure of the C_60_ deposited on a gold substrate is investigated by Mg Kα and HeI radiations. As can be seen in Figure 13a,b, both the cutoff and the HOMO position evolve with the increase in the amount of MoO_x_ on a C_60_ film. As can be seen, by increasing the amount of MoO_x_ doping, the HOMO shifts towards the Fermi level. Concurrently, the cutoff position lowers, thus resulting in an increase in the work function from 4.68 eV of C_60_ on Au to 6.48 eV when the MoO_x_ thickness reaches 10 A°. In Figure 13c–e, the C 1s, Mo 3d and O 1s are shown, respectively, whose intensities increase by increasing the MoO_x_ deposition.

As for the previous nanostructures, XPS is extremely useful in controlling the fullerene chemistry. An overview of the possible methods and kinds of functionalization is reported in [169,170,171,172]. Functionalized fullerenes are utilized in different kinds of applications. In particular, the lack of the regular delocalization of charges makes C_60_ an electron-deficient system. This system easily reacts with electron-rich molecules. This property is exploited in photovoltaics to reduce recombination at the donor–acceptor interface [173] in photobiology [174,175], where photoinduced reactions are based on the charge separation provided by fullerenes, in photochemistry, where the charge transfer is at the basis of Dies–Alders reactions, in medicine [176] and in photodynamic therapy [177]. One problem commonly found, is the scarce solubility of fullerenes, preventing their dispersion in aqueous solvents. This problem is solved by functionalizing the fullerene surface, rendering them hydrophilic [178]. There is a huge number of works in which XPS is used to characterize functionalized fullerenes. Here, we will give only some examples. In [179], a variant of Staudenmaier’s method was utilized to graft oxygen-based functional groups on the fullerene surface. XPS was utilized to check the efficiency of the reaction, leading to an improved degree of hydrophilicity. Fullerene can be functionalized with cyanuric chloride molecules by using a nitrene-mediated cycloaddition reaction. XPS revealed the presence of extrinsic atoms such as N and Cl. The C 1s analysis led to the identification of two components assigned to triazine molecules (56%) and fullerene molecules (44%). This is consistent with the conjugation of five triazine molecules per fullerene unit. Fullerene derivatives possessing high electron-accepting properties are widely utilized in planar heterojunction perovskite solar cells as electron transport layers. In [180], the authors synthesized pyridine-functionalized fullerene derivatives using a 1,3-dipolar cycloaddition reaction. By varying the nitrogen site, the authors were able to produce three different molecules. The molecular structures and packing were determined by X-ray single crystal diffraction. Using XPS, the authors found that the electron transport properties of the material were dependent on the nitrogen site within the pyridine molecule, resulting in a different coordination interaction between the Pb^2+^ ion of the perovskite layer and the pyridine moiety. In another work [181], the organic solar cells fullerenes enhanced the performances of the PTB7-Th:PC71BM active layer. The authors synthesized two different fullerene derivatives functionalized with amphiphilic diblocks—namely, C_60_-2DPE and C_60_-4HTPB. The modified fullerenes were deposited as an interface on a zinc oxide (ZnO) layer. The two fullerene buffers led to an improvement of the coupling between ZnO and the active layer, leading to a power conversion of 9.21% and 8.86% respectively. In that work, XPS was used to analyze the ITO-ZnO, ZnO/C_60_-2DPE and ZnO/C_60_-4HTPB samples, confirming the good synthesis of the two amphiphilic diblocks. Finally, UPS was utilized to investigate the material electronic properties. In particular, the authors determined the HOMO binding energy position for the two different solar cells and, knowing the bandgap, the LUMO position as well as the cell work functions, which were correlated with the cell energetics. Fullerenes are also used to increase the efficiency in the generation of singlet oxygen, as shown in [182]. C_60_ was functionalized with selenophene and electrochemically co-deposited with bis-selenophene on platinum (Pt) or indium-tin oxide (ITO) substrates. Different properties of the monomers in the deposited film led to different spectroscopic and photosensitizing properties. XPS used to control the layer chemistry was utilized to optimize the ratio between C_60_ photosensitizers and organic units in the layer, thus allowing the authors to improve the efficiency of the visible light-driven singlet oxygen generation. 

**Figure 14:**
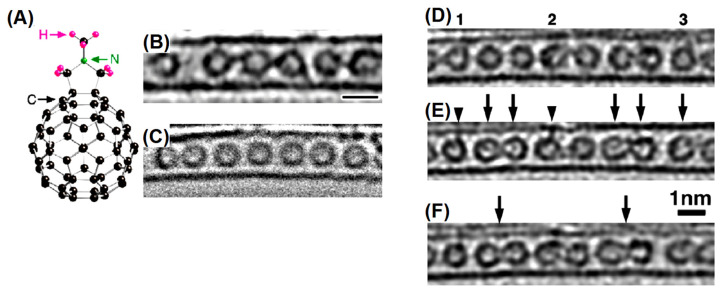
(**A**) A scheme of the used fullerene derivatives functionalized with a pyrrolidine (C60-C3NH7, N-methyle-3,4-fulleropyrrolidine). HR-TEM images of a functionalized fullerene peapod (**B**,**C**) and the non-functionalized fullerene peapod. A sequential HR-TEM image of (C_60_-C_3_NH_7_)_n_@SWNTs. The functional group attached to each fullerene cage is indicated by arrows and arrowheads. (**D**) 0 s (**E**) 2 s and (**F**) 4 s. Some of the fullerenes make rotations inside the SWNTs. Two of the adjacent fullerenes begin to fuse with each other (**F**). Reproduced with permission from [183].

Interestingly, functionalized fullerenes may be visualized using HRTEM [183]. An example is reported in Figure 14. CNT is utilized as a fullerene container, which limits the damage induced by the electron beam in the carbon nanostructures and, at the same time, limits the fullerene motion, which results in a higher image quality. Figure 14A shows C_60_ molecules functionalized with C_3_NH_7_ via the Prato reaction, resulting in a N-methyle-3,4-fulleropyrrolidine (Figure 14A). It can be observed that the interference caused by the upper side of the SWCNT wall was minimized to obtain a clearer image of the inner fullerenes. This allows for the discrimination between the functionalized fullerenes (Figure 14B) and the reference fullerenes (Figure 14C), where the functional groups are visible. In Figure 14D–F, the dynamic behavior of (C_60_-C_3_NH_7_)_n_@SWNT is shown. HRTEM images are acquired with an interval of 2 s between each image. Figure 14D is acquired at time = 0 and reports the reference fullerenes. In Figure 14E, line-shaped (approximately 0.25 nm long) pyrrolidine-type functional groups can be seen at the outside of each C_60_ (indicated by the arrowheads). The functional groups can often appear in the middle of the fullerenes (marked by the arrows) because they are located between the nanotube and the fullerene in the projected HR-TEM image. At higher times, Figure 14F shows the sensitivity of the fullerenes to the electron beam. Indeed, compared to the non-functionalized fullerenes, functionalized C_60_ are more sensitive to the electron beam and are more easily fused or coalesced with each other. It can be observed that this reaction does not proceed through their functional groups but via the fullerene bodies (see the arrows in Figure 14F). 

### 4.4. Surface Chemistry and Properties of Nanodiamonds

**Figure 15:**
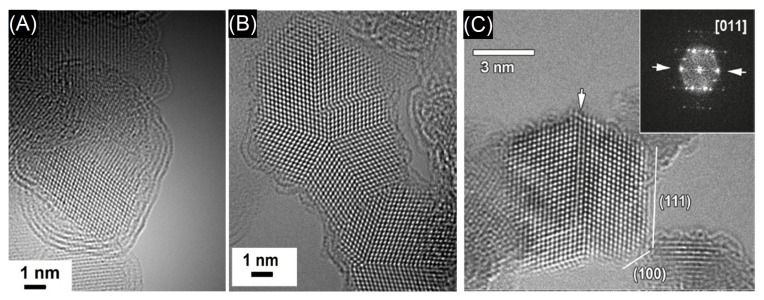
(**A**) A nanodiamond cone. (**B**) Nanodiamonds characterized by a twinned structure. Reprinted with permission from [192]. (**C**) An ozone treated detonation nanodiamond. The non-diamond phases are negligible. The inset shows clear diffraction patterns of the diamond crystal lattice. Reprinted with permission from [191].

The functionalization of the surface is also commonly utilized to characterize nanodiamonds (NDs). These nanoobjects can be synthesized by massive fragmentation bulk diamonds by high energy ball milling [184,185]. The effects of the high pressure applied to induce the fragmentation were studied in [186]. The authors found that, by increasing the pressure, it is possible to obtain a rather homogeneous ND size. The properties of the NDs mirror those of the bulk diamonds in terms of biocompatibility and mechanical, optical, thermal and electrical properties. Diamond is characterized by sp^3^-hybridized carbon atoms arranged in a tetrahedral structure. This structure leads to face-centered cubic or hexagonal (lonsdaleite) lattices, which result in the higher resistance to compression among the materials ranging from 90 to 225 GPa depending on the crystal orientation. Diamond is classified as a wide bandgap material with a prominent resistivity of 10^11^ to 10^18^ Ω·m and a prominent phonon mobility, which results in a high thermal conductivity of 3320 W/(m K) at RT. The strong C–C bonds and the absence of free electron pairs induce a very low chemical reactivity, even in presence of strong acids. Finally, diamond possesses a high refracting index from 2.465 in the violet region to 2.409 in the red region. In diamond, the absorption is caused mainly by different colored centers induced by extrinsic elements such as nitrogen, boron, phosphorous, hydrogen, nickel, cobalt, silicon, germanium and sulphur. Nitrogen is the more common color center, leading to different defects classified as A, B, C N2 and N3 centers. NDs can be synthesized via CVD processes [187,188], which are used to produce high quality crystals from nanometric to macroscopic dimensions. NDs may also be synthesized by laser ablation in liquids [189,190]. Nanometric-sized diamonds are produced by detonation processes produced by mixing trinitrotoluene (TNT) and cyclotrimethylenetrinitramine (RDX) in a closed chamber. The high temperature and pressure caused by the explosion process leads to the formation of diamond crystals with a typical average size of about 5 nm [3]. All these synthesis processes may introduce graphitic or amorphous matter or induce surface oxidation. HRTEM and XPS are then the probes of choice to detect the presence of undesired non-diamond phases. In Figure 15, an example of detonation nanodiamonds is illustrated, where the HRTEM image clearly shows the presence of graphitic shells containing diamond nanocrystals and, in particular, Figure 15A shows a big diamond cone, while a nanodiamond with twins is displayed in Figure 15B. The graphitic shells are commonly removed by using ozone or acid treatments attacking the graphitic phase at the defects. Thanks to the high chemical inertness, the diamond core is preserved. The effect of ozone cleaning is shown in Figure 15C, where the graphitic phase has disappeared and a purified nanodiamond crystal is left [191]. This also appears in the inset, where a perfect diffraction pattern corresponding to the diamond lattice is reported.

In [193], the authors utilized the C KLL Auger feature obtained by X-ray photoemission to evaluate the sp^2^ and sp^3^ carbon nanostructures, including NDs. The estimation of the sp^2^, sp^3^ hybridization of carbon atoms may be performed by analyzing the XPS C 1s spectrum, relying on the different BE values of graphite at 284.4 eV and of diamond at 285 eV. However, some serious problems may arise because of the interference of chemical bonds overlapping the two prototypal sp^2^ and sp^3^ components. The main source of error can derive from the CH_x_ bonds, which fall at the same BE of the diamond, generating a complete interference. There is no way to discriminate the CH_x_ contribution from the diamond phase from CH_x_, and this is a non-trivial problem considering that hydrocarbon is always present as a surface contaminant or as a residual product of the synthesis process. The C 1s analysis is also complicated because the graphitic phase should require a Doniach–Sunjic line shape with a pronounced tail overlapped with the diamond component. If amorphous carbon is present, Gaussian line shapes are utilized for fitting. Again, it is very difficult, if not impossible, to estimate the presence of sp^2^ crystalline phases from the amorphous matrix and the relative intensities of the Doniach–Sunjic and the Gaussian contributions. Finally, the presence of carbon–oxygen bonds and, in particular, the hydroxyl bond may also affect the C 1s peak fitting. For this reason, the C 1s fit is coupled with the analysis of other spectral features such as the C KLL Auger spectrum. In [193], the authors utilized the C KLL to discriminate the sp^2^ from the sp^3^ phases. A similar analysis was performed by other authors [194], who analyzed the C 1s, its loss features and the C KLL Auger spectrum of acid-cleaned detonation NDs, which were subsequently annealed at 900 and 1500 °C. 

**Figure 16:**
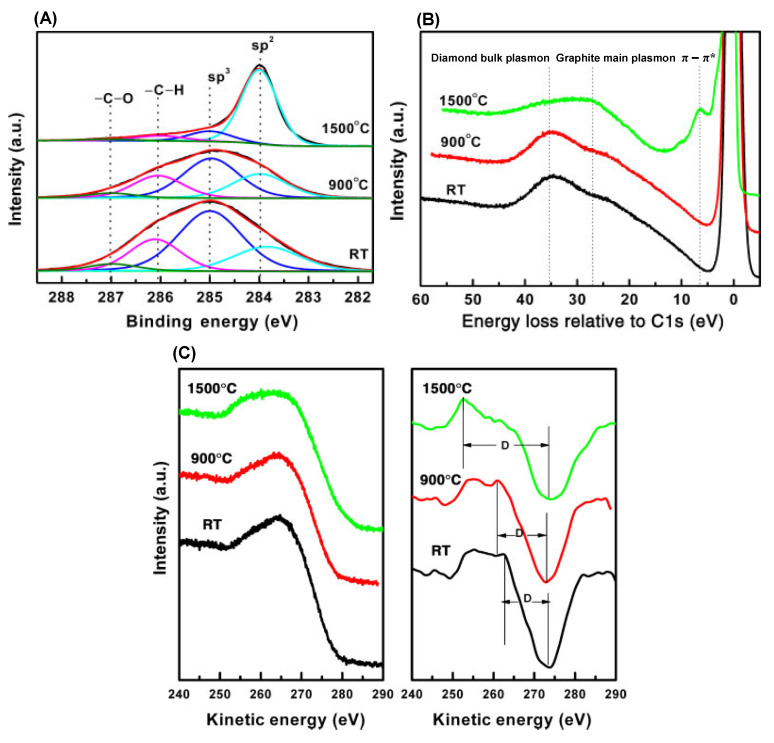
(**A**) High-resolution XPS C 1s peak of the unannealed ND powder and the ND powder annealed at different temperatures, with fitting curves. (**B**) C 1s energy-loss spectra of the unannealed sample and the samples annealed at different temperatures. (**C**) C KVV X-ray-excited C KLL Auger spectra (**Left**) and their first derivatives (**Right**), as compared with the unannealed sample and the samples annealed at different temperatures. Reprinted with permission from [194].

Figure 16A shows the deconvolution of the C 1s in the sp^2^ and sp^3^ and in the carbon–oxygen bond components. Here, it is clearly seen how the fitting components interfere with each other. Figure 16B reports the different line shape of the loss feature depending on the carbon hybridization. An analysis of the loss components may be carried out, as shown in [195]. Finally, Figure 16C shows the evolution of the Auger KLL of ND powder induced by the annealing. The high temperature leads to an increase in the D parameter, which is associated with a progressive graphitization of the ND powder. The C 1s analysis was also utilized in another work [187] to understand the effect of different synthesis parameters on the quality of NDs. The latter were synthesized using novel continuous atmospheric microplasma in an atmosphere produced using just 180 p.p.m. ethanol vapor in Ar or 180 p.p.m. ethanol vapor mixed with 450 and 10,000 p.p.m. H_2_ gas in Ar. The deconvolution of the C 1s peak in the sp^2^ and sp^3^ components was sensitive to the different plasma atmospheres, showing that the presence of H_2_ is favorable for the formation of sp^3^ hybrids.

**Figure 17:**
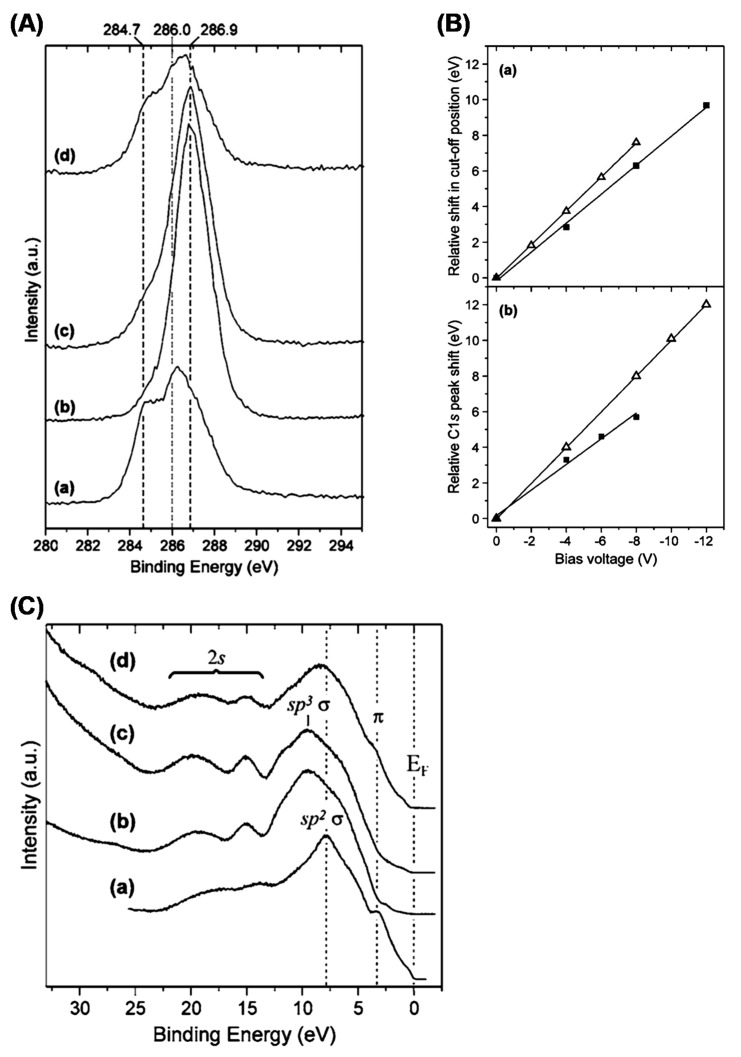
(**A**): C 1s photoemission spectra of (a) the initial ND sample; (b) the same sample after atomic hydrogen treatment; (c) the sample following additional annealing at 1000 K after hydrogenation; (d) the sample following additional annealing at 1200 K after treatment described in (c). The annealing time was 20 min for both temperatures. Dashed lines are used to guide eyes. (**B**): (a) E_cutoff_ and (b) C 1s peak position of the sample as a function of applied negative bias voltage after the sample was treated with atomic hydrogen (full squares) and after annealing at 1000 K (open triangles). (**C**): Valence band spectra of (a) the initial ND sample annealed to 1420 K, (b) the same sample after atomic hydrogen treatment, (c) the additional annealing at 1000 K after hydrogenation, (d) additional annealing at 1200 K after the treatment described in (c). Dashed lines are used to guide eyes. E_F_ is the Fermi energy level. The spectra were obtained using a photon energy of 55 eV. Reproduced with permission from [199].

In general, the estimation of the sp^2^ and sp^3^ hybrid abundance is rather complex, and all the spectral features—the C 1s, valence band, C KLL Auger and loss features—may help to obtain more reliable values, as shown in [195,196], or different techniques providing information of the different physicochemical properties of the material, as in [197]. The effect of annealing and the progressive transformation of nanodiamonds into onion-like carbon nanostructures was investigated by XPS in [198]. The authors carefully studied the changes in the photoelectron C 1s and valence band spectra upon the UHV annealing of nanodiamonds. The results show that thermal treatments at high temperatures generate a progressive growth of graphitic layers on the surface of the ND crystals by the transformation of the C-hybridization from sp^3^ to sp^2^. This change is mirrored in the C 1s spectrum by a downshift of the C–C components from that of the diamond towards those of the graphite. A similar fate is observed in the valence band, which also displays a modification of the DOS features, mirroring a gradual graphitization. The effect of annealing and of hydrogenation on the electronic structure of NDs is investigated in [199]. The authors studied the effect of hydrogen treatment under UHV, aiming to etch the graphitic layers and, at the same time, to saturate dangling bonds to avoid ND aggregation. It is worth noting that hydrogen-terminated NDs display negative electron affinity [200,201], p-type surface conductivity [202] and superb biocompatibility [203]. The results of the hydrogenation process and annealing are shown in Figure 17. Figure 17A displays the evolution of the C 1s peak, which is initially formed by two components assigned to the sp^3^ C-hybrids derived from the diamond core and the sp^2^ C-hybrids derived from the graphitic layers. Hydrogenation efficiently etches the graphitic shell, and a pure diamond core is left. Upon thermal annealing at 1000 and 1200 °C, the NDs start to graphitize, as reflected by the increasing intensity of the component at 284.7 eV. In Figure 17B, the shift of the cutoff energy and of the position of the C 1s peak upon the application of a bias is shown. For the hydrogen-terminated NDs, the energy shift is not proportional to the applied bias, revealing a positive surface charge upon X-irradiation. This explains the higher energies found for the C 1s peak with respect to the current literature (see, for example, [204] and the references therein). Finally, in Figure 17C, the changes in the density of states of the pristine, hydrogenated and annealed NDs using hν = 55 eV radiation are reported. Initially, the valence band shows main peaks located at 3.2 and 7.7 eV, assigned to the π and σ bands of graphite, respectively [205,206]. In the hydrogenated NDs, a feature at 9.5 eV that is derived from the C 2p states and peaks at 15.1 and 19.5 eV that are attributed to the C 2s states appear [114]. The disappearance of the π structure at 3.2 eV with hydrogenation reflects the etching of the graphitic layers from the NDs. On the contrary, annealing leads to an increase in this feature, mirroring an increasing graphitization.

Photoelectron spectroscopies are also very useful in estimating the electron affinity of materials. Diamond is a wide bandgap material, however, with appropriate surface termination, it behaves as a semiconductor, possessing a highly stable negative electron affinity (NEA) [200]. 

Generally, diamonds display a positive electron affinity (PEA). In other words, electrons excited by radiation into the conduction band minimum (CBM) encounter an energy barrier, the electron affinity χ, which must be surmounted to raise the electron to the vacuum level. However, in the case of H-terminated diamond, the χ is negative: the electrons at the CBM are free to leave the diamond surface. 

**Figure 18:**
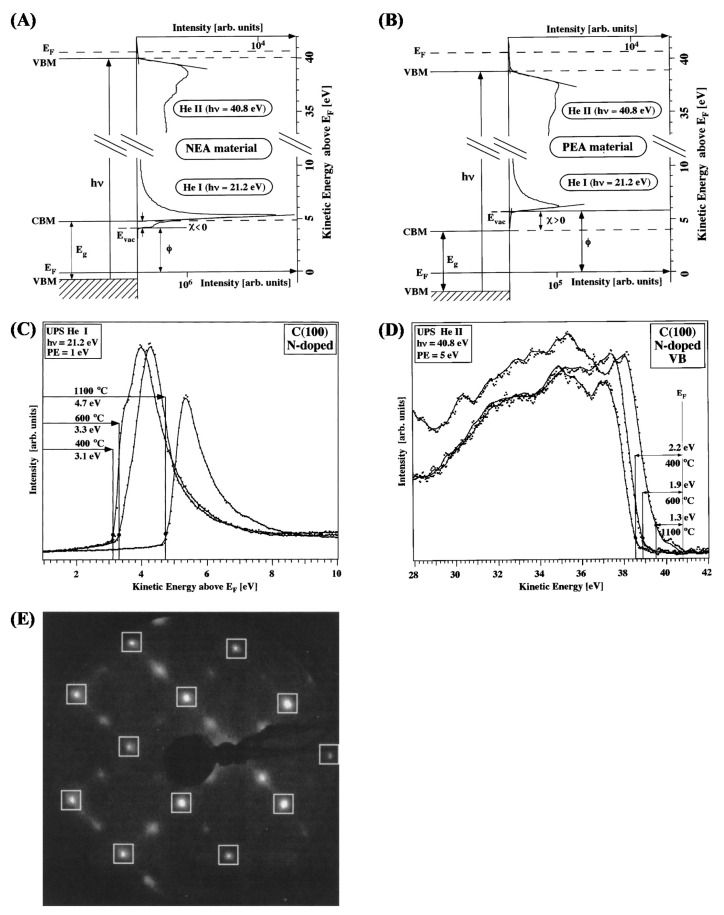
Combined He I (hν = 21.2 eV) and He II (hν = 40.8 eV) normal emission spectra of the valence band for (**A**) negative electron affinity (NEA) and (**B**) positive electron affinity (PEA) materials with the band diagrams on the left side, respectively. We determine the energy levels at the cutoff positions with extrapolation to zero intensity (**C**). Labeled are the work function W, the electron affinity χ, the band gap E_g_, the vacuum level E_vac_, the photon energy hν, the Fermi level E_F_, the conduction band minimum (CBM) and the valence band maximum (VBM) shown in (**D**). Note the different intensities for the spectra of the (**A**) NEA and (**B**) PEA cases. (**E**) LEED pattern (E = 154.7 eV) from the hydrogen plasma-treated N-doped diamond (100) surface, which reveals a (2 × 1) reconstruction. The spots corresponding to a bulk termination are marked by white squares, while the other spots characterize the two domains of the (2 × 1) superstructure. Reprinted with permission from [213].

The consequence is that, upon irradiation, the yield of the photoelectrons of the H-diamond exceeds that of PEA surfaces by orders of magnitude [207,208], making diamond an interesting material for cold cathode emitters, photocathodes and other electronic applications [209,210]. The mechanism used to determine the entity of the χ is shown in the diagram in Figure 18A. UPS HeI and HeII photons can be utilized to determinate the electronic structure of the diamond samples. When a diamond surface shows a negative χ (Figure 18A), electrons excited into the CB are emitted very easily into the vacuum because electrons thermalizing in the CBM are above the vacuum level. To determine the NEA, He I photons are used to find the cutoff position at low kinetic energies, because the cross-section of the 2p states at a 8.0 eV binding energy is higher than that for photons of a 40.8 eV energy. Figure 18B shows the band diagram for a positive electron affinity (PEA). The cutoff position is estimated with a linear fit on the tail at a lower kinetic energy, determining the minimum energy required to excite electrons in the CBM, as shown in Figure 18C. The CBM is calculated from the position of the valence band maximum plus the energy gap E_g_, which, for diamond, is 5.5 eV. He II photons are generally used to map the states near the Fermi edge (top of the valence band) due to the higher cross-sections of these states with respect to He I. The VB maximum is extrapolated as the point where the linear fit on the descending shoulder meets the background level in the region near the Fermi edge, as shown in Figure 18D. These measurements may be performed for both negative and positive electron affinity surfaces, as schematized in Figure 18A,B. In the case of PEA, the position of the CBM is below the vacuum level E_vac_, as shown in Figure 18B. Electrons thermalized in this level need an extra amount of energy to win the surface potential and be emitted to the vacuum. Finally, the quality of the diamond samples may be controlled by the LEED patterns reported in Figure 18E. In nanodiamonds, the situation is more complicated, since the small size may induce quantum confinement effects. The dependence of the energy band gap on the size was studied by theoretical models [211]. More recent studies found that surface states are generated in low-dimension diamonds. As the number of atoms of a diamond cluster increases, the HOMO transforms in the valence band of a bulk diamond. A different fate occurs for the LUMO, which does not turns into the diamond conduction band. By increasing the ND size, the LUMO mutates into unoccupied surface states, which, for diamond, are located between 2.0 and 3.3 eV above the VB and below the CB, respectively [212]. Difficulties are also added considering the possible presence of graphitic layers due to the ND synthesis, which have to be removed.

The surface termination, as in the bulk diamond, has a fundamental role. In [214], the authors found that the emission properties of hydrogenated NDs can be described with a two-barrier model where electrons are injected into the NDs from a conductive electrode. Then, they propagate along the ND surface and escape to the vacuum thanks to the NEA. In oxidized NDs, the presence of oxygen increases the surface barrier, thus shifting the electron emission at a higher potential. In [215], the authors used XPS and UPS to characterize the NDs. The C 1s peak indicates that NDs possess n-type doping behavior. The FWHM of the C 1s is 2.15 eV, is rather broad due to the presence of non-diamond phases, which was also proven by Raman spectra. Finally, UPS was utilized to estimate the electron affinity, which, after H plasma treatment, was 0.2 eV. 

As for the previous carbon nanostructures for NDs, the surface chemistry directly influences the electronic properties of the material, as seen above. The synthesis methods also influence the surface chemistry of the final material and thus the kind of interaction with the environment. Generally, after the synthesis of the NDs, deagglomeration, purification and fractionation processes are applied to eliminate non-diamond phases and contaminants and homogenize their surface chemistry and size distribution. The etching of the graphitic phases in detonation NDs, implanted NDs or metallic contaminants in HPHT diamonds is conducted using purification protocols based on different acid mixtures (for example, HCl/HNO_3_, H_2_O_2_/NaOH) [216], leading to surface oxidation. Purification is also performed through gas phase treatments—for example, oxygen, hydrogen plasmas, ozone and hydrogen. After purification, the functionalization of the ND surface is applied to thinly control the behavior of the particulate (dispersivity, capability to adsorb specific molecules, interaction with organic substances, lubricant properties, etc.) in relation to the desired application. Based on the selected purification processes, the ND surface is oxidized or hydrogenated. Wet chemistry can be applied to reduce the oxidized surface in order to enhance the abundance of hydroxyl groups [217]. The activation of the ND surface with hydroxyl groups opens a series of possible reactions with organic molecules. For example, milder reactions are based on the use of carbodiimide/succinimide activation, enabling reactions in water or buffers at room temperature [218]. This functionalization has been successfully applied to graft a large variety of molecules such as small organic molecules, polymers, proteins and dyes. Another possibility is the use of carboxyl groups for esterification reaction. Carbodiimides and appropriate bases are used to activate the ND surface, which can be subsequently functionalized with the desired alcohol moieties such as polyglycerol or polyethylene glycol [219]. Esterification is also used to graft biomimetic coatings in order to control opsonization [220] and avoid immune system reaction, enhancing the blood compatibility [221,222]. Another possible reaction is the modification of the ND surface through silanization, which offers the advantage of being performed using mild alcohols as solvents and a large selection of siloxanes. Stable and uniform crosslinked siloxane coatings may be obtained using 1,2-bis(triethoxysilyl)ethane [219]. For their versatility, silanized surfaces were used, for example, to deliver genetic matter [223], to attach dye molecules and receptors [224] and for the interaction between the filler and the polymer matrix host [225]

The hydroxyl-functionalized ND surface can react with alkyl groups [226,227], while hydrogenated NDs can be functionalized with alkenes or aminated using diazonium salts [227,228]. The oxidation or hydrogenation of ND surfaces followed by covalent or non-covalent functionalization routes can be used to graft a large multiplicity of molecules. More information can be found in [228,229,230]. Besides other characterization techniques such as Fourier Transform Infrared Spectroscopy (FTIR) or direct tritration, XPS is a very useful and helpful analytical technique to assess ND surface chemistry. Pristine and functionalized NDs can be analyzed to determine the success of a chemical processing applied to graft desired molecules. Elemental quantification is commonly used to establish the efficiency of the chemical reactions used. As an example, in [231], the authors used the combined results of XPS, FTIT and Nuclear Magnetic Resonance (NMR) to unambiguously characterize the surface chemistry of detonation NDs treated in a reduction reaction. The ND surface was enriched with hydroxyl and hydroxymethyl functional groups. Characterization techniques and quantum-chemistry modeling demonstrated that samples needed a vacuum treatment to completely remove the adsorbed water and other volatile contaminates in order to obtain a correct description of the chemistry and a quantification of the hydroxyl groups on the detonation ND surface. In [232], grinded and detonation NDs were analyzed by XPS to characterize their composition and the surface chemistry. Acid treatment was utilized to remove surface contaminants, and the abundance of nitrogen, sulfur, chlorine and metal atoms was negligible. 

C KLL Auger and core line spectra describe an oxidized ND surface with a concentration of oxygen of ~9%. The analysis of the C 1s allowed for the estimation of the sp^2^/sp^3^ ratio, which varied from 0.58 to 0.26 for detonation and milled ND, respectively. Finally, a survey of the use of XPS to characterize the ND surface terminations, the biomolecule or the polymer grafting is presented in [233]. This work also describes the use of XPS to investigate the in situ reactivity and the stability of NDs toward various atmospheres such as plasma or UHV annealing.

### 4.5. Carbon Dots

Carbon quantum dots (CQDs) were discovered as byproducts of a top-down route used to cut large carbon nanostructures such as graphite, long CNTs, micron-sized graphene patches, etc. in smaller particulates [234]. The process is performed in strong acids, resulting in the production of nanometer-sized CQDs, which are, at the same time, functionalized with oxygen groups such as hydroxyl and carboxyl groups. 

**Figure 19:**
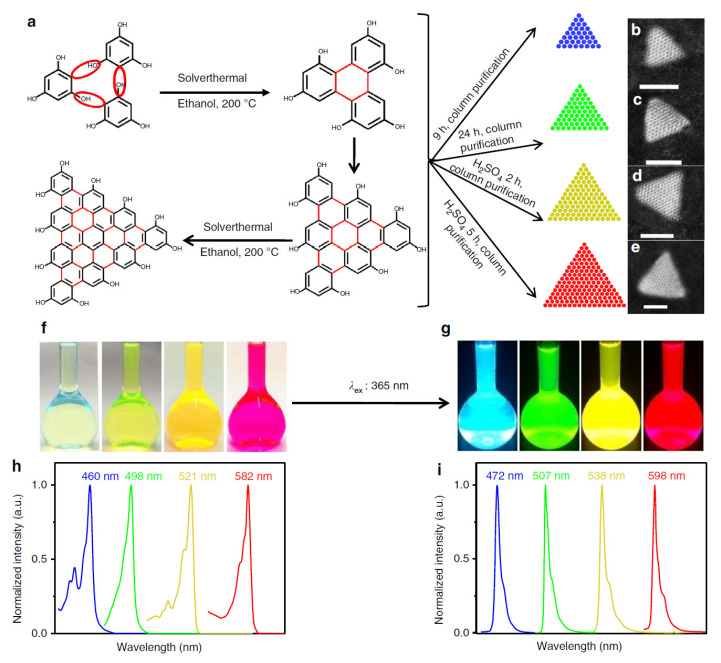
Design and synthesis of narrow bandwidth emission triangular CQDs. (**a**) Synthesis route of the NBE-T-CQDs by the solvothermal treatment of phloroglucinol triangulogen. The typical aberration-corrected HAADF-STEM images of B- (**b**), G- (**c**), Y- (**d**) and R-NBE-T-CQDs (**e**), respectively. Scale bar: 2 nm. Photographs of the NBE-T-CQDs ethanol solution under daylight (**f**) and fluorescence images under UV light (excited at 365 nm) (**g**). The normalized UVvis absorption (**h**) and PL (**i**) spectra of B-, G-, Y- and R-NBE-T-CQDs, respectively. Reprinted with permission from [240].

Recently, several theoretical and experimental studies have been performed to explore the fundamental properties of Dirac fermions, such as Klein tunneling [235,236], quantum electron optics [237,238] and electron–electron (e–e) interaction [239]. As observed for the other C-nanostructures, in this case, the confinement of electrons in nanosized systems results in peculiar properties. One example is the possibility to tune the frequency emission from CQD, as shown in [240]. The authors were able to reduce the broad emission of CQD obtained from graphene by synthesizing triangular nanostructures (Figure 19a–e). Differently from optical properties related to surface defects, in these systems, the absorption and emission bands originate from the creation of excitons and from exciton recombination, respectively. By increasing the CQD size from 1.9 nm to 3.9 nm, the emission frequency shifts from 472 nm to 598 nm (Figure 19h–i). These exceptional optical properties were investigated by several techniques, including XPS and UPS. The authors found that, with the different absorption bands, by going from the blue (472 nm) to the red emission (598 nm), the bandgap reduces from 2.63 eV to 2.07 eV, demonstrating the bandgap size dependence that has already been observed in other works [241]. Photoelectron spectroscopies were utilized to further support these results. UPS registered an up-shift in the HOMO levels from −5.18 eV to −4.92 eV and a correspondent down-shift of the LUMO orbital from −2.55 eV to −2.85 eV. In addition to FTIR and NMR, XPS provided important information on the edge termination of the triangular shaped CQD. The presence of electron-donating hydroxyl groups at the edge sites enhanced the emission properties, limiting the electron–phonon coupling and then narrowing the bandwidth. Theoretical calculations supported this interpretation. 

In [242], the authors synthesized nitrogen-doped CQDs to improve their optical properties induced by surface passivation and/or incorporation of the dopant into the dot core. The CQDs were synthesized using atmospheric pressure microplasma. Depending on the synthesis conditions, the CQDs were found to contain a variable concentration of N in a highly crystalline matrix. The synthesized CQDs displayed an energy band structure suitable for their use in solar cells. Differently from other devices based on Pb or carbon dots used in combination with polymers, here, the CQDs are used as a photoactive layer without the need for additional polymer composites or sensitizing materials [242]. The device is formed by an Indium Tin Oxide thin film, a TiO_2_-active electrode used as a hole blocking layer coated with a CQD thin layer and a gold electrode. XPS was utilized to characterize the chemical composition of the CQDs, the concentrations of nitrogen and oxygen eteroatoms as well as the bonds formed by these elements with C atoms. UV-Vis spectra show two features that can be associated with the presence of the π → π* and σ → π* absorption bands due to aromatic sp^2^-hybridized C atoms and with the n → π* transition, reflecting the presence of C = O, C = N bonds. By increasing the N concentration in the CQDs, a redshift in the photoluminescence spectrum was observed. UPS with He I radiation at 21.2 eV was utilized to determine the band diagram of the CQDs for a specific doping level (12.7% from XPS). The authors determined the spectral window defined by the valence band maximum and the cutoff energy. The valence band maximum defines the CQDs’ HOMO position. The cutoff energy corresponds to the upper edge of the He I valence band. From these values, it is possible to estimate the position of the valence band E_VB_ with respect to the vacuum, E_VB_ = −21.2 + (E_cutoff_ − VB_max_) = −6.6 eV. The minimum of the conduction band was readily calculated: CB_min_ = E_VB_ + E_g_ = −3.9 eV, where the bandgap E_g_ = 2.7 eV was obtained from absorption spectra (Tauc plot). The VB_max_ and CB_min_ of the CQDs were compared with those of the TiO_2_ placed at −7.4 eV and −4.2 eV, respectively. The different band energies of the TiO_2_ and CQDs led to a higher efficiency in separating the excitons and avoiding recombination to an open circuit voltage = 1.8 V and an efficiency of 0.8%. 

The presence of oxygen functionalities facilitates the dispersivity of the CQDs in polar solvents and adds photoluminescence (PL) properties. These kinds of CQDs display a rather wide photoluminescence spectrum which extends from 400 to 600 nm. The maximum of the photoluminescence (PL) is located in the blue region at ~460 nm and presents an exponential decay until ~600 nm, where it is almost null. Different emission properties may be obtained by treating the CQDs with dimethylformamide to introduce nitrogen functional groups, with sodium hydrosulfide to graft sulphur functional groups and with sodium selenide to bind selenium [234]. The three different CQDs showed tunable PL and an improved quantum yield and lifetime with respect to conventional CQDs. Other CQD synthesis methods include arc discharge [243], laser ablation and electrochemical synthesis. High-energy laser pulses were used to vaporize a carbon target in the presence of water vapors, leading to the crystallization of carbon NP [243,244]. It is possible to generate CQDs with the desired chemistry by selecting a proper organic solvent during the laser irradiation [245]. This resulted in a tunable PL whose origin was attributed to carboxylate ligands on the CQD surface. CQDs were produced by the electrochemical oxidation of a graphitic electrode in an aqueous solution [246]. Similarly, graphite was exfoliated in a NaOH/EtOH electrolyte using an electrochemical process, resulting in the production of CQDs that were 1–4 nm in size [247]. The large-scale production of CQDs was electrochemically carried out in simple water using a carbon electrode without any other additive [248]. This process led to the production of a water suspension of highly crystalline and pure CQDs, displaying up- down-conversion photoluminescence.

Because the PL properties of the CQDs depend on the particulate size and surface composition, generally, characterization is carried out using high-resolution microscopy such as transmission electron microscopy (TEM), scanning electron microscopy (SEM) or atomic force microscopy (AFM). As for the surface composition, it may be obtained by titration by using NMR. XPS is broadly utilized to reveal the surface composition and estimate the concentration of the different elements. In the following, we will give only some examples among the wide literature. The authors of [249] synthesized nitrogen-doped CQDs to detect oxytetracycline in environmental samples. The CQDs were characterized by UV-Vis absorption spectra and fluorescence for the optical properties. Atomic force microscopy (AFM) and high-resolution transmission electron microscopy were used to estimate the morphological properties, while FTIR and XPS were used to check the chemical composition. The characterization proved the efficient nitrogen doping and a stable fluorescence performance. Finally, the authors demonstrated that the N-CQDs were able to efficiently detect oxytetracycline in various reaction conditions. Another example is the work [250], where the authors synthesized N-doped CQDs to detect Cr(IV) ions through a fluorescence “turn-off” mechanism. Additionally, in this case, FTIR spectroscopy, TEM and UV-Vis spectrometry were used to characterize the N-CQDs. XPS certified the presence of the nitrogen doping. The N 1s core line shows the components at 398.7, 400.4 and 402.3 eV, which were assigned to the C-N, -NH and -NH_2_ chemical bonds, respectively. As for the Cr(IV) detection, the N-CQDs displayed a wide linear range from 0 to 60 μmol/L, with a low limit of detection of 348.18 μmol/L.

**Figure 20:**
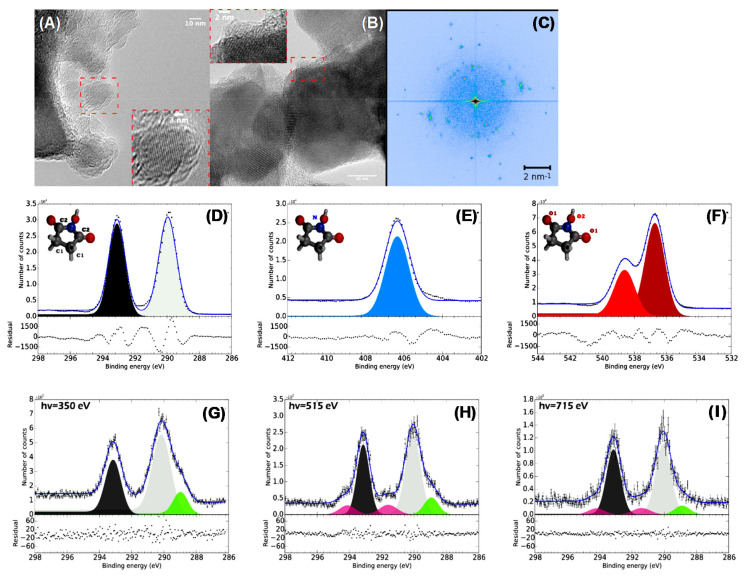
(**A**) HRTEM image of the CQDs suspension, with red squares indicating regions with crystal planes, and (**B**) HRTEM of the CQDs beyond the interaction point, with the synchrotron radiation. (**C**) Two-dimensional fast Fourier transformation of the whole image (**B**). Below XPS spectra recorded at a photon energy of 1486.7 eV for (**D**) C 1s, (**E**) N 1s and (**F**) O 1s of a solid N-hydroxysuccinimide (NHS) precursor deposited on an indium substrate. (**G**–**I**) C 1s core level photoelectron spectra of CQDs, recorded at various photon energies and corresponding to probing depths of (**D**) 1.16 ± 0.34 nm, (**E**) 1.54 ± 0.52 nm and (**F**) 3.44 ± 1.5 nm. Gray and black components are assigned to the C1 and C2 sites, respectively, while the green one is attributed to a silicon carbide bond and the pink ones are assigned to residual gas-phase NHS. Reproduced with permission from [254].

In [251], the authors utilized a large-scale synthesis method to derive CQDs from glucose, cellulose, hemicellulose and chitosan through mild oxidation in a NaOH/H_2_O_2_ solution. XPS coupled with FTIR was used to determine the chemical composition of the CQDs. XPS revealed the presence of hydroxyl, epoxy, carbonyl and carboxyl groups on the surface of the CQDs. Thanks to these functionalities, the CQDs display an excellent quantum yield (QY) as high as 22.67% when they are synthesized using glucose. In another work [252], the authors were able to explain the PL properties of CQDs using XPS and pH-dependent fluorescence titration. They discovered that the PL depends on the presence of protonable oxygen functionalities such as phenolic −OH and −COOH. The presence of these groups explains why there is not a correlation of the PL on the CQDs’ size and the absence of the long-lived optical excitation of the core. Rather, the CQDs depend on the number of oxygen-containing defects defining the emission wavelength, with more reddish emissions increasing the defect density. CQDs are commonly used as fluorescent tags for their stable PL and the absence of photobleaching. In [253], XPS was utilized to detect the composition and interaction of CQDs with TiO_2_ to enhance the light absorption and then the TiO_2_ photocatalitic properties. XPS revealed that there were not direct bonds between CQDs and TiO_2_. Rather, there are weak electrostatic interactions which preserve both the TiO_2_ and the CQDs’ electronic structure. This allows the CQDs to convert visible light in UV photons readily absorbed by TiO_2_, enhancing the degradation of methyl orange. Interestingly, a compositional depth profile of CQDs was performed using X-ray photons of different energies produced by a synchrotron radiation beamline [254]. The CQDs were produced by the pyrolysis of N-hydroxysuccinimide (NHS). Interestingly, the analysis was performed on free-standing NP by the generation of a CQD aerosol beam propagating under vacuum conditions. Figure 20A reports an example of HRTEM images of quantum dots. The crystalline structure of the CQDs is highlighted in the red-squared region. Figure 20B shows the cumulative effect of the generation and transfer, focusing the CQDs aerosol beam on the region irradiated by the synchrotron radiation. The image shows nearly spherical CQDs, with a particle dimension ranging from 10 to 20 nm. Figure 20C represents the FFT of Figure 20B to highlight the crystalline structure of the CQDs. The functional group molecule is shown as the inset of Figure 20D–F and was characterized by XPS. The analysis enlightens the carbon, nitrogen and oxygen components. C 1s is composed of two main components assigned to the CH_x_ and N-C=O bonds (denoted with C1 and C2), respectively, at 290 and 293 eV. After the pyrolyzation of the NHS, the authors exploited the different sampling depths associated with 350 eV, 515 eV and 715 eV photons to characterize the CQDs, which, for instance, vary from 1.16nm to 1.54 nm and 3.44 nm. As shown in Figure 20D–F, increasing the photon energy increases the sampling depth, leading to changes in the C 1s core line. The higher the sampling depth, the higher the graphitic components of the CQDs, while the oxygen and nitrogen functionalities appear to be more abundant on the external parts of the CQDs. The authors concluded that the composition of the CQD surface is similar to that of the NHS precursor.

### 4.6. Carbon Nanofibers

Let us turn our attention to carbon nanofibers (CNF). 

Carbon fibers (CNF) are highly anisotropic fibrous materials possessing a graphitic structure with strong crystallite covalent bonds. These fibers display a two-dimensional long-range order of carbon atoms, forming stakes of planar hexagonal networks, while no regularity is present in the third dimension. CNFs are known for their prominent mechanical properties, which depend on the synthesis process and the precursor used. Their tensile strengths range from 5.65 GPa to 1.5 GPa, and their specific modulus ranges from 106 GPa to 407 GPa [255], exceeding the strength and modulus of steel—2.39 GPa and 26.6 GPa, respectively [256]. CNFs are broadly utilized as reinforced and conductive elements in polymers and composites in general [257]. Table 2 summarizes some of the properties of CNFs.

CNFs are obtained by pyrolyzing organic materials such as rayon, polyacrylonitrile and pitch [259]. CNFs can also be produced by CVD processes using an appropriate metal catalyzer (for example, Fe, Co, Ni, Cr, Va, etc.) and hot filament-assisted sputtering [260]. In a CVD process, the growth of the CNF is carried out in a flow furnace at an atmospheric pressure. The gaseous precursor (for example, acetylene) is introduced in the reactor chamber, where it decomposes on a catalyzer surface at temperatures from 500 to 1000 °C [261,262]. CNFs can be grown in a vapor atmosphere using a “floating catalyst” carried in a continuous flow reactor by a gaseous precursor [263]. A CVD process my result in the production of two kinds of CNF, namely, the cone- [264] and platelet-staked [265] CNF. In both cases, the piling of cone-shaped CNFs or platelets leads to the formation of elongated structures, namely, the nanofibers. In this process, the precursors such as CO, H_2_, CH_4_, ethane and ethyne decompose and dissolve on the catalyzer surface and grow as graphitic carbon [266]. The crystalline structure of the catalytic nanosized metal particles determines the shape of the CNF. The latter is also produced in plasma generated by an arc discharge [267,268]. Electrospinning is another method to synthesize the CNF [269]. Different polymers such as polystyrene (PS), polyvinylchloride (PVC), polymethylmetacrylate (PMMA), polyacrylonitrile (PAN), polyvinylalcohol (PVA), polyvinylidenfluoride (PVDF), etc. can be used to produce woven fibers. Then, a carbonization treatment is applied to obtain the CNFs. The thermal treatment is performed at a temperature typically ranging between 600 and 1000 °C. The preparation and applications of CNFs produced by electrospinning are reviewed in [270]. More recently, microwaves are used to assist the pyrolysis of biomass [271,272]. For example, ligno-cellulosic biomass is a suitable precursor for the production of CNFs [273]. In this process, microwaves generate high-energy plasma, resulting in the pyrolysis of the biomass precursor and the generation of CNFs [274]. 

Concerning characterization, XPS is mainly utilized to detect the surface chemistry. Effects related to the quantum confinement in CNFs are absent because of the overly large system size. One of the more diffuse applications of CNFs is the use in composite materials to enhance the mechanical properties. In this respect, a good integration of the CNF in the host matrix is required. For this aim, the surface chemistry plays a fundamental role, since it determines the chemical interaction between the CNF and the hosting material. XPS is commonly used to determine the surface composition of the CNF—for example, in [275]. In this work, XPS was used to detect the effect of progressive HNO_3_ acid treatment on the CNF. The results show that the acid treatment transforms the initially heterogeneous surface of the pristine CNF in a more homogeneous composition. The acid induces oxidation with the generation of carbonyl and carboxyl groups. In another work, XPS was used to study the effect of a plasma treatment on the surface of a CNF. The authors found that plasma induced the grafting of oxygen and nitrogen functionalities. The presence of polar oxygen radicals on the surface resulted in an increase in the surface energy of the CNFs, thus enhancing their adhesion properties. In [276], CNFs were produced by the pyrolyzation of electrospinned PAN. The CNFs were then decorated with Fe_3_O_4_ NPs to fabricate the anodes for Li ion batteries. CNFs can also be successfully utilized in the fabrication of supercapacitors [277]. The surface composition was determined by XPS. The decorated CNF composite exhibited a high specific capacity and a better cycle stability when compared to that of the simple CNF electrodes. SEM, HRTEM, XRD, FTIR, XPS, TGA, Raman, electrochemical tests and cyclic voltammetry impedance measurements were utilized to characterize the structural, chemical and electric properties of the PAN/PMMA CNFs. As for the surface composition, XPS was utilized to detect and quantify the C, O and N at the CNF surface. In particular, the N 1s peak was deconvoluted in pyridinic-, pyrrolic-, graphitic- and oxidized-nitrogen components. The authors found that the O and N surface functionalities significantly enhanced the total capacitance. The porous CNF mats prepared with a PAN/PMMA = 7:3 showed a specific capacitance of 140.8 F g^−1^ and very good stability, with a decrease of only 4.6% after 10,000 charge/discharge cycles. Another area where a high specific surface area is beneficial is the electrocatalysis. Recent studies on the development of efficient electrocatalysts focused on the synergistic effect of coupling transition and CNFs, resulting in a large specific surface area, a short mass diffusion distance and a fast electron transport [278]. In this work, CNFs were produced by electrospinning a solution containing polyvinylpyrrolidone, N-Dimethylformamide, Co(NO_3_)_2_ and Fe(NO_3_)_3_. The polymer nanofibers were then stabilized at 250 °C for 3 h in air and calcinated at 600 °C in an N_2_ atmosphere for 3 h. The CNFs were then characterized using SEM, HRTEM, XRD and cyclic voltammetry. The chemical compositions and valence states of the CoFe2O4@N-CNFs were studied using XPS, where the main constituents—C, N, O, Fe and Co—and their chemical state were detected. In particular, the presence of the shake-up satellite peak of iron coupled with the BE values indicate a Fe^3+^ oxidation state. Similarly, for Co, the presence of the shake-up feature and the BE of the 2p components suggest an oxidation state Co^2+^ for cobalt. Carbon is present with the main graphitic component and C-O bonds introduced by the covalent linkage with the CoFe_2_O_4_ NPs. N 1s displays the presence multiple components derived from pyridinic, pyrrolic, graphitic N and oxidized N. The presence of N in the CNF has two advantageous effects: increasing the electrical conductivity and introducing defects which behave as active sites for the electrocatalysis, increasing the reaction kinetics. Another important application of the CNFs is CO_2_ adsorption. Microporous carbon nanofibers were produced by electrospinning PAN, followed by stabilization and carbonization [279]. EDX, FTIR and XPS were utilized to characterize the surface chemistry of the CNF. In particular, the N 1s features shifted to a higher BE, and the N/C ratio decreased with the increase in the activation temperature and time. This allowed for the selection of the best activation conditions to increase the formation of ultramicropores while maintaining the N functionalities and reducing its evolution. As a result, the higher the N functional group abundance was, the higher the CO_2_ adsorption was.

In another interesting work [280], the authors studied the effect of polyacetonitrile CNF surface treatment. Different degrees of oxidative electrochemical surface treatments were applied to the CNF. Inverse gas chromatography and XPS were utilized to determine the chemical species induced on the CNF surface. The authors described the variation of the acidity and basicity parameters, K_A_ and K_D_, which were strongly correlated to the oxygen and nitrogen surface compositions. An acid-base interaction is induced by a perturbation between the accepting and donating orbitals. This information is of paramount importance for coupling the CNF with the external matrix and in the modulation of their electrical properties. As an example, the degree of acidity/basicity influences the electrons interacting with two different outer electronic orbitals. This is the case for the π–π* type interaction, where one orbital has electrons to donate and the other has unfilled states behaving as acceptors. 

In another study [281], the C 1s high-resolution core line revealed neglectable heteroatoms (O/C ratio is 0.019), confirming the purity of the sp^2^ graphitic phases in the CNF. Optical measurements exhibit an absorption peak located in the UV region at 270 nm, while the emission spectra display a peak at 420 nm. However, this emission line cannot be explained by the σ → π* transition due to the absence of C-O bonds in the CNF. A possible explanation supported, by theoretical calculations, a broadening of the π–π* transition band, while the adsorption at 240 nm may be due to the overlap between the σ and π bands, which appear at 9.6 eV and 2.3 eV in XPS VB.

**Figure 21:**
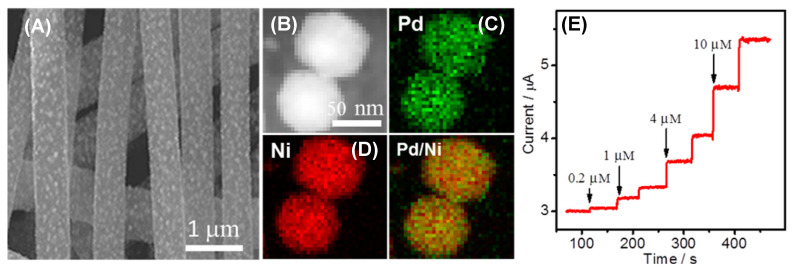
(**A**) Typical SEM images of Pd−Ni/CNF nanocomposites with Pd_30_Ni_70_/CNF molar feed ratio at Tc = 850 °C. (**B**) STEM image and EDX maps of Ni (**C**), Pd (**D**) and their superposition. (**E**) The Current responses of the Pd_30_Ni_70_ CFP electrode upon the successive addition of glucose solutions with varying concentrations; applied potential: 0.40 V vs. Ag/AgCl. Reprinted with permission from [283].

CNFs are useful also in the biomedical area. For example, they can be utilized in wound dressing materials, in bone tissue regeneration and in biosensing [282]. In all these cases, the control of the surface composition is mandatory to ensure perfect biocompatibility and functionality. As an example, Pd/Ni-decorated CNFs produced from the electrospinning of PAN carbonized at T_c_ = 850 °C were utilized to detect sugars. In Figure 21A, [283] an SEM image of the decorated CNF shows the high density of Pd/Ni clusters on the fibers’ surfaces. In Figure 21B STEM image and correspondent Pd and Ni EDX maps in Figure 21C, Figure 21D are utilized to verify the cluster composition. In the analyzed region, the Pd and Ni are localized in the same region, demonstrating the atomic mixture of Pd and Ni without phase segregation. Finally, Figure 21E displays the current responses of a Pd_30_Ni_70_CNF electrode upon the increasing concentration of glucose in the solution with applied potential: 0.40 V vs. Ag/AgCl. Higher currents are obtained with increasing glucose concentrations. Raising the carbonization temperature increases the concentration of the Pd/Ni on the CNF surface, resulting in the higher sensitivity of the electrode. In particular, the sensor showed a very low limit of sugar detection of 7–20 nM, with a linear response in the 0.03–800 μM range and a high resistance to surface fouling. Additional information concerning the synthesis and the applications of CNFs may be found in [284,285]. 

### 4.7. Conclusions

Today, nanostructures are playing a crucial role in the development of many important commercial applications. The success in using these objects is related the properties arising from the reduction of the system size to the nanometric scale, e.g., higher chemical reactivity, optical emission, superior mechanical and electrical properties and higher interaction with biological systems. Critical is the characterization of these properties to ensure that these nanoobjects can be successfully integrated in devices where they must provide specific functionalities. XPS is a powerful technique offering a rich list of information related to the chemical composition, the electronic configuration and the physical structure. This work is dedicated to the description of the potentialities of XPS for the characterization of different carbon-based materials with the aim of showing what kind of information can be obtained and how XPS instruments can be used to characterize C-nanostructures.

Because carbon is a multiform element, it can be organized in a rich variety of allotropes starting from the two prototypes, diamond and graphite, to the list of nanostructures such as graphene, quantum dots, nanotubes, fibers, fullerenes and nanodiamonds. Probing the electronic structure of materials, photoelectron spectroscopies can supply information regarding the chemical composition and how elements are bonded together. At the same time, the spectral features are influenced by the presence of the regular organization of atoms in crystalline lattices or amorphous phases or the presence of quantum confinement induced by nanodimensions. On the other hand, the electronic configuration of atoms directly influences their optical transitions. Then, the investigation of the valence bands (XPS and UPS valence bands) may help to shed light on the materials and the photoluminescence properties. The analysis of valence states coupled with the information derived from the C KLL Auger and core line spectra help in determining the presence of different hybridization states, e.g., graphitic/diamond or diamond-like phases.

Much time has gone by since the discovery of the photoelectric effect by Einstein and the subsequent developments by Siegbahn and co-workers. Much work is dedicated to obtaining brighter analyzers for increasing the possibility of obtaining an even more detailed picture of how atoms are bonded together, their magnetic state and structural maps in nanosized structures. Although not presented in the present work, spin- and time-resolved measurements and ambient pressure analysis are crucial for providing a description and understanding of the behavior of complex systems. Other areas of current research are the development of new, more intense X-ray sources and hard and tunable photon sources (synchrotron radiation), enabling higher energy and angular (k-vector) resolutions. These new improvements will be crucial for the characterization of future advanced functional nanomaterials. The increasing demand of high performing systems in energy, chemistry and biotechnology and the environmental, biomedical and life sciences is connected to the development of high-tech materials. Photoelectron spectroscopies are likely to address the need for an even deeper understanding of the properties of materials when organized in nanostructures. In this context, photoelectron spectroscopies are a very powerful tool assisting in the development of novel and original solutions.

## Figures and Tables

**Table 1:** Comparison of the performances of conventional XPS instruments, UPS (He I, HeII radiation or synchrotron radiation) and Hard X-ray Photoelectron Spectroscopy (HAXPS).

	XPS		UPS: HeI	HeII	UV Synchrotron	HAXPES
**Energy (eV)**	1486.6 (Al Kα)	1253.6 (Mg Kα)	20.2	40.8	10–10^2^	10^2^–10^4^ eV
**Δλ (eV)**	0.25	0.7	0.003	0.017	10^−4^	~0.01 eV
**Energy resolution (eV)**	0.28	0.9–1	<0.1		0.01	0.6
**Lateral resolution (μm)**	3		2		-	Submicron
**Sampling depth (nm)**	3–10		0–1.5		0–2	0–20
**Angle-resolved**	Yes		Yes		Yes	Yes
**X diffraction**	//		//		Yes	Yes
**Characterization**						
**Electronic structure**	XPS VB and DOS		UV DOS		UV DOS	XPS VB and DOS
**Quantum confinement**	BE shift of core lines		Work function shift, energy gaps		Work function shift, energy gaps	BE shift of core lines
**Structural information**	NP size estimation		Presence of curvature		Presence of curvature	NP size estimation
**Chemical information**	Chemical shift		VB orbital bands		VB orbital bands	Chemical shift
**Different hybridization**	Core lines, VB and KLL Auger spectra		VB analysis		VB analysis	Core lines, VB and KLL Auger spectra
**Band structure**	Angle-resolved VB		Angle-resolved VB		Angle-resolved VB	Angle-resolved VB
**Microscopy**	-		-		-	Submicron structure

Note that Δλ represents the excitation linewidth, while the energy resolution refers to the instrumental resolving power. Data related to XPS instruments are taken from ref. [44,45]. Data of UPS He lamps are taken from ref. [20,46,47]. Data related to synchrotron radiation are derived from ref. [11,48,49].

**Table 2:** Typical properties of Vapor-grown CNFs (VGCNF), SWCNTs, MWCNTs and CNFs (*).

Property	VGCNF	SWCNT	MWCNT	CNF
Diameter (nm)	50–200	0.6–0.8	5–50	7300
Aspect ratio	250–2000	100–10,000	100–10,000	440
Density (g/cm^3^)	2	~1.3	~1.75	1.74
Thermal conductivity (W/mK)	1950	3000–6000	3000–6000	20
Electrical resistivity (Ω/cm)	1 × 10^−4^	1 × 10^−3^–1 × 10^−4^	2 × 10^−3^–1 × 10^−4^	1.7 × 10^−3^
Tensile strength (GPa)	2.92	50–500	10–60	3.8
Tensile modulus (GPa)	240	1500	1000	227

(*) (data obtained with permission from ref. [258]).

## Data Availability

Not applicable.

## References

[1] Mohan V.B., Lau K.-T., Hui D., Bhattacharyya B. (2018). Graphene-based materials and their composites: A review on production, applications and product limitations. Compos. Part B Eng..

[2] Backes C., Abdelkader A.M., Alonso C., Andrieux-Ledier A., Arenal R., Azpeitia J., Balakrishnan N., Banszerus L., Barjon J., Bartali R. (2020). Production and processing of graphene and related materials. 2D Mater..

[3] Mochalin V.N., Shenderova O., Ho D., Gogotsi Y. (2011). The properties and applications of nanodiamonds. Nat. Nanotechnol..

[4] Aharonovich I., Neu E. (2014). Diamond Nanophotonics. Adv. Opt. Mater..

[5] Georgakilas V., Perman J.A., Tucek J., Zboril R. (2015). Broad Family of Carbon Nanoallotropes: Classification, Chemistry, and Applications of Fullerenes, Carbon Dots, Nanotubes, Graphene, Nanodiamonds, and Combined Superstructures. Chem. Rev..

[6] Jariwala D., Sangwan V.K., Lauhon L.J., Marks T.J., Hersam M.C. (2013). Carbon nanomaterials for electronics, optoelectronics, photovoltaics, and sensing. Chem. Soc. Rev..

[7] Ruppender H.J., Grunze M., Kong C.W., Wilmers M. (1990). In situ X-ray photoelectron spectroscopy of surfaces at pressures up to 1 mbar. Surf. Interface Anal..

[8] Trotochaud L., Head A.R., Karslglu O., Kyhl L., Bluhm H. (2017). Ambient pressure XPS Practical considerations and experimental frontiers. J. Phys. Condens. Matter.

[9] Moulde J.F. (2019). The Impact of the Scanning XPS Microprobe on Industrial Applications of X-ray Photoelectron Spectroscopy. J. Electron Spectrosc. Relat. Phenom..

[10] Roberts A.J., Moffitt C.E. (2019). Trends in XPS instrumentation for industrial surface analysis and materials characterization. J. Electron Spectrosc. Relat. Phenom..

[11] Mino L., Borfecchia E., Segura-Ruiz J., Giannini C., Martinez-Criado G., Lamberti C. (2018). Materials characterization by synchrotron x-ray microprobes and nanoprobes. Rev. Mod. Phys..

[12] Egelhoff W.F. (1987). Core-level binding-energy shifts at surfaces and in solids. Surf. Sci. Rep..

[13] Schattke W., van Hove M.A. (2003). Solid-State Photoemission and Related Methods: Theory and Experiment.

[14] Miron C., Morin P. (2011). Handbook of High-Resolution Spectroscopy.

[15] Susi T., Pichler T., Ayala P. (2015). X-ray photoelectron spectroscopy of graphitic carbon nanomaterials doped with heteroatoms. Beilstein J. Nanotechnol..

[16] Weiland C., Rumaniz A.K., Pianetta P., Woicik J.C. (2016). Recent applications of hard X-ray photoelectron spectroscopy. J. Vac. Sci. Technol. A.

[17] Nordling C., Sokolowski E., Siegbahn K. (1957). Precision Method for Obtaining Absolute Values of Atomic Binding Energies. Phys. Rev..

[18] Fadley C.S. (2009). X-ray photoelectron spectroscopy: From origins to future directions. Nucl. Instrum. Methods Phys. Res. A.

[19] Hill J.P., Kaufmann E.N. (2003). Ultraviolet Photoelectron Spectroscopy. Characterization of Materials.

[20] Rabalais J.W. (1977). Principles of Ultraviolet Photoelectron Spectroscopy.

[21] Powell C.J., Seah M.P. (1990). Precision, accuracy, and uncertainty in quantitative surface analyses by Auger-electron spectroscopy and X-ray photoelectron spectroscopy. J. Vac. Sci. Technol. A.

[22] Tilinin I.S., Jablonski A., Werner W.S.M. (1996). Quantitative surface analysis by Auger and X-ray photoelectron spectroscopy. Prog. Surf. Sci..

[23] Seah M.P., Briggs D., Seah M.P. (1990). Quantification of AES and XPS. Practical Surface Analysis Vol. 1 Auger and X-ray Photoelectron Spectroscopy.

[24] Fadley C.S. (2010). X-ray photoelectron spectroscopy: Progress and perspectives. J. Electron Spectrosc. Relat. Phenom..

[25] Reilman R.F., Msezane A., Manson S.T. (1976). Relative intensities in photoelectron spectroscopy of atoms and molecules. J. Electron Spectrosc. Relat. Phenom..

[26] Scofield J.H. (1976). Hartree-Slater subshell photoionization cross-sections at 1254 and 1487 eV. J. Electron Spectrosc. Relat. Phenom..

[27] Wagner C.D., Davis L.E., Zeller M.V., Taylor J.A., Raymond R.H., Gale L.H. (1981). Empirical atomic sensitivity factors for quantitative analysis by electron spectroscopy for chemical analysis. Surf. Interface Anal..

[28] Brundle C.R., Crist B.V. (2020). X-ray photoelectron spectroscopy: A perspective on quantitation accuracy for composition analysis of homogeneous materials. J. Vac. Sci. Technol. A.

[29] Briggs D. (1998). Surface Analysis of Polymers by XPS and Static SIMS.

[30] Seah M.P., Dench W.A. (1979). Quantitative electron spectroscopy of surfaces: A standard data base for electron inelastic mean free paths in solids. Surf. Interface Anal..

[31] Mason M.G. (1983). Electronic structure of supported small metal clusters. Phys. Rev. B.

[32] Watson R.E., Perlman L. (1975). X-ray photoelectron spectroscopy application to metals and alloys. Structure and Bonding.

[33] Wertheim G.K., DiCenzo S.B., Youngquist S.E. (1983). Unit Charge on Supported Gold Clusters in Photoemission Final State. Phys. Rev. Lett..

[34] Wertheim G.K., DiCenzo S.B. (1988). Cluster growth and core-electron binding energies in supported metal clusters. Phys. Rev. B.

[35] Calliari L., Speranza G., Minati L., Micheli V., Baranov A., Fanchenko S. (2008). Composition and structure of a-C: Au nanocomposites obtained by physical vapour deposition. Appl. Surf. Sci..

[36] Videnovic I., Oelhafen P. (2005). Photoelectron spectroscopy study of metallic nanocluster arrangement at the surface of reactively sputtered amorphous hydrogenated carbon. J. Appl. Phys..

[37] Takahiro K., Oizumi S., Terai A., Kawatsura K., Tsuchiya B., Nagata S., Yamamoto S., Naramoto H., Narumi K., Sasase M. (2006). Core level and valence band photoemission spectra of Au clusters embedded in carbon. J. Appl. Phys..

[38] Minati L., Speranza G., Calliari L., Micheli V., Baranov A., Fanchenko S. (2008). The Influence of Metal Nanoparticle Size Distribution in Photoelectron Spectroscopy. J. Phys. Chem. A.

[39] Zorn G., Dave S.R., Gao X., Castner D.G. (2011). Method for Determining the Elemental Composition and Distribution in Semiconductor Core-Shell Quantum Dots. Anal. Chem..

[40] Kono S., Kageura T., Hayashi Y., Ri S.-G., Teraji T., Takeuchi D., Ogura M., Kodama H., Sawabe A., Inaba M. (2019). Carbon 1s X-ray photoelectron spectra of realistic samples of hydrogen-terminated and oxygen-terminated CVD diamond (111) and (001). Diam. Relat. Mater..

[41] Powell C.J., Jackson A. (2000). NIST Electron Inelastic-Mean-Free-Path Database.

[42] Brzhezinskaya M.M., Muradyan V.E., Vinogradov N.A., Preobrajenski A.B., Gudat W., Vinogradov A.S. (2009). Electronic structure of fluorinated multiwalled carbon nanotubes studied using X-ray absorption and photoelectron spectroscopy. Phys. Rev. B.

[43] Baer D.R. (2020). Guide to making XPS measurements on nanoparticles. J. Vac. Sci. Technol. A.

[44] Briggs D., Seah M.P. (1990). Practical Surface Analysis.

[45] Briggs D., Grant J. (2003). Surface Analysis by Auger and X-ray Photoelectron Spectroscopy.

[46] Briggs D. (1977). Handbook of X-ray and Ultraviolet Photoelectron Spectroscopy.

[47] Hufner S. (2003). Photoelectron Spectroscopy: Principles and Applications.

[48] Kalha C., Fernando N.K., Bhatt P., Johansson F.O.L., Lindblad A., Rensmo H., Zendejas-Medina L., Lindblad R., Sio S., Jeurgens L.P.H. (2021). Hard X-ray photoelectron spectroscopy: A snapshot of the state-of-the-art in 2020. J. Phys. Condens. Matter.

[49] Weinhardt L., Steininger R., Kreikemeyer-Lorenzo D., Mangold S., Hauschild D., Batchelor D., Spangenberg T., Heske C. (2021). X-SPEC: A 70 eV to 15 keV undulator beamline for X-ray and electron spectroscopies. J. Synchrotron Radiat..

[50] Baer D.R., Engelhard M.H. (2010). XPS analysis of nanostructured materials and biological surfaces. J. Electron Spectrosc. Relat. Phenom..

[51] Doniach S., Sunjic P. (1970). Many-electron singularity in X-ray photoemission and X-ray line spectra from metals. J. Phys. C Solid State Phys..

[52] Speranza G., Minati L. (2006). The surface and bulk core lines in crystalline and disordered polycrystalline graphite. Surf. Sci..

[53] Speranza G., Miinati L., Anderle M. (2007). The C1s core line in irradiated graphite. J. Appl. Phys..

[54] Prince K.C., Ulrych I., Peloi M., Ressel B., Chab V., Crotti C., Comicioli C. (2000). Core-level photoemission from graphite. Phys. Rev. B.

[55] Lesiak B., Zemek J., Houdkova J., Kromka A., Jozwik A. (2010). Electron spectra Line shape analysis of Highly oriented Pyrolytic Graphite and Nanocrystalline diamond. Anal. Sci..

[56] Balasubramanian T., Andersen J.N., Walladen L. (2001). Surface-bulk core-level splitting in graphite. Phys. Rev. B.

[57] Smith R.A.P., Armstrong C.W., Smith G.C., Weightman P. (2002). Observation of a surface chemical shift in carbon 1s core-level photoemission from highly oriented pyrolytic graphite. Phys. Rev. B.

[58] Yang D.-Q., Sacher E. (2006). Carbon 1s X-ray Photoemission Line Shape Analysis of Highly Oriented Pyrolytic Graphite: The Influence of Structural Damage on Peak Asymmetry. Langmuir.

[59] Geim A.K., Novoselov K.S. (2007). The rise of graphene. Nat. Mater..

[60] Lee C., Wei X., Kysar J.W., Hone J. (2008). Measurement of the Elastic Properties and Intrinsic Strength of Monolayer Graphene. Science.

[61] Bolotin K.I., Sikes K.J., Jiang Z., Klima M., Fudenberg G., Hone J., Kim P., Stormer H.L. (2008). Ultrahigh electron mobility in suspended graphene. Solid State Commun..

[62] Züttel A., Sudan P., Mauron P., Wenger P. (2004). Model for the hydrogen adsorption on carbon nanostructures. Appl. Phys. A.

[63] Nair R.R., Blake P., Grigorenko A.N., Novoselov K.S., Booth T.J., Stauber T., Peres N.M.R., Geim A.K. (2008). Fine Structure Constant Defines Visual Transparency of Graphene. Science.

[64] Huang H., Chen S., Wee A.T.S., Chen W. (2014). Chap 1-Epitaxial growth of graphene on silicon carbide (SiC). Graphene-Properties, Preparation, Characterization and Devices.

[65] Wang J.-B., Ren Z., Hou Y., Yan X.-L., Liu P.-Z., Hua Z., Zhang H.X., Guo J.J. (2020). A review of graphene synthesis at low temperatures by CVD methods. New Carbon Mater..

[66] Novoselov K.S., Geim A.K., Morozov S.V., Jiang D., Zhang Y., Dubonos S.V., Grigorieva I.V., Firsov A.A. (2004). Electric Field Effect in Atomically Thin Carbon Films. Science.

[67] Hernandez Y., Nicolosi V., Lotya M., Blighe F.M., Sun Z., De S., McGovern I.T., Holland B., Byrne M., Gun’Ko Y.K. (2008). High-yield production of graphene by liquid-phase exfoliation of graphite. Nat. Nanotechnol..

[68] Knieke C., Berger A., Voigt M., Taylor R.N.K., Röhrl J., Peukert W. (2010). Scalable production of graphene sheets by mechanical delamination. Carbon.

[69] Lv Y., Yu L., Jiang C., Chen S., Nie Z. (2014). Synthesis of graphene nanosheet powder with layer number control via a soluble salt-assisted route. RSC Adv..

[70] Hummers W.B., Offeman R.E. (1958). Preparation of graphitic oxide. J. Am. Chem. Soc..

[71] Guex L.G., Sacchi B., Peuvot K.F., Andersson R.L., Pourrahimi A.M., Strom V., Farris S., Olsson R.T. (2017). Experimental review: Chemical reduction of graphene oxide (GO) to reduced graphene oxide (rGO) by aqueous chemistry. Nanoscale.

[72] Saleem H., Haneef M., Abbasi H.Y. (2018). Synthesis route of reduced graphene oxide via thermal reduction of chemically exfoliated graphene oxide. Mater. Chem. Phys..

[73] Uz M., Jackson K., Donta M.S., Jung J., Lentner M.T., Hondred J.A., Claussen J.C., Mallapragada S.K. (2019). Fabrication of High-resolution Graphene-based Flexible Electronics via Polymer Casting. Sci. Rep..

[74] Deng D., Novoselov K.S., Fu Q., Zheng N., Tian Z., Bao X. (2016). Catalysis with two-dimensional materials and their heterostructures. Nat. Nanotechnol..

[75] Nag A., Mitra A., Chandra M. (2018). Graphene and its sensor-based applications: A review. Sens. Actuators A.

[76] Peña-Bahamond J., Nguyen H.N., Fanourakis S.K., Rodrigues D.F. (2018). Recent advances in graphene-based biosensor technology with applications in life sciences. J. Nanobiotechnol..

[77] Meyer J.C. (2014). Chapter 5: Transmission electron microscopy (TEM) of graphene. Graphene Properties, Preparation, Characterisation and Devices.

[78] Toma S., Scardamaglia M., Mustonen K., Tripathi M., Mittelberger M., Al-Hada M., Amati M., Sezen H., Zeller P., Larsen A.H. (2018). Intrinsic core level photoemission of suspended monolayer graphene. Phys. Rev. Mater..

[79] Hibino H., Kageshima H., Kotsugi M., Meda F., Guo F.-Z., Watanabe Y. (2009). Dependence of electronic properties of epitaxial few-layer graphene on the number of layers investigated by photoelectron emission microscopy. Phys. Rev. B.

[80] Gupta B., Notarianni M., Mishra N., Shafiei M., Iacopi F., Motta N. (2014). Evolution of epitaxial graphene layers on 3C SiC/Si(111) as a function of annealing temperature in UHV. Carbon.

[81] Niesner D., Fauster T. (2014). Image-potential states and work function of graphene. J. Phys. Condens. Matter.

[82] Kralj M., Pletikosic I., Petrovic M., Pervan P., Milun M., N’Diaye A.T., Busse C., Michely T., Fujii J., Vobornik I. (2011). Graphene on Ir(111) characterized by angle-resolved photoemission. Phys. Rev. B.

[83] Tatti R., Aversa L., Verrucchi R., Cavaliere E., Garberoglio G., Pugno N.M., Speranza G., Taioli S. (2016). Synthesis of single layer graphene on Cu(111) by C60 supersonic molecular beam epitaxy. RSC Adv..

[84] Kraus J., Reichelt R., Gunther S., Gregoratti L., Amati M., Kiskinova M., Yulaev A., Vlassioukd I., Kolmakov A. (2014). Photoelectron spectroscopy of wet and gaseous samples through graphene membranes. Nanoscale.

[85] Xu M., Fujita D., Gao J., Hanagata N. (2010). Auger Electron Spectroscopy: A Rational Method for Determining Thickness of Graphene Films. ACS Nano.

[86] Georgakilas V., Otyepka M., Bourlinos A.B., Chandra V., Kim N., Kemp K.C., Hobza P., Zboril R., Kim K.S. (2012). Functionalization of Graphene: Covalent and Non-Covalent Approaches, Derivatives and Applications. Chem. Rev..

[87] Speranza G. (2019). The Role of Functionalization in the Applications of Carbon Materials: An Overview. C-J. Carbon Res..

[88] Yu W., Sisi L., Haiyan Y., Jie L. (2020). Progress in the functional modification of graphene/graphene oxide: A review. RSC Adv..

[89] Guo Z., Chakraborty S., Monikh F.A., Varsou M.D., Chetwynd A.J., Afantitis A., Lynch I., Zhang P. (2021). Surface Functionalization of Graphene-Based Materials: Biological Behavior, Toxicology, and Safe-by-Design Aspects. Adv. Biol..

[90] Peng C., Zhang X. (2021). Chemical Functionalization of Graphene Nanoplatelets with Hydroxyl, Amino, and Carboxylic Terminal Groups. Chemistry.

[91] Santos E.J.G., Ayuela A., Sanchez-Portal D. (2012). Universal magnetic properties of sp3-type defects in covalently functionalized graphene. New J. Phys..

[92] Elias D.C., Nair R.R., Mohiuddin T.M.G., Morozov S.V., Blake P., Halsall M.P., Ferrari A.C., Boukhvalov D.W., Katsnelson M.I., Geim A.K. (2009). Control of Graphene’s Properties by Reversible Hydrogenation: Evidence for Graphane. Science.

[93] Ngyen P., Berry V. (2012). Graphene Interfaced with Biological Cells: Opportunities and Challenges. J. Phys. Chem. Lett..

[94] Xia J., Chen F., Li J., Tao N. (2009). Measurement of the quantum capacitance of graphene. Nat. Nanotechnol..

[95] Dreyer D.R., Park S., Bielawski C.W., Ruoff R.S. (2010). The chemistry of graphene oxide. Chem. Soc. Rev..

[96] Sreeprasad T.S., Berry V. (2013). How Do the Electrical Properties of Graphene Change with its Functionalization?. Small.

[97] Susi T., Kaukonen M., Havu P., Ljungberg M.P., Ayala P., Kauippen M. (2014). Core level binding energies of functionalized and defective graphene. Beilstein J. Nanotechnol..

[98] Bulusheva L.G., Arkhipov V.E., Popov K.M., Sysoev V.I., Makarova A.A., Okotrub A.V. (2020). Electronic Structure of Nitrogen- and Phosphorus-Doped Graphenes Grown by Chemical Vapor Deposition Method. Materials.

[99] Fates R., Bouridah H., Raskin J.-P. (2019). Probing carrier concentration in gated single, bi- and tri-layer CVD graphene using Raman spectroscopy. Carbon.

[100] Das A., Pisana S., Chakraborty B., Piscanec S., Saha S.K., Waghmare U.V., Novoselov K.S., Krishnamurthy H.R., Geim A.K., Ferrari A.C. (2008). Monitoring dopants by Raman scattering in an electrochemically top-gated graphene transistor. Nat. Nanotechnol..

[101] Scardamaglia M., Toma S., Struzzi C., Snyders R., di Santo G., Petaccia L., Bittencourt C. (2017). Spectroscopic observation of oxygen dissociation on nitrogendoped graphene. Sci. Rep..

[102] Charoenpakdee J., Suntijitrungruang O., Boonchui S. (2020). Chirality effects on an electron transport in single-walled carbon nanotube. Sci. Rep..

[103] Rathinavel S., Priyadharshini K., Panda D. (2021). A review on carbon nanotube: An overview of synthesis, properties, functionalization, characterization, and the application. Mater. Sci. Eng. B.

[104] Eatemadi A., Daraee H., Karimkhanloo H., Kouhi M., Zarghami N., Akbarzadeh A., Abasi M., Henifehpour Y., Joo S.W. (2014). Carbon nanotubes: Properties, synthesis, purification, and medical applications. Nanoscale Res. Lett..

[105] Arora N., Sharma N.N. (2014). Arc discharge synthesis of carbon nanotubes: Comprehensive review. Diam. Relat. Mater..

[106] Prasek J., Drbohlavova J., Chomoucka J., Hubalek J., Jasek O., Adamc V., Kizek R. (2011). Methods for carbon nanotubes synthesis—Review. J. Mater. Chem..

[107] Rahman G., Najaf Z., Mehmood A., Bilal S., ul Haq Ali Shah A., Mian S.A., Ali G. (2019). An Overview of the Recent Progress in the Synthesis and Applications of Carbon Nanotubes. C-J. Carbon Res..

[108] Kumar M., Ando Y. (2010). Chemical vapor deposition of carbon nanotubes: A review on growth mechanism and mass production. J. Nanosci. Nanotechnol..

[109] Hamzah N., Yasin M.F.M., Yusop M.Z.M., Saatac A., Subhae N.A.M. (2017). Rapid production of carbon nanotubes: A review on advancement in growth control and morphology manipulations of flame synthesis. J. Mater. Chem. A.

[110] Yoshida H., Takeda S., Uchiyama T., Kohno H., Homma Y. (2008). Atomic-Scale In-situ Observation of Carbon Nanotube Growth from Solid State Iron Carbide Nanoparticles. Nano Lett..

[111] Adamska M., Narkiewicz U. (2018). Purification of Carbon Nanotubes A Review of Methodology. Nanosci. Nanotechnol. Lett..

[112] Hou P.-X., Liu C., Cheng H.-M. (2008). Purification of carbon nanotubes. Carbon.

[113] Chen P., Wu X., Sun X., Lin J., Tan K.L. (1999). Electronic Structure and Optical Limiting Behavior of Carbon Nanotubes. Phys. Rev. Lett..

[114] Schafer J., Ristein J., Graupner R., Ley L., Stephan U., Frauenheim T., Veersamy V.S., Amaratunga G.A.J., Weiler M., Ehrhardt H. (1996). Photoemission study of amorphous carbon modifications and comparison with calculated densities of states. Phys. Rev. B.

[115] Umari P., Petrenko O., Taioli S., de Souza M.M. (2012). Communication: Electronic band gaps of semiconducting zig-zag carbon nanotubes from many-body perturbation theory calculations. J. Chem. Phys..

[116] Soncini C., Bondino F., Magnano E., Bhardwaj S., Kumar M., Cepek C., Pedio M. (2021). Electronic properties of carbon nanotubes as detected by photoemission and inverse photoemission. Nanotechnology.

[117] Suzuki S., Bower C., Kiyokura T., Nath K.G. (2001). Photoemission spectroscopy of single-walled carbon nanotube bundles. J. Electron Spectrosc. Relat. Phenom..

[118] Schiessling J., Kjeldgaard L., Rohmund F., Falk L.K.L., Campbell E.E.B., Nordgren J., Bruhwiler P.A. (2003). Synchrotron radiation study of the electronic structure of multiwalled carbon nanotubes. J. Phys. Condens. Matter.

[119] Li Z., Zheng L., Yan W., Pan Z., Wei S. (2009). Spectroscopic Characteristics of Differently Produced Single-Walled Carbon Nanotubes. ChemPhysChem.

[120] Bennich P., Puglia C., Bruhwiler P.A., Nilsson A., Maxwell A.J., Sandell A., Martensson N., Rudolf P. (1999). Photoemission study of K on graphite. Phys. Rev. B.

[121] Bohm D., Pines D. (1951). A Collective Description of Electron Interactions I Magnetic Interactions. Phys. Rev..

[122] Ayala P., Miyata Y., de Blauwe K., Shiozawa H., Feng Y., Yanagi K., Kramberger C., Silva S.R.P., Follath R., Kataura H. (2009). Disentanglement of the electronic properties of metallicity-selected single-walled carbon nanotubes. Phys. Rev. B.

[123] Castrucci P., Scarselli M., de Crescenzi M., el Khahanib A., Rosei F. (2010). Probing the electronic structure of carbon nanotubes by nanoscale spectroscopy. Nanoscale.

[124] Kramberger C., Rauf H., Shiozawa H., Knupfer M., Buchner B., Pichler T., Batchelor D., Kataura H. (2007). Unraveling van Hove singularities in X-ray absorption response of single-wall carbon nanotubes. Phys. Rev. B.

[125] Shearer C.J., Yu L.P., Blanch A.J., Zheng M., Andersson G.G., Shapter J.G. (2019). Broadening of van Hove Singularities Measured by Photoemission Spectroscopy of Single- and Mixed-Chirality Single-Walled Carbon Nanotubes. J. Phys. Chem. C.

[126] Okpalugo T.I.T., Papakonstantinou P., Murphy H., McLaughlin J., Brown N.M.D. (2005). High resolution XPS characterization of chemical functionalised MWCNTs and SWCNTs. Carbon.

[127] Wespasnick K.A., Smith B.A., Bitter J.L., Fairbrother D.H. (2010). Chemical and structural characterization of carbon nanotube surfaces. Anal. Bioanal. Chem..

[128] Xia W., Wang Y., Bergstraßer R., Kundu S., Muhler M. (2007). Surface characterization of oxygen-functionalized multi-walled carbon nanotubes by high-resolution X-ray photoelectron spectroscopy and temperature-programmed desorption. Appl. Surf. Sci..

[129] Balasubramanian K., Burghard M. (2005). Chemically functionalized carbon nanotubes. Small.

[130] Zhao J., Park H., Han J., Lu J.P. (2004). Electronic Properties of Carbon Nanotubes with Covalent Sidewall Functionalization. J. Phys. Chem. B.

[131] Lim S.H., Lin J., Liu L., Pan H., Pan H.L., Ji W., Feng Y.P., Shen Z. (2008). Functionalization effect on the electronic properties of single walled carbon nanotubes. Funct. Mater. Lett..

[132] Duclaux L. (2002). Review of the doping of carbon nanotubes (multiwalled and single-walled). Carbon.

[133] Park Y.R., Ko M.J., Song Y.-H., Lee C.J. (2013). Surface electronic structure of nitrogen-doped semiconducting single-walled carbon nanotube networks. J. Appl. Phys..

[134] Dettlaff-Weglikowsk U., Kim G., Bulusheva L.G., Roth S. (2011). Modification of the electronic structure in single-walled carbon nanotubes with aromatic amines. Phys. Status Solidi B.

[135] Dementjev A.P., Eletskii A.V., Maslakov K.I., Rakov E.G., Sukhoverhov V.F., Naumkin A.V. (2006). Fluorination of Carbon Nanostructures and Their Comparative Investigation by XPS and XAES Spectroscopy. Fuller. Nanotub. Carbon Nonstruct..

[136] Osawa E. (1970). Superaromaticity. Kagaku.

[137] Castro E., Garcia A.H., Zavala G., Echegoyen L. (2017). Fullerenes in biology and medicine. J. Mater. Chem. B.

[138] Lieber C.M., Chen C.-C. (1994). Preparation of Fullerenes and Fullerene-Based Materials. Solid State Phys..

[139] Hare J.P., Kroto H.W., Taylor R. (1991). Preparation and UV/visible spectra of fullerenes C60 and C70. Chem. Phys. Lett..

[140] Taylor R., Langley G.J., Kroto H.W., Walton D.R.M. (1993). Formation of C60 by pyrolysis of naphthalene. Nature.

[141] Howard J.B., McKinnon J.T., Makarovsky Y., Lafleur A.L., Johnson M.E. (1991). Fullerenes C60 and C70 in flames. Nature.

[142] Kroto H.W. (1988). Space, Stars, C60, and Soot. Science.

[143] Stalling D.L., Kuo K.C., Guo C.Y., Saim S. (1993). Separation of Fullerenes C60, C70, and C76-84 on Polystyrene Divinylbenzene Columns. J. Liq. Chromatogr..

[144] Guldi D.M., Illescas B.M., Atienza C.M., Wielopolski M., Martin N. (2009). Fullerene for organic electronics. Chem. Soc. Rev..

[145] Prato M. (1997). Fullerene chemistry for materials science applications. J. Mater. Chem..

[146] Martin N. (2006). New challenges in fullerene chemistry. Chem. Commun..

[147] Hebard A.F., Rosseinski M.J., Haddon R.C., Murphy D.W., Glarum S.H., Palstra T.T.M., Ramirez A.P., Kortan A.R. (1991). Superconductivity at 18 K in potassium-doped C60. Nature.

[148] Holczer K., Klein O., Huang S.-M., Kaner R.B., Fu K.-J., Whetten R.L., Diederich F. (1991). Alkali-Fulleride Superconductors: Synthesis, Composition, and Diamagnetic Shielding. Science.

[149] Allemand P.M., Khemani K.C., Koch A., Wudl F., Holczer K., Donovan S., Gruner G., Thompson J.D. (1991). Organic Molecular Soft Ferromagnetism in a Fullerene C60. Science.

[150] Tutt L.W., Kost A. (1992). Optical limiting performance of C60 and C70 solutions. Nature.

[151] Pana Y., Liu X., Zhang W., Liu Z., Zeng G., Shao B., Liang Q., He Q., Yuan X., Huang D. (2020). Advances in photocatalysis based on fullerene C60 and its derivatives: Properties, mechanism, synthesis, and applications. Appl. Catal. B.

[152] Wen C., Li J., Kitazawa K., Aida T., Honma I., Komiyama H., Yamada K. (1992). Electrical conductivity of a pure C60 single crystal. Appl. Phys. Lett..

[153] Mort J., Ziolo R., Machonkin M., Huffman D.R., Ferguson M.I. (1991). Electrical conductivity studies of undoped solid films of C60, C70. Chem. Phys. Lett..

[154] Gueorguiev G., Pacheco J.M., Tomanek D. (2004). Quantum Size Effects in the Polarizability of Carbon Fullerenes. Phys. Rev. Lett..

[155] Matveeva L.A., Venger E.F., Kolyadina E.Y., Neluba P.L. (2017). Quantum-size effects in semiconductor heterosystems. Semicond. Phys. Quantum Electron. Optoelectron..

[156] Haddon R.C., Pasquarello A. (1994). Magnetism of carbon clusters. Phys. Rev. B.

[157] Reinke P., Feldermann H., Oelhafen P. (2003). C60 bonding to graphite and boron nitride surfaces. J. Chem. Phys..

[158] Yu J., Kalia R., Vashishta P. (1993). Phonon dispersion and density of states of solid C60. Appl. Phys. Lett..

[159] Hua X., Cagin T., Che J., Goddard W.A. (2000). QM(DFT) and MD studies on formation mechanisms of C60 fullerenes. Nanotechnology.

[160] Hasegawa M., Ohno K. (1996). Density functional theory for the phase diagram of rigid C60 molecules. Phys. Rev. E.

[161] Feng M., Zhao J., Petek H. (2008). Atomlike Hollow-Core-Bound Molecular Orbitals of C60. Science.

[162] Bilan S., Zotti L.A., Pauly F., Cuevas J.C. (2012). Theoretical study of the charge transport through C60-based single-molecule junctions. Phys. Rev. B.

[163] Endo K., Koizumi S., Otsuka T., Ida T., Morohashi T., Onoe J., Nakao A., Kurmaev E.Z., Moewes A., Chong D.P. (2003). Analysis of Electron Spectra of Carbon Allotropes (Diamond, Graphite, Fullerene) by Density Functional Theory Calculations Using the Model Molecules. J. Phys. Chem. A.

[164] Kalb W.L., Haas S., Krellner C., Mathis T., Batlogg B. (2010). Trap density of states in small-molecule organic semiconductors: A quantitative comparison of thin-film transistors with single crystals. Phys. Rev. B.

[165] Yogev S., Matsubara R., Nakamura M., Zschieschang U., Klauk H., Rosenwaks Y. (2013). Fermi Level Pinning by Gap States in Organic Semiconductors. Phys. Rev. Lett..

[166] Bussolotti F., Yang J., Hiramoto M., Kaji T., Kera S., Ueno N. (2015). Direct detection of density of gap states in C60 single crystals by photoemission spectroscopy. Phys. Rev. B.

[167] Hwang J., Wan A., Khan A. (2009). Energetics of metal-organic interfaces: New experiments and assessment of the field. Mater. Sci. Eng. R.

[168] Wang C., Liu X., Wang C., Kauppi J., Gao Y. (2015). Electronic structure evolution in doping of fullerene (C60) by ultra-thin layer molybdenum trioxide. J. Appl. Phys..

[169] Weibo Y., Seifermann S.M., Pierrat P., Brase S. (2015). Synthesis of highly functionalized C60 fullerene derivatives and their applications in material and life sciences. Org. Biomol. Chem..

[170] Rašović I. (2017). Water-soluble fullerenes for medical applications. Mater. Sci. Technol..

[171] Lin H.-L., Yutaka M. (2018). Functionalization of fullerene through fullerene cation intermediates. Chem. Commun..

[172] Alonso A.M., Bonifazi D., Prato M., Dai L. (2006). Functionalization and applications of fullerene-Chap 7. Carbon Nanotechnologies.

[173] Fukuzumi S. (2017). Nanocarbons as Electron Donors and Acceptors in Photoinduced Electron-Transfer Reactions. ECS J. Solid State Sci. Technol..

[174] Tkachenko N.V., Rantala L., Tauber A.Y., Helaja J., Hynninen P.H., Lemmetyinen H. (1999). Photoinduced Electron Transfer in Phytochlorin−Fullerene Dyads. J. Am. Chem. Soc..

[175] Koeppe R., Sariciftci N.S. (2005). Photoinduced charge and energy transfer involving fullerene derivatives. Photochem. Photobiol. Sci..

[176] Mikami K., Matsumoto S., Tonoi T., Okubo Y., Suenobu T., Fukuzumi S. (1998). Solid state photochemistry for fullerene functionalization: Solid state photoinduced electron transfer in the Diels-Alder reaction with anthracenes. Tetrahedron Lett..

[177] Mroz P., Tegos G.P., Gali H., Wharton T., Sarna T., Hamblin M.R. (2007). Photodynamic therapy with fullerenes. Photochem. Photobiol. Sci..

[178] da Ros T., Prato M. (1999). Medicinal chemistry with fullerenes and fullerene derivatives. Chem. Commun..

[179] Zygouri P., Spyrou K., Mitsari E., Barrio M., Macovez R., Patila M., Stamatis H., Verginadis I.I., Velalopoulou A.P., Evangelou A.M. (2020). A facile approach to hydrophilic oxidized fullerenes and their derivatives as cytotoxic agents and supports for nanobiocatalytic systems. Sci. Rep..

[180] Li B., Zhen J., Wan Y., Lei X., Jia L., Wu X., Zeng H., Chen M., Wang G.-W., Yang S. (2020). Steering the electron transport properties of pyridine-functionalized fullerene derivatives in inverted perovskite solar cells: The nitrogen site matters. J. Mater. Chem. A.

[181] Liu J., Wang Y., Jiang P., Tu G. (2020). Functionalized Amphiphilic Diblock Fullerene Derivatives as a Cathode Buffer Layer for Efficient Inverted Organic Solar Cells. ACS Omega.

[182] Nyga A., Motya R., Bussetti G.L., Calloni A., Jagadeeshb M.S., Pluczyk-Malek S., Data P., Blacha-Grzechnik A. (2020). Electrochemically deposited poly(selenophene)-fullerene photoactive layer:Tuning of the spectroscopic properties towards visible light-driven generation of singlet oxygen. Appl. Surf. Sci..

[183] Liu Z., Koshino M., Suenaga K., Mrzel A., Kataura H., Iijima S. (2006). Transmission Electron Microscopy Imaging of Individual Functional Groups of Fullerene Derivatives. Phys. Rev. Lett..

[184] Balaz P. (2008). High-energy milling-Chap 2. Mechanochemistry in Nanoscience and Minerals Engineering.

[185] Boudou J.P., Curmi P.A., Lelezko F., Wrachtrup J., Aubert P., Sennour M., Balasubramanian G., Reuter R., Thorel A., Gaffet E. (2009). High yield fabrication of fluorescent nanodiamonds. Nanotechnology.

[186] Guan S., Peng F., Liang H., Fan C., Tan L., Wang Z., Zhang Y., Zhang J., Yu H., He D. (2018). Fragmentation and stress diversification in diamond powder under high pressure. J. Appl. Phys..

[187] Kumar A., Lin P.A., Xue A., Hao B., Yap Y.K., Sankaran M. (2013). Formation of nanodiamonds at near-ambient conditions via microplasma dissociation of ethanol vapour. Nat. Commun..

[188] Tzeng Y.-K., Zhang J.L., Lu H., Ishiwata H., Dahl J., Carlson R.M.K., Yan H., Schreiner P.R., Vuckovic J., Shen Z.-X. (2017). Vertical-Substrate MPCVD Epitaxial Nanodiamond Growth. Nano Lett..

[189] Amans D., Chenus A.C., Ledoux G., Dujardin C., Reynaud C., Sublemontier O., Masenelli-Varlot K., Guillois O. (2009). Nanodiamond synthesis by pulsed laser ablation in liquids. Diam. Relat. Mater..

[190] Bazzanella N., Cazzanelli M., Dorigoni C., Bifone A., Miotello A. (2018). The modeling and synthesis of nanodiamonds by laser ablation of graphite and diamond-like carbon in liquid-confined ambient. Appl. Phys. A.

[191] Shenderova O., Koscheev A., Zaripov N., Petrov I., Skryabin Y., Dektov P., Turner S., van Tendeloo G. (2011). Surface Chemistry and Properties of Ozone-Purified Detonation Nanodiamonds. J. Phys. Chem. C.

[192] Popov V. (2021). Several Aspects of Application of Nanodiamonds as Reinforcements for Metal Matrix Composites. Appl. Sci..

[193] Lesiaka P., Kover L., Toth J., Zemek J., Jiricek P., Kromka A., Rangama N. (2018). C sp2/sp3 hybridisations in carbon nanomaterials-XPS and (X)AES study. Appl. Surf. Sci..

[194] Xie Y., Xie W.G., Gong L., Zhang W.H., Chen S.H., Zhang Q.Z., Chen J. (2010). Surface characterization on graphitization of nanodiamond powder annealed in nitrogen ambient. Surf. Interface Anal..

[195] Speranza G., Laidani N. (2004). Measurement of the relative abundance of sp2 and sp3 hybridised atoms in carbon-based materials by XPS: A critical approachPart I. Diam. Relat. Mater..

[196] Speranza G., Laidani N. (2004). Measurement of the relative abundance of sp2 and sp3 hybridised atoms in carbon-based materials by XPS: A critical approachPart II. Diam. Relat. Mater..

[197] Osswald S., Yushin G., Mochalin V., Kucheyev S.O. (2006). Control of sp2/sp3 Carbon Ratio and Surface Chemistry of Nanodiamond Powders by Selective Oxidation in Air. J. Am. Chem. Soc..

[198] Butenko Y.V., Krishnamurthy S., Chakraborty A.K., Kuznetsov V.L., Dhanak V.R., Hunt M.R.C., Siller L. (2005). Photoemission study of onionlike carbons produced by annealing nanodiamonds. Phys. Rev. B.

[199] Yeganeh M., Coxon P.R., Brieva A.C., Dhanak V.R., Siller L., Butenko Y.V. (2007). Atomic hydrogen treatment of nanodiamond powder studied with photoemission spectroscopy. Phys. Rev. B.

[200] Cui J.B., Ristein J., Ley L. (1998). Electron Affinity of the Bare and Hydrogen Covered Single Crystal Diamond (111) Surface. Phys. Rev. Lett..

[201] Wilson T.B., Walton J.S., Beamson G. (2001). Analysis of chemical vapour deposited diamond films by X-ray photoelectron spectroscopy. J. Electron Spectrosc. Relat. Phenom..

[202] Maier F., Riedel M., Mantel B., Riestein J., Ley L. (2000). Origin of Surface Conductivity in Diamond. Phys. Rev. Lett..

[203] Zhu Y., Li J., Zhang Y., Yang X., Chen N., Sun Y., Zhao Y., Fan C., Huang Q. (2012). The Biocompatibility of Nanodiamonds and Their Application in Drug Delivery Systems. Theranostics.

[204] Kozakov A.T., Kochur A.G., Kumar N., Panda K., Nikolskii A.V., Sidashov A.V. (2021). Determination of sp2 and sp3 phase fractions on the surface of diamond films from C1s, valence band X-ray photoelectron spectra and CKVV X-ray-excited Auger spectra. Appl. Surf. Sci..

[205] Bianconi A., Hagstrom S.B.M., Babrach R.Z. (1977). Photoemission studies of graphite high-energy conduction-band and valence-band states using soft-X-ray synchrotron radiation excitation. Phys. Rev. B.

[206] Kundu R., Mishra P., Sekhar B.R., Maniraj M., Barman S.R. (2012). Electronic structure of single crystal and highly oriented pyrolytic graphite from ARPES and KRIPES. Phys. B.

[207] Bandis C., Pate B.B. (1995). Electron Emission Due to Exciton Breakup from Negative Electron Affinity Diamond. Phys. Rev. Lett..

[208] Ristein J., Stein W., Ley L. (1997). Defect Spectroscopy and Determination of the Electron Diffusion Length in Single Crystal Diamond by Total Photoelectron Yield spectroscopy. Phys. Rev. Lett..

[209] James M., Fogarty F., Zulkharnay R., Fox N.A., May P.W. (2020). A review of surface functionalisation of diamond for thermionic emission applications. Carbon.

[210] Nemanich R.J., Carlisle J.A., Hirata A. (2014). CVD diamond: Research, applications, and challenges. MRS Bull..

[211] Raty J.-Y., Galli G., Bosted C., van Buuren T.W., Terminello L. (2003). Quantum Confinement and Fullerenelike Surface Reconstructions in Nanodiamonds. Phys. Rev. Lett..

[212] Ristein J. (2005). Diamond surfaces: Familiar and amazing. Appl. Phys. A.

[213] Diederich L., Kuttel O.M., Ruffieux P., Pillo T., Aebi P., Schlapbach L. (1998). Photoelectron emission from nitrogen- and boron-doped diamond(100) surfaces. Surf. Sci..

[214] Zhirnov V.V., Shenderova O.A., Jaeger D.L., Tyler T., Areshkin D.A., Brenner D.W., Hren J.J. (2004). Electron emission properties of detonation nanodiamonds. Phys. Solid State.

[215] Maillard-Schaller E., Kuettel O.M., Diederich L., Schlapbach L., Zhirnov V., Belobrov P.I. (1999). Surface properties of nanodiamond films deposited by electrophoresis on Si(100). Diam. Relat. Mater..

[216] Shenderova O., Nunn N. (2017). Chapter 2-Production and purification of nanodiamonds. Nanodiamonds-Advanced Material Analysis, Properties and Applications-Micro and Nano Technologies.

[217] Gismondi A., Reina G., Orlanducci S., Mizzoni F., Gay S., Terranova M.L., Canini A. (2015). Nanodiamonds coupled with plant bioactive metabolites: A nanotech approach for cancer therapy. Biomaterials.

[218] Tinwala H., Wairkar S. (2019). Production, surface modification and biomedical applications of nanodiamonds: A sparkling tool for theranostics. Mater. Sci. Eng. C.

[219] Neburkova J., Vavra J., Cigler P. (2017). Coating nanodiamonds with biocompatible shells for applications in biology and medicine. Curr. Opin. Solid State Mater. Sci..

[220] Mona J., Kuo C.-J., Perevedentseva E., Priezzhev A.V., Cheng C.-L. (2013). Adsorption of human blood plasma on nanodiamond and its influence on activated partial thromboplastin time. Diam. Relat. Mater..

[221] Li H.-C., Hsieh F.-J., Chen C.-P., Chang M.-Y., Hieh P.C.H., Chen C.-C., Hung S.-U., Wu C.-C., Chang H.-C. (2013). The hemocompatibility of oxidized diamond nanocrystals for biomedical applications. Sci. Rep..

[222] Tsai L.-W., Lin Y.-C., Perevedentseva E., Lugovtsov A., Priezzhev A., Cheng C.-L. (2016). Nanodiamonds for medical applications: Interaction with blood in vitro and in vivo. Int. J. Mol. Sci..

[223] Edgington R., Spillane K.M., Papageorgiou G., Wray W., Ishiwata H., Labarca M., Leal-Ortiz S., Reid G., Webb M., Foord J. (2018). Functionalisation of Detonation Nanodiamond for Monodispersed, Soluble DNA-Nanodiamond Conjugates Using Mixed Silane Bead-Assisted Sonication Disintegration. Sci. Rep..

[224] Whitlow J., Pacelli S., Paul A. (2017). Multifunctional nanodiamonds in regenerative medicine: Recent advances and future directions. J. Control. Release.

[225] Hajiali F., Shojaei A. (2016). Silane functionalization of nanodiamond for polymer nanocomposites-effect of degree of silanization. Colloids Surf. A.

[226] Zheng W.-W., Hsieh Y.-H., Chiu Y.-C., Cai S.-J., Cheng C.-L., Chen C. (2009). Organic functionalization of ultradispersed nanodiamond: Synthesis and applications. J. Mater. Chem..

[227] Krueger A., Ozawa M., Jarre G., Liang Y., Stegk J., Lu L. (2007). Deagglomeration and functionalisation of detonation diamond. Phys. Status Solid A.

[228] Krueger A., Lang D. (2012). Functionality is Key: Recent Progress in the Surface Modification of Nanodiamond. Adv. Funct. Mater..

[229] Barzegar M., Olia A., Donnelly P.S., Hollenberg L.C.L., Mulvaney P., Simpson D.A. (2021). Advances in the Surface Functionalization of Nanodiamonds for Biological Applications: A Review. ACS Appl. Nano Mater..

[230] Reina G., Zhao L., Bianco A., Komatsu N. (2019). Chemical Functionalization of Nanodiamonds: Opportunities and Challenges Ahead. Angew. Chem. Int. Ed..

[231] Shenderova O., Panich A.M., Moseenkov S.I., Hens S.C., Kuznetsov V.L., Vieth H.-M. (2011). Hydroxylated Detonation Nanodiamond: FTIR, XPS, and NMR Studies. J. Phys. Chem. C.

[232] Sharin P., Sivtseva A.V., Popov V.I. (2021). X-ray Photoelectron Spectroscopy of Nanodiamonds Obtained by Grinding and Detonation Synthesis. Technol. Phys..

[233] Arnault J.C. (2018). X-ray Photoemission Spectroscopy applied to nanodiamonds: From surface chemistry to in situ reactivity. Diam. Relat. Mater..

[234] Minati L., Torrengo S., Maniglio D., Migliaresi C., Speranza G. (2012). Luminescent graphene quantum dots from oxidized multi-walled carbon nanotubes. Mater. Chem. Phys..

[235] Kahn Z., Tsai T., Taniguchi K., Watanabe A., Zettl F. (2016). Imaging electrostatically confined Dirac fermions in graphene quantum dots. Nat. Phys..

[236] Gutierrez C., Brown L., Kim C.J., Park J., Pasupathy A.N. (2016). Klein tunnelling and electron trapping in nanometre-scale graphene quantum dots. Nat. Phys..

[237] Bai K.K., Zhou J.J., Wei Y.C., Qiao J.B., Liu Y.W., Liu H.W., Jiang H. (2018). Generating atomically sharp p-n junctions in graphene and testing quantum electron optics on the nanoscale. Phys. Rev. B.

[238] Jiang Y., Mao J., Moldovan D., Masir M.R., Li G., Watanabe K., Taniguchi T., Peeters F.M., Andrei E.Y. (2017). Tuning a circular p-n junction in graphene from quantum confinement to optical guiding. Nat. Nanotechnol..

[239] Fu Z.Q., Bai K.K., Ren Y.N., Zhou J.J., He L. (2020). Coulomb interaction in quasibound states of graphene quantum dots. Phys. Rev. B.

[240] Yuan F., Yuan T., Sui L., Wang Z., Xi Z., Li Y., Li X., Fan L., Tan Z., Chen A. (2018). Engineering triangular carbon quantum dots with unprecedented narrow bandwidth emission for multicolored LEDs. Nat. Commun..

[241] Yuan F., Wang Z., Li X., Li Y., Tan Z., Fan L., Yang S. (2017). Bright multicolor bandgap fluorescent carbon quantum dots for electroluminescent light-emitting diodes. Adv. Mater..

[242] Carolan D., Rocks C., Padmanaban D.B., Maguire P., Svrcekb V., Mariotti D. (2017). Environmentally friendly nitrogen-doped carbon quantum dots for next generation solar cells. Sustain. Energy Fuels.

[243] Yang S.T., Wang X., Wang H.F., Lu F.S., Luo P.J.G., Cao L., Meziani M.J., Liu J.H., Chen M., Huang Y.P. (2009). Carbon Dots as Nontoxic and High-Performance Fluorescence Imaging Agents. J. Phys. Chem. C.

[244] Wang X., Cao L., Lu F.S., Meziani M.J., Li H., Qi G., Zhou B., Harruff B.A., Kerramec F., Sun Y.P. (2009). Photoinduced electron transfers with carbon dots. , Chem. Commun..

[245] Hu S.L., Niu K.Y., Sun J., Yang J., Zhao N.Q., Du X.W. (2009). One-step synthesis of fluorescent carbon nanoparticles by laser irradiation. J. Mater. Chem..

[246] Zhao Q.L., Zhang Z.L., Huang B.H., Peng J., Zhang M., Pang D.W. (2008). Facile preparation of low cytotoxicity fluorescent carbon nanocrystals by electrooxidation of graphite. Chem. Commun..

[247] Li H.T., He X.D., Kang Z.H., Huang H., Liu Y., Liu J.L., Lian S.Y., Tsang C.H., Yang X.B., Lee S.T. (2010). Water-Soluble Fluorescent Carbon Quantum Dots and Photocatalyst Design, Angew. Angew. Chem. Int. Ed..

[248] Ming H., Ma Z., Liu Y., Pan K.M., Yu H., Wang F., Kang Z.H. (2012). Large scale electrochemical synthesis of high quality carbon nanodots and their photocatalytic property. Dalton Trans..

[249] Gao R., Wu Z., Wang L., Liu J., Deng Y., Xiao Z., Fang J., Liang Y. (2020). Green Preparation of Fluorescent Nitrogen-Doped Carbon Quantum Dots for Sensitive Detection of Oxytetracycline in Environmental Samples. Nanomaterials.

[250] Luo X., Bai P., Wang X., Zhao G., Feng J., Rend H. (2019). Preparation of nitrogen-doped carbon quantum dots and its application as a fluorescent probe for Cr(vi) ion detection. New J. Chem..

[251] Jing S., Zhao Y., Sun R.-C., Zhong L., Peng X. (2019). Facile and High-Yield Synthesis of Carbon Quantum Dots from Biomass-Derived Carbons at Mild Condition. ACS Sustain. Chem. Eng..

[252] Nguyen H.A., Srivastava I., Pan D., Gruebele M. (2020). Unraveling the Fluorescence Mechanism of CQDs with Sub-Single-Particle Resolution. ACS Nano.

[253] Deng Y., Chen M., Chen G., Zou W., Zhao Y., Zhang H., Zhao Q. (2021). Visible-Ultraviolet Upconversion Carbon Quantum Dots for Enhancement of the Photocatalytic Activity of Titanium Dioxide. ACS Omega.

[254] Papagiannouli I., Patanen M., Blanchet V., Bozek J.D., Villa M.D., Huttula M., Kokkonen E., Lamour E., Mevel E., Pelimanni E. (2018). Depth Profiling of the Chemical Composition of Free-Standing Carbon Dots Using Xâ€‘ray Photoelectron Spectroscopy. J. Phys. Chem. C.

[255] Chawla K.K. (1998). Carbon Fibers in Composite Materials.

[256] Petersen R. (2016). Carbon Fiber Biocompatibility for Implants. Fibers.

[257] Park S.-J. (2018). Carbon Fibers.

[258] Al-Saleh M.H., Sundararaj U. (2011). Review of the mechanical properties of carbon nanofiber/polymer composites. Compos. A.

[259] Shamsuddin S.R., Ho K.K.C., Lee K.H., Hodgkinson J.M., Bismarck A., Nicolais L., Borzacchiello A. (2012). Carbon Fiber: Properties, Testing, and analysis. Wiley Encyclopedia of Composites.

[260] Zou G., Zhang D., Dong C., Li H., Xiong K., Fei L., Qian Y. (2006). Carbon nanofibers: Synthesis, characterization, and electrochemical properties. Carbon.

[261] Rodriguez N.M. (1993). A review of catalytically grown carbon nanofibres. J. Mater. Res..

[262] Manafi S.A., Badiee S.H. (2008). Production of carbon nanofibers using a CVD method with lithium fluoride as a supported cobalt catalyst. Res. Lett. Mater. Sci..

[263] Tibbets G.G., Gorkiewicz D.W., Alig R.L. (1993). A new reactor for growing carbon-fibers from liquid-phase and vapor-phase hydrocarbons. Carbon.

[264] Ge M., Sattler K. (1994). Observation of fullerene cones. Chem. Phys. Lett..

[265] Zhao T., Kvande I., Yu Y., Ronning M., Holmen A., Chen D. (2011). Synthesis of Platelet Carbon Nanofiber/Carbon Felt Composite on in Situ Generated Ni-Cu Nanoparticles. J. Phys. Chem. C.

[266] Kim Y.A., Hayashi T., Endo M., Dresselhaus M.S., Vajtai R. (2013). Carbon nanofibers. Handbook of Nanomaterials.

[267] Huang H., Kajiura H., Murakami Y., Ata M. (2003). Metal sulfide catalyzed growth of carbon nanofibers and nanotubes. Carbon.

[268] Kajiura H., Huang H., Tsutsui S., Murakami Y., Miyakoshi M. (2002). High purity fibrous carbon deposit on the anode surface in hydrogen DC arc-discharge. Carbon.

[269] Inagaki M., Yang Y., Kang F. (2012). Carbon nanofibers prepared via electrospinning. Adv. Mater..

[270] Zhang L., Aboagye A., Kelkar A., Lai C., Fong H. (2014). A review: Carbon nanofibers from electrospun polyacrylonitrile and their applications. J. Mater. Sci..

[271] Omoriyekomwan J.E., Tahmasebi A., Dou J., Wang R. (2021). A review on the recent advances in the production of carbon nanotubes and carbon nanofibers via microwave-assisted pyrolysis of biomass. Fuel Proc. Technol..

[272] Zhang J., Tahmasebi A., Omoriyekomwan J.E., Yu J. (2018). Direct synthesis of hollow carbon nanofibers on bio-char during microwave pyrolysis of pine nut shell. J. Anal. Appl. Pyrolysis.

[273] Bengtsson A., Bengtsson J., Sedin M., Sjoholm E. (2019). Carbon Fibers from Lignin-Cellulose Precursors: Effect of Stabilization Conditions. ACS Sustain. Chem. Eng..

[274] Nuryantini A.Y., Rahayu F., Mahen E.C.S., Sawitri A., Nuryadin B.W. (2018). Synthesis of activated carbon fiber from pyrolyzed cotton for adsorption of fume pollutants. J. Phys. Conf. Ser..

[275] Gardner S., Singamsetty C.S.K., Booth G.L., He G.-R., Pittman J.C.U. (1995). Surface characterization of carbon fibers using angle-resolved XPS and ISS. Carbon.

[276] Akia M., Salinas N., Luna S., Medina E., Valdez A., Lopez J., Ayala J., Alcoutlabi M., Lozano K. (2019). In situ synthesis of Fe3O4-reinforced carbon fiber composites as anodes in lithium-ion batteries. J. Mater. Sci..

[277] He G., Song Y., Chen S., Wang L. (2018). Porous carbon nanofiber mats from electrospun polyacrylonitrile/polymethylmethacrylate composite nanofibers for super-capacitor electrode materials. J. Mater. Sci..

[278] Li T.F., Lv Y.L., Su J.H., Wang Y., Yang Q., Zhang Y.W., Zhou J.C., Xu L., Sun D.M., Tang Y.W. (2017). Anchoring CoFe2O4 nanoparticles on N-doped carbon nanofibers for high-performance oxygen evolution reaction. Adv. Sci..

[279] Chiang Y., Wu C., Chen Y. (2020). Effects of activation on the properties of electrospun carbon nanofibers and their adsorption performance for carbon dioxide. Sep. Purif. Technol..

[280] Lindsay B., Abel M.-L., Watts J.F. (2007). A study of electrochemically treated PAN based carbon fibres by IGC and XPS. Carbon.

[281] Boukhvalov D.W., Zhidkov I.S., Kiryakov A., Menendez J.L., Garcia L.F., Kukharenko A.I., Cholakh S.O., Zatsepin A.F., Kurmaev E.Z. (2021). Unveiling the Atomic and Electronic Structure of StackedCup Carbon Nanofibers. Nanoscale Res. Lett..

[282] Yadava D., Aminib F., Ehrmann A. (2020). Recent advances in carbon nanofibers and their applications–A review. Eur. Polym. J..

[283] Guo Q., Liu D., Zhang X., Li L., Hou H., Niwa O., You T. (2014). Pd-Ni Alloy Nanoparticle/Carbon Nanofiber Composites:Preparation, Structure, and Superior Electrocatalytic Properties for Sugar Analysis. Anal. Chem..

[284] Guo L., Wan K., Liu B., Wang Y., Wei G. (2021). Recent advance in the fabrication of carbon nanofiber-based composite materials for wearable devices. Nanotechnology.

[285] Zhou X., Wang Y., Gong C., Liu B., Wei G. (2020). Production, structural design, functional control, and broad applications of carbon nanofiber-based nanomaterials: A comprehensive review. Chem. Eng. J..

